# Thermally Reconstructed Ru/La‐Co_3_O_4_ Nanosheets with Super Thermal Stability for Catalytic Combustion of Light Hydrocarbons: Induced Surface LaRuO_3_ Active Phase

**DOI:** 10.1002/advs.202414919

**Published:** 2025-03-16

**Authors:** Biao Gao, Wei Deng, Hangqi Xia, Kai Shan, Li Wang, Boyuan Qiao, Qiang Niu, Aiyong Wang, Yun Guo, Wangcheng Zhan, Yanglong Guo, Qiguang Dai

**Affiliations:** ^1^ State Key Laboratory of Green Chemical Engineering and Industrial Catalysis Research Institute of Industrial Catalysis School of Chemistry and Molecular Engineering East China University of Science and Technology Shanghai 200237 P. R. China; ^2^ School of Optoelectronic Materials and Technology Jianghan University Wuhan 430056 P. R. China; ^3^ Electric Power and Metallurgy Group Co. Ltd. Ordos Inner Mongolia 016064 P. R. China; ^4^ Zhejiang Wild Wind Pharmaceutical Co. Ltd. Zhejiang 322105 P. R. China

**Keywords:** catalytic combustion, cobalt oxide, LaRuO3 perovskite, light hydrocarbons, thermal stability

## Abstract

Addressing the thermal deactivation of catalysts remains critical for light hydrocarbons (LHs) combustion. This study develops Ru/La‐Co_3_O_4_ nanosheets combining RuO_x_ and La‐doped Co_3_O_4_, demonstrating exceptional high‐temperature stability. The individual introduction of Ru or La significantly promoted the catalytic activity of Co_3_O_4_, but a severe thermal deactivation is still inevitable. Remarkably, the co‐decoration of Ru and La brought a prominent resistance to high‐temperature, the aged Ru/La‐Co_3_O_4_ at 750 °C for 4 h presented a better activity than the fresh catalyst and the T_C3‐90_ instead decreased by 11 °C, even only increased by 1 °C after aging 100 h and 15 °C for 200 h. Systematic studies revealed that the co‐presence of La and Ru enhanced the resistance to sintering of Co_3_O_4_ and promoted the migration of the lattice oxygen, moreover, the high‐temperature induced the formation of LaRuO_3_ perovskite through the reaction of RuO_x_ with the exsolved La from Co_3_O_4_. LaRuO_3_ phase with excellent redox ability and thermal stability presented a superior activity for catalytic combustion of LHs and suppressed the leaching of Ru species in an oxidizing atmosphere at high temperature. This work contributed to the design of catalysts especially Ru based catalysts for the stable elimination of LHs emissions under high temperature conditions.

## Graphical abstract

The high‐temperature induced the exsolution of La from La‐Co_3_O_4_ lattice and facilitated the in situ formation of highly active LaRuO_3_ perovskite through a solid‐phase reaction with surface RuO_x_. This process significantly enhanced the thermal and hydrothermal stability of Ru/La‐Co_3_O_4_ for light hydrocarbons (LHs) oxidation, providing an innovative strategy to stabilize the Ru species under high‐temperature oxidizing conditions.

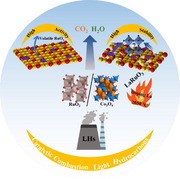

## 1 Introduction

Light hydrocarbons (LHs), hydrocarbons with low molecular weight such as methane, ethane, and propane, have been regarded as attractive chemical feedstocks and fuels, for examples, the dehydrogenation or oxidative dehydrogenation of propane or ethane into olefins, the oxychlorination of methane into platform materials for various valuable chemicals, and fuels as compressed natural gas (CNG) or liquefied natural gas (LNG) engines.^[^ ^1^ ^]^ However, these unreacted or unburned and by‐producing LHs as potent greenhouse gas were directly emitted into the atmospheric environment and caused serious air pollution. Practically, the global warming potential (GWP) of methane, ethane, and propane is 25, 5.5, and 3, which is much higher than the GWP of CO_2_. Catalytic combustion or catalytic total oxidation technique has been considered to be a promising strategy for eliminating these emitted LHs. Moreover, due to their stable structure and high C‐H bond energies, catalytic combustion of LHs was generally also investigated as a model reaction of catalytic combustion of volatile organic compounds (VOCs) to design and develop efficient catalysts.^[^ ^2^, ^3^, ^4^ ^]^

The most promising Pd, Ru, and Co‐based catalysts presented an excellent overall performance for catalytic combustion of LHs and were currently the most attractive catalytic materials,^[^ ^5^, ^6^, ^7^ ^]^ particularly, Co_3_O_4_ and RuO_x_‐based catalysts were focused in the recent years due to their high activity and low cost. Spinel Co_3_O_4_ possessed numerous unique physicochemical properties, such as abundant unfilled d6/d7 orbitals, weak strength of Co‐O bonds, low formation energy of oxygen vacancies, and high mobility of lattice oxygen, hence, exhibited a high activity for the activation of the C‐H bonds through the direct interaction of the d orbitals with the σ/σ* C‐H orbitals, which was considered to contribute greatly to catalytic combustion of LHs.^[^ ^8^, ^9^ ^]^ Wang et al. suggested that the calcined Co_3_O_4_ at low temperatures such as 200 °C demonstrated the best catalytic activity of propane combustion (the temperature of 90% conversion, T_90_ = 170 °C) due to the excellent reducibility, abundant oxygen vacancies, and large surface area, however, which suffered from a severe sintering deactivation at high temperature (for example, 450 °C) and the T_90_ increased to 220 °C.^[^ ^10^ ^]^ Cao et al. found that the doping of La led to the formation of LaCoO_3_ perovskites and improved the thermal stability of the Co_3_O_4_ structure. After aging at 750 °C for 100 h, the activity loss of La‐Co_3_O_4_ was simply less than that of the pristine Co_3_O_4_ and the ΔT_90_ dropped from 217 °C (Co_3_O_4_) to 138 °C (La‐Co_3_O_4_), but this thermal deactivation was still inevitable and very serious.^[^ ^11^ ^]^ The supported Co_3_O_4_ such as Co_3_O_4_/ZSM‐5 exhibited a better catalytic activity for propane combustion than bulk Co_3_O_4_ due to the high concentration of active Co^3+^ and lattice oxygen species, but the easy sintering of Co_3_O_4_ inevitably caused a declining in activity after the stability test at 500 °C for 40 h due to the deficiency of the strong anchoring or stabilizing between Co_3_O_4_ and ZSM‐5 support.^[^ ^12^ ^]^ By contrast, the loading of Co_3_O_4_ on the reducible supports such as CeO_2_ and CeO_2_‐ZrO_2_ presented good activity and stability for methane combustion, the conversion decreased by only 8% and 7% after running 65 h at 600 °C, which was attributed to the suppressed decomposition and sintering of Co_3_O_4_ owing to the high oxygen mobility/storage capacity and the strong metal oxide‐support interaction.^[^ ^13^ ^]^ Similar to Co_3_O_4_‐based catalysts, supported Ru‐based catalysts such as Ru/CeO_2_ or Ru/γ‐Al_2_O_3_ presented a high catalytic activity for propane combustion (for example, T_90_ = 150 °C on Ru/CeO_2_ and T_50_ = 175 °C on Ru/γ‐Al_2_O_3_).^[^ ^14^ ^]^ Unfortunately, the leaching and sintering of Ru would occur at high temperatures under an oxidizing atmosphere and the activity decreased significantly, even which was much worse than Co_3_O_4_‐based catalysts.^[^ ^15^ ^]^ The encapsulated RuO_2_ (ultrafine Ru nanoclusters in silica‐1 zeolite, Ru_1_@S‐1) showed excellent stability and slightly decreased activity after aging at 700 °C under a nitrogen atmosphere, which was ascribed to the confined effect of the shell structure. However, the stability of this catalyst in an oxidizing atmosphere (under the reaction condition) was not comprehensively examined.^[^ ^16^ ^]^ Catalytic combustion of propane over Ru or Pd supported on cobalt‐doped alumina nanosheets (Ru/CoANS and Pd/CoANS) demonstrated that Ru/CoANS presented a superior activity and water‐resistance but a low sintering‐resistance compared with Pd/CoANS.^[^ ^17^ ^]^ Despite the high activity of Ru and Co‐based catalysts for catalytic combustion of LHs, their thermal stability still needed to be enhanced, which was extremely important for the practical working conditions of catalysts.

Catalytic combustion of LHs was involved in different scenarios such as the purification of vehicle exhaust including gasoline or natural gas engines and dual‐fuel marine engines, the production of Zero Air, the portable detection of non‐methane hydrocarbons (NMHC), and the removal of industrial emissions such as the petroleum processing, manufacture of acrylic acid and F‐T synthesis,^[^ ^1^ ^]^ the desired catalysts should be more possessed the superior sintering‐resistance at high temperatures (>750 °C) and water‐resistance under high concentrations of water vapor (>10 vol. %) besides the high activity/selectivity. Herein, rare earth elements (REs) doped holey Co_3_O_4_ nanosheets were prepared by a simple methanol solvothermal method and followed by the loading of Ru (Ru/REs‐Co_3_O_4_), then catalytic combustion of mixed LHs was performed. The thermal stability of Ru/REs‐Co_3_O_4_ especially Ru/La‐Co_3_O_4_ was evaluated in detail, interestingly, an aging‐reactivation phenomenon (an enhanced activity after aging at 750 °C) was observed not a thermal deactivation, even Ru/La‐Co_3_O_4_ presented a negligible activity declining after a prolonged high‐temperature aging (aging for 200 h at 750 °C in an oxidizing atmosphere). A series of well‐designed experiments and characterizations suggested that high‐temperature aging induced a reconstruction of Ru/La‐Co_3_O_4_, generating a LaRuO_3_ perovskite phase through the reaction of RuO_x_ with the exsolved La from the lattice of Co_3_O_4_, served as a new active site for LHs combustion, not only stabilized Ru species and Co_3_O_4_ structure. Moreover, the stabilization of Ru could be achieved by the generation of a thermally stable LaRuO_3_ perovskite phase, preventing the ruthenium from the formation of its volatile oxides under an oxidative atmosphere.^[^ ^18^ ^]^ This work presented an innovative approach to designing highly thermostable catalysts for catalytic combustion of LHs, which contributed to the effective elimination of organic pollutant emissions and the stabilization of Ru species.

## 2 Results and Discussion

### 2.1 Catalytic Combustion of LHs

**Figure 1:**
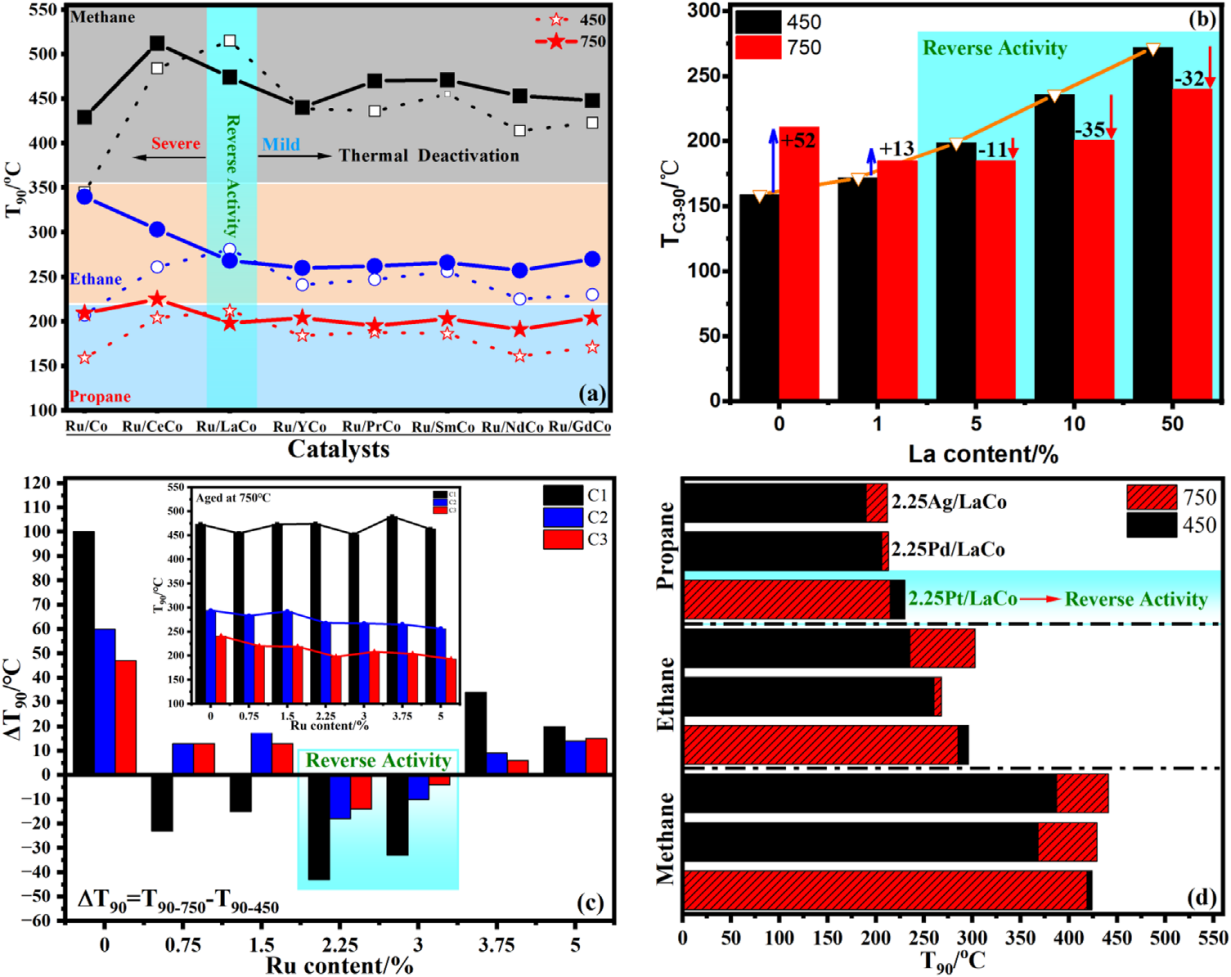
T_90_ of fresh and aged (at 750 °C) Ru/REs‐Co_3_O_4_ catalysts for catalytic combustion of mixed LHs a), the effect of La content on 2.25 wt% Ru/La‐Co_3_O_4_ b), effect of Ru content on Ru/5La‐Co_3_O_4_ c), and catalytic combustion of mixed LHs on fresh and aged (at 750 °C) precious metals supported 5La‐Co_3_O_4_ (precious metals = Ag, Pd, and Pt) d).

**Figure** **1**a displayed the T_90_ of fresh and aged (750 °C) Ru/Co_3_O_4_ for catalytic combustion of mixed LHs and the different rare earths doping was specifically compared, and the corresponding light‐off curves were also supplementarily presented in Figure S1 (Supporting Information). The doping of REs into Co_3_O_4_ did not increase the activity of Ru/Co_3_O_4_ as expected, instead an inhibition was observed for catalytic combustion of methane (C1), ethane (C2), or propane (C3). For example, the T_C3_‐_90_ (the temperature of 90% propane conversion) increased differently from 159 °C of Ru/Co_3_O_4_ to 161 °C (Ru/Nd‐Co_3_O_4_) and 227 °C (Ru/La‐Co_3_O_4_). But importantly, the aged Ru/REs‐Co_3_O_4_ presented a better activity than the aged Ru/Co_3_O_4_ although the thermal deactivation still occurred, the ΔT_90_ (T_90‐750_ ‐T_90‐450_) of propane combustion reduced from 50 to ≈20 °C, which meant the doping of REs enhanced the high‐temperature resistance of Ru/Co_3_O_4_.^[^ ^11^ ^]^ What was more, the doping of La brought a reverse activity, that is to say, the aged Ru/La‐Co_3_O_4_ demonstrated an enhanced activity. Taking propane combustion as an example, the ΔT_C3_‐_90_ declined by 11 °C. To further understand this phenomenon, the effect of La and Ru content was investigated and the T_90_ (T_C3‐90_ as a representative) and ΔT_90_ were shown in Figure 1b,c, more detailed data was provided in Figures S2 and S3 (Supporting Information). Even after a small amount of La (1 wt%) was doped, the high‐temperature resistance of Ru/Co_3_O_4_ was improved (ΔT_C3‐90_ only increased by 13 °C) but an enhanced activity was not observed. Increasing continuously to the higher La content (between 5 and 50 wt%), the aged catalysts began to show better activity and the enhancement of activity increased with the increasing of La content (ΔT_C3‐90_ varied from −11 to −35 °C). However, too much La would result in the activity decreasing due to the possible occupation of excess La on the active sites from Co_3_O_4_,^[^ ^19^ ^]^ subsequently, 5 wt% La as the optimal content was adopted. Figure 1c and Figure S3 (Supporting Information) suggested that the reverse activity also depended obviously on the presence of Ru (lower than 3 wt%) but the continuous increase of Ru would lead to the weakening of the high‐temperature resistance, the loading of 2.25 wt% Ru was considered to be the most suitable. Moreover, other noble metals such as Ag, Pt, and Pd were also supported on La‐Co_3_O_4_, but the reverse activity was determined on only the aged Pt/La‐Co_3_O_4_ and the enhanced activity was lower than Ru/La‐Co_3_O_4_. Although the supported Ag and Pd were slightly deactivated after aging at high‐temperature, the deactivation was partially suppressed compared with La‐Co_3_O_4_ (Figure 1d; Figure S4, Supporting Information). Summarily, the co‐presence of La and Ru improved greatly the high‐temperature resistance of Co_3_O_4,_ and even the aged catalysts with appropriate Ru and La content (for example, 2.25 wt% Ru and 5 wt% La) presented a better activity than the fresh catalysts, which possibly contributed to the increased thermal stability of Co_3_O_4_ owing to the introduction of La and Ru, but it was speculated that some other factors, such as the formation of new active phases and more defects (due to the exsolving of La from Co_3_O_4_ lattice), were additionally responsible for this reverse activity.

**Figure 2:**
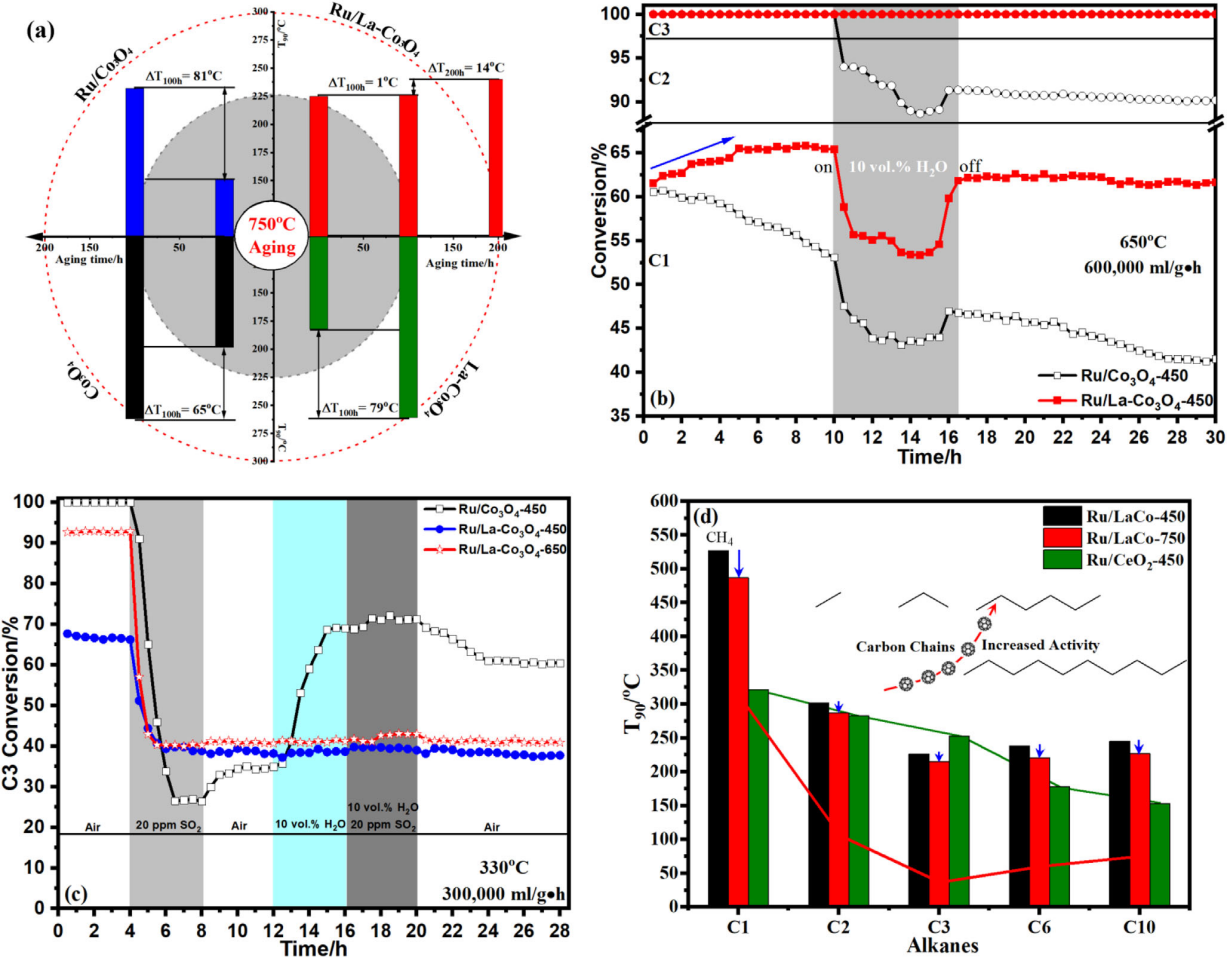
Catalytic combustion of LHs (ΔT_C3_‐_90_) on the aged Co_3_O_4_‐based catalysts at 750 °C for 100 h or 200 h (a), Long‐term stability test of Ru/Co_3_O_4_‐450 and Ru/La‐Co_3_O_4_‐450 at 650 °C and 6 00 000 mL g^−1^·h in the absence and presence of 10.0 vol.% H_2_O (b), Effects of H_2_O (10.0 vol.%) and SO_2_ (20 ppm) on the stability of Ru/Co_3_O_4_‐450, Ru/La‐Co_3_O_4_‐450, and Ru/La‐Co_3_O_4_‐650 at 330 °C and 300000 mL g^−1^·h (c), and catalytic combustion of alkanes with different carbon chain length on Ru/La‐Co_3_O_4_‐450, Ru/La‐Co_3_O_4_‐750 and Ru/CeO_2_‐450 catalysts (d).

To rapidly evaluate the life of catalysts and further verify the high‐temperature durability, **Figure** **2**a displayed the T_C3‐90_ of four Co_3_O_4_‐based catalysts after prolonged aging at 750 °C in an air atmosphere, and more details were shown in Figure S5 (Supporting Information). After 100 h, catalytic activities of Co_3_O_4_, La‐Co_3_O_4,_ and Ru/Co_3_O_4_ catalysts declined sharply, and the T_C3‐90_ increased by 65, 79, and 81 °C compared with fresh catalysts, respectively. Gratifyingly, the deactivation of Ru/La‐Co_3_O_4_ was almost negligible and the T_C3‐90_ increased by 1 °C, the T_C3‐90_ only increased by 15 °C even after aging for 200 h at 750 °C. Meanwhile, the activation energy of the catalysts was calculated after aging, and the results are presented in Figure S6 (Supporting Information). The data demonstrate that Ru/La‐Co_3_O_4_ exhibits the lowest activation energy, confirming that it retains excellent catalytic activity even after prolonged high‐temperature aging. Certainly, Ru/La‐Co_3_O_4_ exhibited excellent thermal stability, which was expected to be competent in the high‐temperature scenario of LHs combustion such as the removal of industrial emission from the manufacture of acrylic acid that is usually maintained at the high operating temperature (for example, 550–650 °C). Additionally, the stability of these catalysts was further evaluated under different reaction conditions, such as low temperature, high WHSV, high H_2_O concentration, or high temperature. Figure S7 (Supporting Information) displayed prolonged stability tests of Ru/Co_3_O_4_‐450, Ru/La‐Co_3_O_4_‐450, and Ru/La‐Co_3_O_4_‐750 at 250 °C and 300000 mL g^−1^·h in the absence and presence of 10.0 or 5.0 vol.% H_2_O under kinetically controlled conditions.^[^ ^20^ ^]^ Three catalysts presented a stable conversion of propane within 45 h (≈36% for Ru/Co_3_O_4_‐450, 20% for Ru/La‐Co_3_O_4_‐450 and Ru/La‐Co_3_O_4_‐750), and the introduction of H_2_O only caused a reversible deactivation. The reaction temperature increased to 330 °C, both Ru/Co_3_O_4_‐450 and Ru/La‐Co_3_O_4_‐750 still presented a super high and stable activity for catalytic combustion of mixed LHs (Figure S8, Supporting Information), while a slight deactivation occurred on Ru/La‐Co_3_O_4_‐450 with the lowest activity. These stability tests indicated that all three catalysts were of good stability at low temperatures, how stable were they at high temperatures? Therefore, more real and harsh conditions, 10.0 vol.% H_2_O, 600000 mL g^−1^h, and 650 °C, were used to evaluate the practicability of Ru/La‐Co_3_O_4,_ and the results were presented in Figure 2b. Within the initial 10 h (dry condition), the 100% conversion of ethane and propane was detected (61% methane conversion) and remained stable on both Ru/Co_3_O_4_ and Ru/La‐Co_3_O_4_. More importantly, Ru/Co_3_O_4_ and Ru/La‐Co_3_O_4_ exhibited different catalytic behaviors with the increasing reaction time, the former presented a declining conversion of methane while an induction period was observed on the latter within the initial 5 h and eventually stable. The sintering of Ru/Co_3_O_4_ was responsible for its deactivation, the better sintering‐resistance and possible structure reconstruction at high temperatures contributed to the enhanced activity of Ru/La‐Co_3_O_4_, which was consistent with the results from the aging experiments in the air atmosphere. After 10 vol.% H_2_O was introduced, the catalytic activity of both catalysts was inhibited but the 100% conversion of ethane and propane over Ru/La‐Co_3_O_4_ was still maintained, while the conversion of ethane decreased from 100% to 88% on Ru/Co_3_O_4_. When H_2_O was turned off, both catalysts were partially restored to the initial catalytic activity, meaning the presence of water did not cause irreversible deactivation. In the following 15 h, Ru/Co_3_O_4_ presented an ever‐descending performance due to the sintering of Co_3_O_4_ and Ru leaching, while Ru/La‐Co_3_O_4_ recovered to the initial activity and remained stable despite the loss of the enhanced activity from the induction period. Effects of SO_2_ or/and H_2_O also were preliminarily investigated (Figure S9, Supporting Information) and the conversion curves of propane as a representative were individually shown in Figure 2c, unfortunately, an irreversible and rapid deactivation was observed on all catalysts and even the introduction of H_2_O could not fully restore their activity, especially for Ru/La‐Co_3_O_4_. Catalytic combustion of alkanes with different carbon chain lengths such as n‐hexane (C6) and n‐decane (C10) including C1‐C3 on Ru/La‐Co_3_O_4_ was evaluated expansively and compared with Ru/CeO_2_ (the preparation could be obtained in Supporting Information) as a reference that presented the best activity for catalytic combustion of propane (Figure 2d; Figure S10, Supporting Information). With the increase of carbon chain, alkanes were more easily oxidized as expected due to the reduction of the C─H bonds energies, moreover, the aged Ru/La‐Co_3_O_4_ at 750 °C displayed a reverse activity for all alkanes and its activity was closed to Ru/CeO_2_.

### 2.2 Structural and Physicochemical Properties

**Figure 3:**
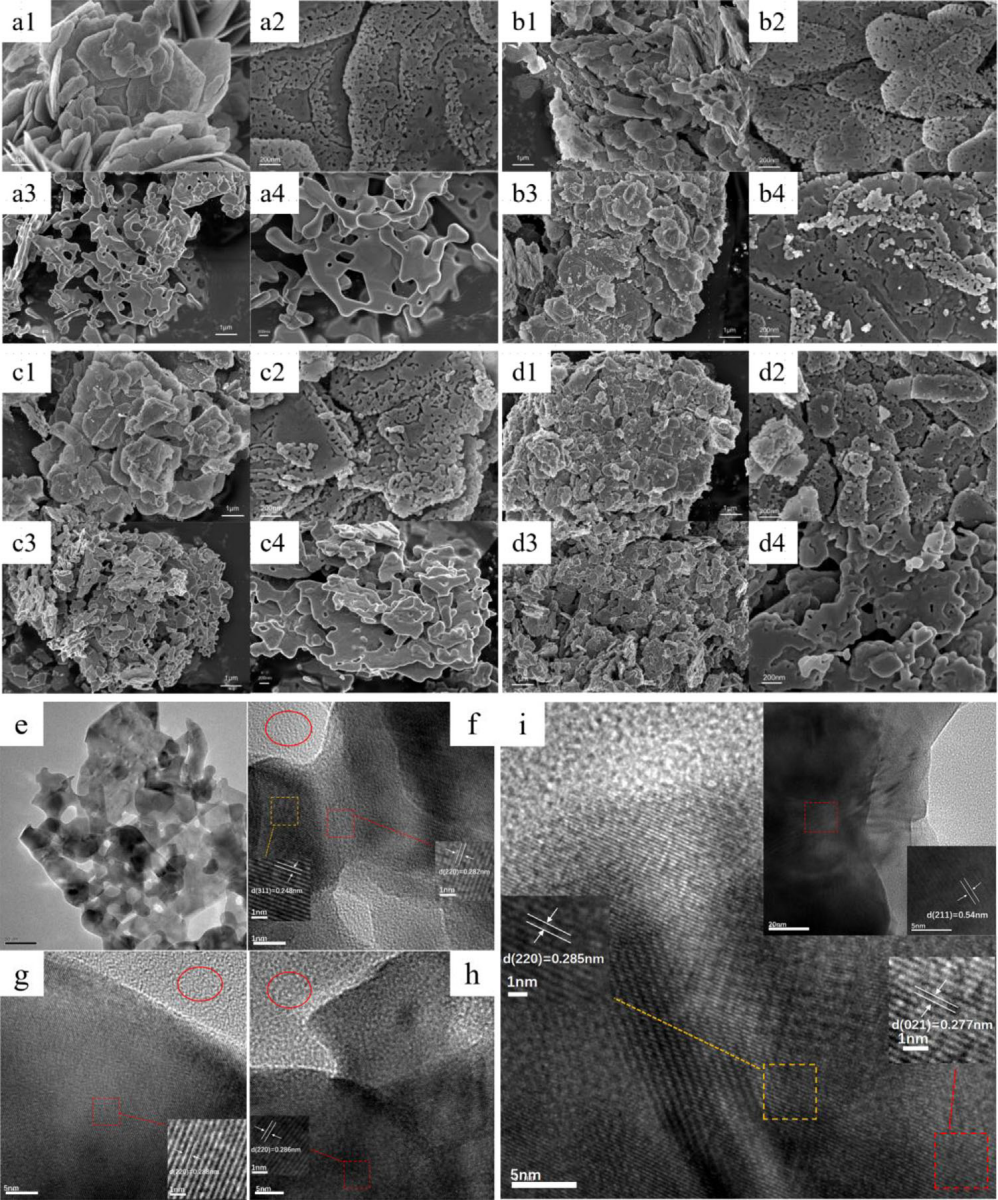
FLSEM images of fresh and aged Co_3_O_4_ (a1, a2 and a3, a4), La‐Co_3_O_4_ (b1, b2 and b3, b4), Ru/Co_3_O_4_ (c1, c2 and c3, c4) and Ru/La‐Co_3_O_4_ (d1, d2 and d3, d4), HRTEM images of Co_3_O_4_‐450 (e), Ru/Co_3_O_4_‐450 (f), Ru/Co_3_O_4_‐750 (g), Ru/La‐Co_3_O_4_‐450 (h) and Ru/La‐Co_3_O_4_‐750 (i).

FLSEM images shown in **Figure** **3**a1–d4 indicated that the prepared Co‐based catalysts and even the aged samples at 750 °C revealed a holey‐engineered 2D nanosheets architecture due to the rapid decomposition of plate‐like Co(OH)(OCH_3_) precursor and the formed organics.^[^ ^21^ ^]^ The doping of La and the high‐temperature calcination did not destroy this porous nanosheet structure. Importantly, the holes of the pristine Co_3_O_4_ (Figure 3a3,a4) and Ru/Co_3_O_4_ (Figure 3c3,c4) almost disappeared and a severe sintered aggregation occurred after aging at 750 °C, while the presence of La distinctly suppressed this sintering (Figure 3b3,b4 for La‐Co_3_O_4_, Figure 3d3,d4 for Ru/La‐Co_3_O_4_). In addition, the change of SSA (Table S2, Supporting Information) indicated that the pure Co_3_O_4_ catalyst experienced a severe reduction in SSA (74%) after aging at 750 °C for 4 h, consistent with significant sintering and structural collapse. In contrast, the Ru/La‐Co_3_O_4_ catalyst exhibits a much smaller reduction in SSA (50%), indicating that the co‐modification with Ru and La effectively mitigates sintering and preserves the catalyst's structural integrity. HRTEM image of the fresh Co_3_O_4_ (Figure 3e) further confirmed the porous sheet‐like structures. The magnified images revealed that only lattice fringes belonging to Co_3_O_4_ were determined and the lattice distances of 0.248 and 0.284 nm were assigned to the (311) and (220) facets of Co_3_O_4_ on Ru/Co_3_O_4_‐450 (Figure 3f), Ru/Co_3_O_4_‐750 (Figure 3g) and Ru/La‐Co_3_O_4_‐450 (Figure 3h).^[^ ^1^, ^22^ ^]^ For the Ru/La‐Co_3_O_4_‐750, the lattice distances of 0.540 and 0.277 nm corresponding to the (211) and (332) facets of LaRuO_3_ was additionally detected (Figure 3i),^[^ ^23^ ^]^ which suggested that a structure reconstruction of Ru/La‐Co_3_O_4_ occurred at the high temperature and a thermally stable LaRuO_3_ phase formed. Additionally, it is noteworthy that no lattice fringes or particles belonging to Ru or RuO_x_ were observed, indicating that Ru species were highly dispersed on the Co_3_O_4_ surface or doped into its lattice.^[^ ^1^ ^]^ Moreover, ICP results showed the content of Ru in Ru/La‐Co_3_O_4_‐450, Ru/La‐Co_3_O_4_‐750 and Ru/Co_3_O_4_‐750 was 2.24 wt%, 2.17 wt% and 1.41 wt%, the thermal leaching of Ru was observed over Ru/Co_3_O_4_ while not over Ru/La‐Co_3_O_4_, which was ascribed to the stabilization of LaRuO_3_.

**Figure 4:**
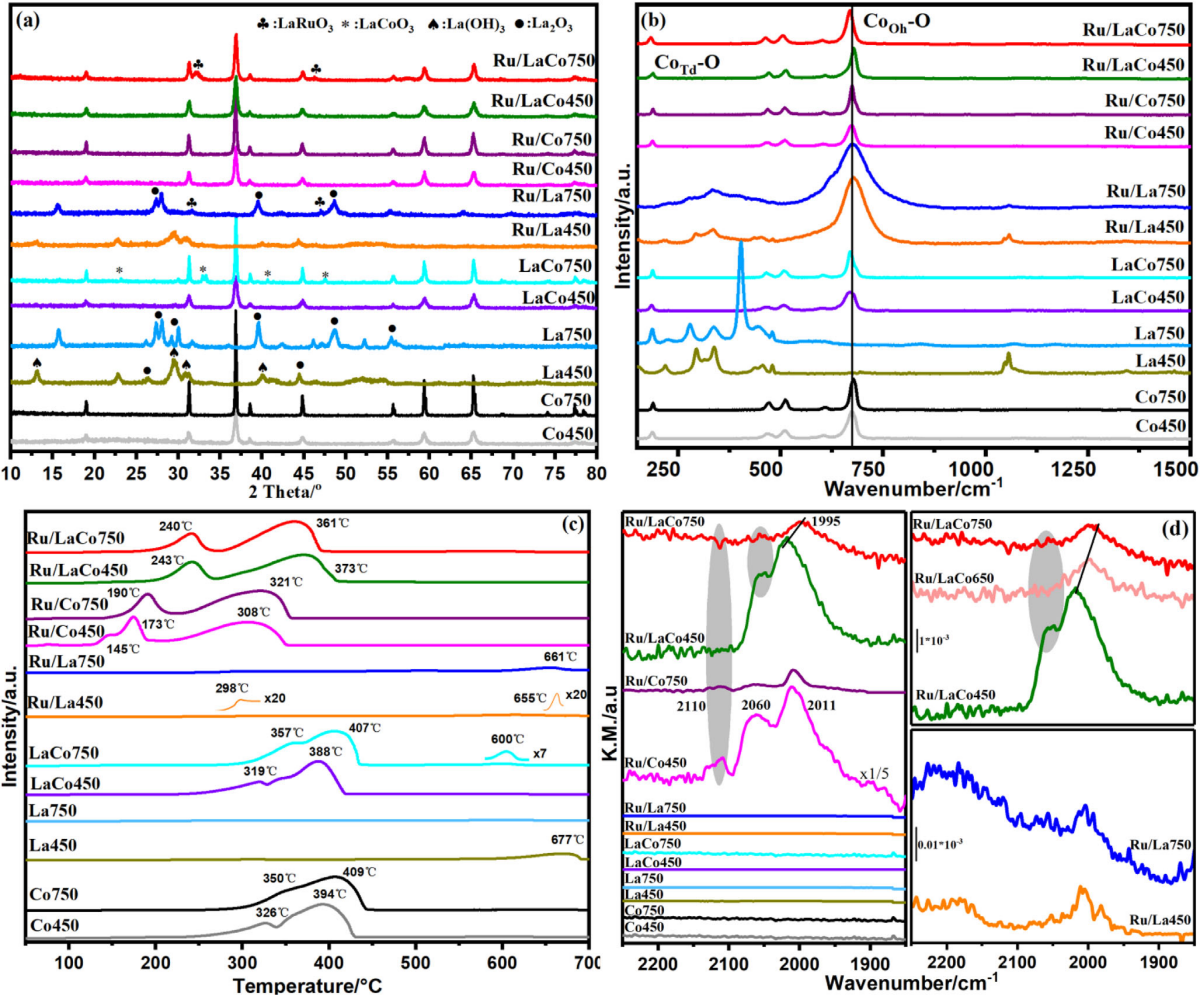
XRD patterns (a), Raman spectra (b), H_2_‐TPR (c), and CO‐DRIFTS (d) of fresh and aged Co_3_O_4_, La_2_O_3_, La‐Co_3_O_4_, Ru/La_2_O_3_, Ru/Co_3_O_4,_ and Ru/La‐Co_3_O_4_ catalysts.

**Figure 5:**
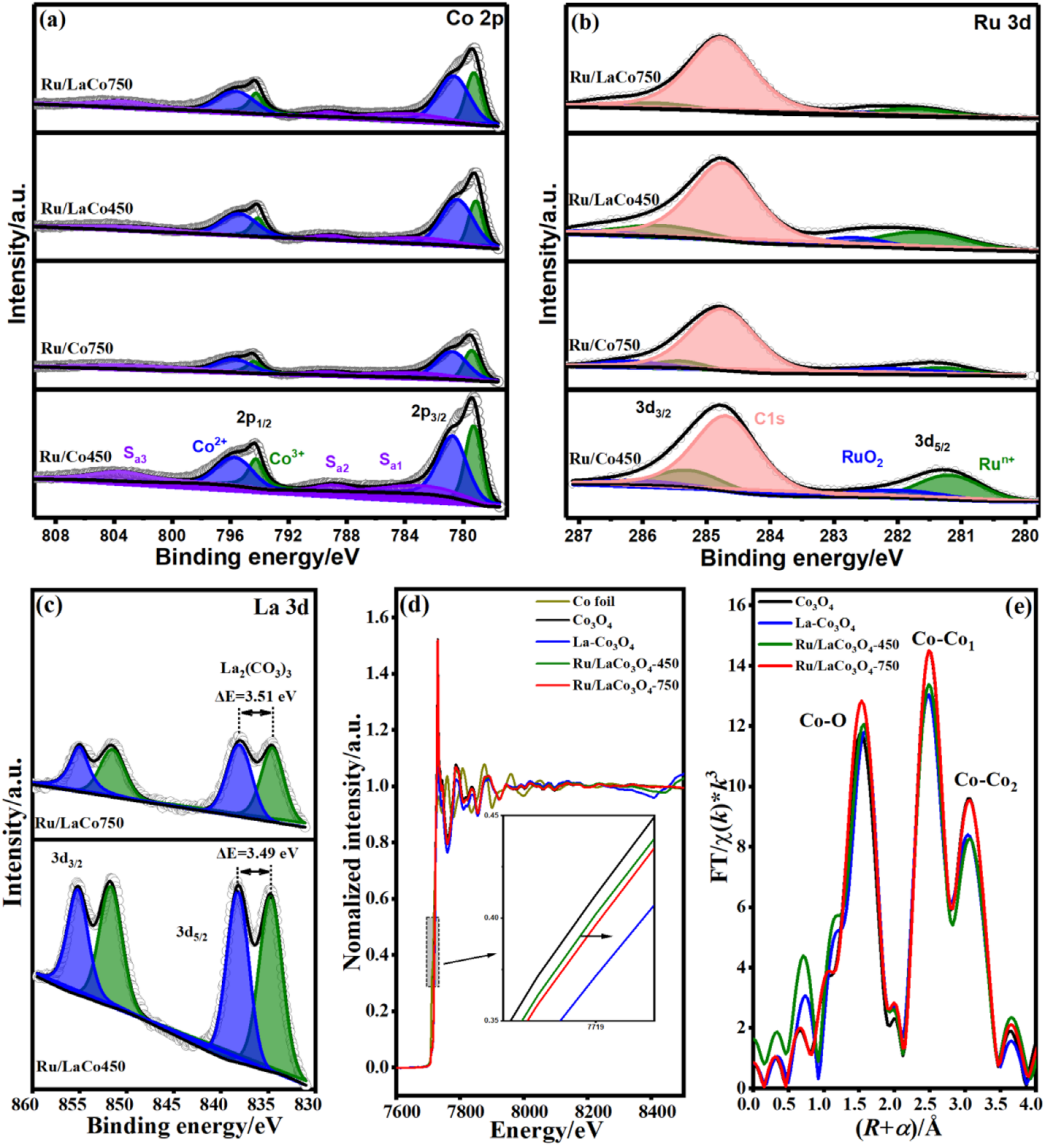
Co 2p (a) Ru 3d (b), La 3d (c) XPS spectra of fresh and aged La‐Co_3_O_4_‐450, Ru/Co_3_O_4_ and Ru/La‐Co_3_O_4_ catalysts, Co K‐edge XANES spectra (d) and Fourier transform of *k* ^3^‐weighted EXAFS profiles (e) of Co_3_O_4_‐450, La‐Co_3_O_4_‐450, Ru/La‐Co_3_O_4_‐450 and Ru/La‐Co_3_O_4_‐750.

The crystal structure, redox ability, and chemical state of Ru of the prepared catalysts further were analyzed by XRD, Raman, H_2_‐TPR, CO‐DRIFTS, XPS, and EXAFS, and shown in **Figures** **4** and **5**. XRD patterns (Figure 4a) of all cobalt‐based catalysts displayed characteristic diffraction peaks of cubic spinel Co_3_O_4_ (PDF #43‐1003). La phases including La_2_O_3_, La(OH)_3,_ and La_2_(CO_3_)_3_ were not observed for the La‐doped Co_3_O_4_ due to the high dispersion or lattice doping of La, moreover, no diffraction peaks belonging to RuO_x_ phases were observed on all supported RuO_x_ catalysts. La_2_O_3_‐450 showed a series of diffraction peaks corresponding to the mixed crystalline phase of La_2_O_3_ (PDF #05‐0602) and La(OH)_3_ (PDF #36‐1481) but without La_2_(CO_3_)_3_ (PDF #25‐1400), while the highly crystalline hexagonal La_2_O_3_ was formed after aging at 750 °C.^[^ ^24^ ^]^ It was worth concerning that, for the aged samples at 750 °C, the LaCoO_3_ perovskite phase appeared on La‐Co_3_O_4_‐750,^[^ ^25^ ^]^ but the loading of Ru suppressed the formation of LaCoO_3_ and a new LaRuO_3_ perovskite phase was observed on Ru/La‐Co_3_O_4_‐750 (confirmed by HRTEM results and XRD pattern of Ru/La_2_O_3_‐750).^[^ ^18^, ^26^ ^]^ Furthermore, Figure S12 (Supporting Information) showed low‐speed scanning (2°/min) XRD analysis, and the new pattern revealed LaRuO_3_ diffraction peaks with significantly enhanced intensity and additional peaks, providing clear evidence of the formation of LaRuO_3_ perovskite on the Ru/La‐Co_3_O_4_ catalyst. The enlarged XRD patterns shown in Figure S11 (Supporting Information) indicated that the diffraction peak of Ru/Co_3_O_4_ was shifted to a lower angle after aging at 750 °C, suggesting that Ru^4+/3+^ (62/68 pm ionic radius) doped into the lattice of Co_3_O_4_ and substituted the part octahedral Co^3+^ (63 pm).^[^ ^21^ ^]^ In contrast, the diffraction peaks of Ru/La‐Co_3_O_4_ shifted toward a high angle after aging at 750 °C, probably due to the exsolving of La^3+^ (106 pm) from the lattice of Co_3_O_4_ to form LaRuO_3_ perovskite reacting with RuO_x_. Additionally, the high‐temperature aging caused the the sintering of Co_3_O_4_ based on the peak intensity, but the introduction of La and Ru significantly enhanced its sintering‐resistance and coincided with FLSEM results. Raman spectra (Figure 4b) verified the Co_3_O_4_ spinel structures of cobalt‐based catalysts, in practice, the two main bands at ≈676 and 186 cm^−1^ corresponding to the A_1_ _g_ symmetry of octahedral site (CoO_6_) and the F_2g_^[^ ^1^ ^]^ symmetry of tetrahedral site (CoO_4_) were observed besides the bands at ≈470, 512, and 606 cm^−1^.^[^ ^27^ ^]^ The doping of La shifted these bands especially octahedral sites to a low wavenumber, which was an indication of the CoO_6_ lattice doping and the distortion of Co─O bonds.^[^ ^25^ ^]^ More importantly, this shift was highly dependent on the aging temperature for Ru/La‐Co_3_O_4_, a more significant redshift was observed on the aged Ru/La‐Co_3_O_4_ at high calcination temperatures such as 750 °C even 650 °C (Figure S13, Supporting Information) and a severe distortion of Co_3_O_4_ spinel structure occurred, which was ascribed to the exsolving of La from the Co_3_O_4_ lattice to form LaRuO_3_ perovskite, and subsequently generated more cation vacancies. Generally, more distorted Co─O bonds and more cation vacancies were considered to facilitate the generation of more reactive oxygen species and oxygen capacity, which could be attributed to the catalytic combustion of LHs.^[^ ^27^ ^]^ H_2_‐TPR indicated that cobalt‐based catalysts presented two characteristic reduction peaks (Figure 4c), corresponding to the stepwise reduction of Co^3+^ to Co^2+^ and Co^2+^ to Co,^[^ ^1^ ^]^ but an additional reduction peak at 600 °C assigning to the reduction of LaCoO_3_ was observed on La‐Co_3_O_4_‐750.^[^ ^25^ ^]^ The doping of La and the loading of Ru both promoted the redox ability of Co_3_O_4_ due to the distortion of Co‐O bonds, especially the loading of Ru drastically decreased the reduction temperature by ≈80–150 °C due to the oxygen spillover between precious metal and metal oxide.^[^ ^28^ ^]^ Conversely, the high‐temperature aging caused a shift to the high temperature owing to the sintering.^[^ ^1^ ^]^ However, Ru/La‐Co_3_O_4_‐750 displayed an enhanced redox performance and the reduction peaks decreased by 12 °C compared with Ru/La‐Co_3_O_4_‐450 (a similar phenomenon was also observed on Ru/La‐Co_3_O_4_‐650, Figure S14, Supporting Information), which was ascribed to the formation of LaRuO_3_ perovskite and the further distortion of Co_3_O_4_ structure. For Ru/Co_3_O_4_‐450, an additional peak at 145 °C appeared and was considered to be the reduction of highly dispersed RuO_x_.^[^ ^1^ ^]^ By the way, La_2_O_3_‐based catalysts showed a reduction peak of carbonate species between 650 °C and 680 °C, and a reduction peak of RuO_x_ at 298 °C was found on Ru/La_2_O_3_‐450 but disappeared after aging at 750 °C.^[^ ^25^ ^]^ in situ DRIFTS of CO chemisorption at 30 °C (Figure 4d) showed that CO adsorption bands were detected only on supported RuO_x_ catalysts and its intensity descended obviously after aging at 750 °C, which possibly because the sintering of Ru and the strong metal‐support interaction occurred.^[^ ^1^ ^]^ Specifically, three major bands appeared on Ru/Co_3_O_4_, the bands at 2060 and 2110 cm^−1^ were ascribed to the adsorption of multi‐carbonyl on oxidized Ru sites as ([Ru^n+^‐(CO)_x_]),^[^ ^29^ ^]^ and the bands at 2011 cm^−1^ could be attributed to dicarbonyl species adsorbed on low‐oxidation‐state Ru sites or reduced Ru.^[^ ^30^ ^]^ When doped with La (Ru/La‐Co_3_O_4_‐450 and Ru/La‐Co_3_O_4_‐750), the weak band at 2110 cm^−1^ disappeared and the high‐oxidation‐state Ru partially reduced due to the electron donation from La.^[^ ^25^ ^]^ More importantly, on Ru/La‐Co_3_O_4_‐750, the peak at 2060 cm^−1^ also disappeared and the band at 2011 cm^−1^ was redshifted to 1995 cm^−1^ (Ru/La‐Co_3_O_4_‐650 confirmed this result), which indicated that low‐oxidation‐state Ru produced and coincided with the formation of LaRuO_3_ perovskite. Additionally, a very weak CO chemisorption was observed on Ru/La_2_O_3_, indicating that Ru species were poorly dispersed on La_2_O_3_.

Co 2p XPS spectra depicted in **Figure** **5**a displayed two main spin‐orbit doublets peaks (besides three satellite peaks) in the range of 779.1–780.1 eV with a 2p_3/2_‐2p_1/2_ splitting of 15.0 eV and 780.3–781.4 eV with a 2p_3/2_‐2p_1/2_ splitting of 15.0 eV, which was characteristic of octahedral Co^3+^ and tetrahedral Co^2+^.^[^ ^21^ ^]^ The peaks of Ru/La‐Co_3_O_4_‐450 were slightly shifted to a low binding energy compared with Ru/Co_3_O_4_‐450 due to the electron donation of La to Co.^[^ ^25^ ^]^ A shift to higher binding energy was found after the high‐temperature aging, and the calculated ratio of Co^3+^/Co^2+^ (Table S3, Supporting Information) revealed that the Co^3+^ content of Ru/La‐Co_3_O_4_‐750 increased. XANES (Figure 5d) and EXAFS (Figure 5e) were employed to further confirm the chemical state of Co species. Ru/La‐Co_3_O_4_‐750 presented a higher oxidation state of Co species than Ru/La‐Co_3_O_4_‐450,^[^ ^31^ ^]^ and the largest number of Co─O coordination and coordinated oxygen (Table S4, Supporting Information),^[^ ^32^ ^]^ which was ascribed to the leaching of La from the Co_3_O_4_ lattice and generated more cation vacancies, facilitating the generation of more oxygen species. Additionally, according to Ru 3d XPS spectra shown in Figure 5b, Ru^n+^ (0<n<4) and Ru^4+^ species could be identified by two characteristic peaks in the range of 281.1–281.7 eV with a 3d_5/2_‐3d_3/2_ splitting of 4.1 eV and 282.0–282.9 eV with a 3d_5/2_‐3d_3/2_ splitting of 4.1 eV,^[^ ^33^ ^]^ the peak intensity indicated that the surface Ru content of Ru/Co_3_O_4_ was higher than Ru/La‐Co_3_O_4_ due to the inhibition of La on the dispersion of Ru (in situ DRIFTS of CO chemisorption and H_2_‐TPR results). After aging at 750 °C, the ratio of Ru^4+^/Ru^n+^ (Table S3, Supporting Information) in Ru/Co_3_O_4_ increased and mainly ascribed to the dramatic decrease of Ru^n+^ species (significantly reduced peak area), which also revealed that Ru^n+^ species was dominant in the highly dispersed and easily sintered RuO_x_, while the formation of LaRuO_3_ perovskite was responsible for the decreased ratio of Ru^4+^/Ru^n+^ in Ru/La‐Co_3_O_4_. XPS spectra of La 3d (Figure 5c) suggested that surface La species existed on Ru/La‐Co_3_O_4_ in the form of La_2_(CO_3_)_3_ based on the ≈3.50 eV ΔE (ΔE is difference of split peaks), which was different from the bulk La species and contributed to the formation of LaRuO_3_ perovskite.^[^ ^34^ ^]^

### 2.3 Determination of Structure‐Activity Relationship

**Figure 6:**
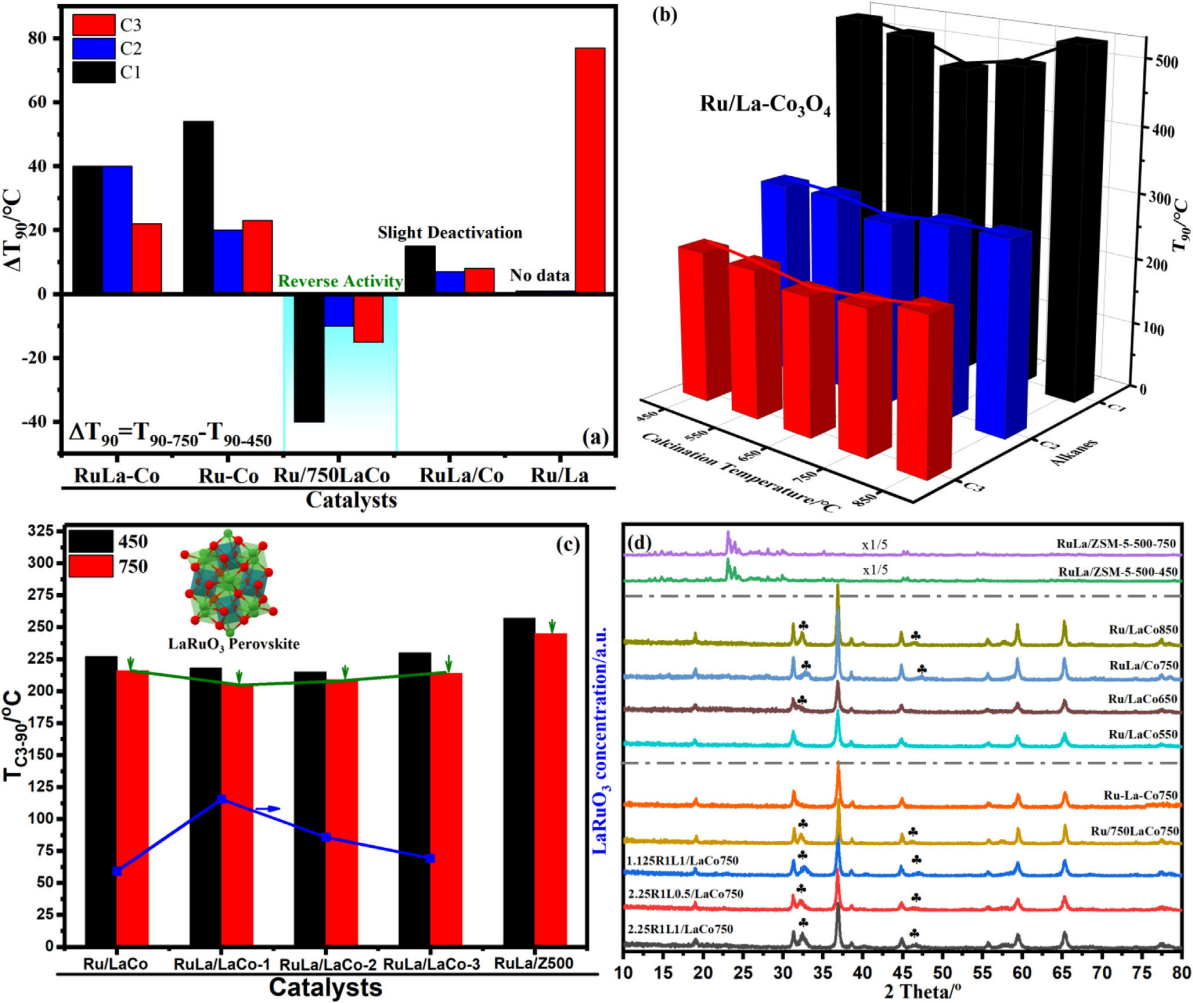
ΔT_90_ of fresh and aged RuLa‐Co_3_O_4_, Ru‐Co_3_O_4_, Ru/750La‐Co_3_O_4_ (La‐Co_3_O_4_ support was calcined at 750 °C), RuLa/Co_3_O_4_ and Ru/La_2_O_3_ catalysts for catalytic combustion of LHs (a), T_90_ of fresh and aged Ru/La‐Co_3_O_4_ at different temperatures (b), T_90_ of fresh and aged Ru/La‐Co_3_O_4_, RuLa/La‐Co_3_O_4_ (5%La‐Co_3_O_4_ as support, the loading of Ru and La as follows: RuLa/LaCo‐1) 2.25 wt% Ru and the mole ratio of La/Ru is 1, RuLa/LaCo‐2) 2.25 wt% Ru and the mole ratio of La/Ru is 0.5, and RuLa/LaCo‐3) 1.125 wt% Ru and the mole ratio of La/Ru is 1), RuLa/ZSM‐5 (SiO_2_/Al_2_O_3_ = 500) (c), and XRD patterns of catalysts for controlled experiments (d).

HRTEM and XRD suggested that a new LaRuO_3_ perovskite phase could be formed by a thermally induced treatment such as the aging of Ru/La‐Co_3_O_4_ at 750 °C, meanwhile, the loading of Ru and the doping of La improved the resistance to sintering of Co_3_O_4_. Moreover, the high temperature aging brought the enhanced redox ability (H_2_‐TPR), the severe distortion of Co─O bonds (Raman), a higher oxidation state of Co and more coordinated oxygen (XPS and XAS), and the more low‐valent Ru (CO‐DRIFTS), which was considered to contribute to catalytic combustion of LHs. However, what were the roles of the formed LaRuO_3_ phase? A series of additional catalysts as controlled experiments were designed and their activity were presented in **Figure** **6**. For example, the prepared RuLa‐Co_3_O_4_ and Ru‐Co_3_O_4_ by the one‐pot solvothermal method (namely Ru and La or Ru doped Co_3_O_4_) obviously deactivated after aging at 750 °C and the ΔT_C3‐90_ was up to ≈25 °C (Figure 6a; Figure S15, Supporting Information), although their catalytic activity was comparable to the pristine Co_3_O_4_ and even better. Moreover, the deactivation still occurred on bimetallic Ru and La supported Co_3_O_4_ (RuLa/Co_3_O_4_) but was slightly less (ΔT_C3‐90_ was ≈15 °C), which was probably ascribed to the serve sintering of bulk Co_3_O_4_ at high temperature. Thus, Ru/750La‐Co_3_O_4_ (Ru supported on the calcined La‐Co_3_O_4_ at 750 °C) was prepared. Although Ru/750La‐Co_3_O_4_ presented a low activity compared with Ru/La‐Co_3_O_4_ due to the partial sintering of La‐Co_3_O_4_, an enhanced activity was observed after a second high‐temperature calcination (Ru/750La‐Co_3_O_4_‐750). Additionally, Ru/La_2_O_3_ was lowly active for catalytic combustion of LHs and the high‐temperature aging led to the most severe deactivation. Based on the previous discussion and XRD results shown in Figure 6d (LaRuO_3_ perovskite formed on Ru/750La‐Co_3_O_4_‐750 but not on RuLa‐Co_3_O_4_‐750), it could be deduced that the dispersed LaRuO_3_ perovskite on Co_3_O_4_ especially La‐Co_3_O_4_ with higher thermal stability was responsible for this reverse activity. Thus, the aged Ru/La‐Co_3_O_4_ at different temperatures was investigated, and the results showed that all the aged Ru/La‐Co_3_O_4_ at high temperatures presented an enhanced activity except for the aging at 850 °C with a very slight deactivation (Figure 6b; Figure S16, Supporting Information). Meanwhile, the LaRuO_3_ phase was clearly detected by XRD. Subsequently, the content of the LaRuO_3_ phase on La‐Co_3_O_4_ support was customized by adjusting the Ru content and the ratio of La and Ru (Figure 6d) and was proportional to the increasing level of activity (Figure 6c; Figure S17, Supporting Information). Additionally, Ru and La supported ZSM‐5 (SiO_2_/Al_2_O_3_ = 500, and the preparation could be obtained in SI) also revealed an enhanced activity after aging at 750 °C but the LaRuO_3_ phase was not observed in Figure S18 (Supporting Information) (highly dispersed due to the large surface area of ZSM‐5). Therefore, it can be clearly identified that the dispersed LaRuO_3_ perovskite phase also was highly active for the catalytic combustion of LHs especially compared with the dispersed RuO_x_. Thus, the aged Ru/La‐Co_3_O_4_ at high temperature presented an enhanced activity rather than a thermal deactivation, and super thermal stability for catalytic combustion of LHs due to the outstanding resistance to sintering of LaRuO_3_ perovskite and La‐doped Co_3_O_4_.

**Figure 7:**
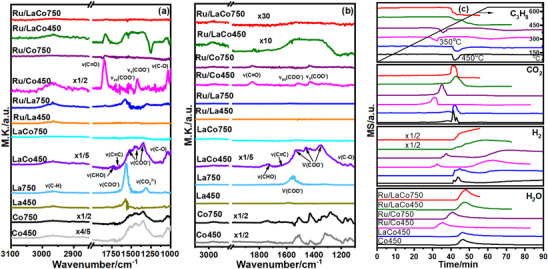
In situ DRIFTS spectra of propane adsorption (a) and oxidation (b) over fresh and aged Co_3_O_4_, La_2_O_3_, La‐Co_3_O_4_, Ru/La_2_O_3_, Ru/Co_3_O_4,_ and Ru/La‐Co_3_O_4_ catalysts at 250 °C, C_3_H_8_‐TPR profiles of Co_3_O_4_ based catalysts (c).

Catalytic combustion of propane as representative LHs was further investigated by in situ DRIFTS and C_3_H_8_‐TPR (namely temperature programmed oxidation of propane in the absence of oxygen, C_3_H_8_‐TPSR) to determine the intermediate distribution and understand possible reaction mechanism of LHs oxidation. As shown in **Figure** **7**a, in situ DRIFTS of propane adsorption at 250 °C in the absence of gaseous O_2_ displayed some weak bands assigned to C‐H bonds (CH_2_ and CH_3_) in the region 2800–3100 cm^−1^ and the adsorption of propane occurred over all catalysts.^[^ ^14^ ^]^ Furthermore, a series of bands at 1730, 1651, 1525, 1441, 1352, and 1000–1200 cm^−1^ were observed on Co_3_O_4_ and La‐Co_3_O_4_, which ascribed to aldehyde (*v*(C═O)), *v*(C═C), *v*_as_(COO^−^), versus(COO^−^), formate species and C─O bonds of alkoxide species,^[^ ^14^, ^35^ ^]^ respectively. However, after the introduction of Ru (including Ru/La_2_O_3_‐750, Ru/Co_3_O_4,_ and Ru/La‐Co_3_O_4_ catalysts), the characteristic bands assigned to the aldehyde group disappeared and a new characteristic band attributed to acetone *v*(C═O) at 1850 cm^−1^ appeared.^[^ ^36^ ^]^ Other bands ascribed to *v*_as_(COO^−^), versus(COO^−^), and *v*(C─O) at 1538, 1429, and 1000–1200 cm^−1^ were still preserved. Therefore, even in the absence of oxygen, the oxidation of propane could occur and the lattice oxygen of Co_3_O_4_ and RuO_x_ probably was directly involved.^[^ ^37^ ^]^ The possible reaction mechanism of propane oxidation was: propane adsorbed on the Co^3+/2+^ or oxygen vacancies, and the end‐group hydrogen was activated to generate propylene intermediates (called the propylene pathway) and then was oxidized to acrolein and acrylic species, until CO_2_ and H_2_O by active lattice oxygen. In the presence of RuO_x_ species, the adsorption of the isopropyl group on Ru/RuO_x_ occurred (called the isopropyl pathway),^14^ the corresponding acetone intermediate reacted with the active lattice oxygen species from Co_3_O_4_ or LaRuO_3_ to form formate and acetate species and finally oxidized to CO_2_ and H_2_O. After gaseous O_2_ was introduced (Figure 7b), similar characteristic bands were determined with that in the absence of O_2_, but the intensity of the bands became weaker and suggested that the oxidation of these intermediate accelerated in the presence of O_2_ due to the rapid replenishing of gaseous O_2_ into the lattice oxygen or the better complete‐oxidation ability of adsorbed surface oxygen species. C_3_H_8_‐TPR (Figure 7c) further revealed the oxidation of propane in the absence of O_2_ and the direct involvement of the lattice oxygen, the strong positive peaks (m/z = 44 and 18, CO_2_ and H_2_O) and negative peaks (m/z = 29, C_3_H_8_) appeared. Importantly, the supported RuO_x_ greatly promoted the propane oxidation and the peak temperature decreased from 450 °C of pristine Co_3_O_4_ to 350 °C of Ru/Co_3_O_4_, which confirmed that the introduction of Ru accelerated migration of the lattice oxygen (consistent with H_2_‐TPR results). The high‐temperature aging suppressed the releasing of the lattice oxygen and propane oxidation, however, the negative effect was not observed on Ru/La‐Co_3_O_4_‐750 and even a promotion occurred, meaning that the highly migrated lattice oxygen generated and was related to the formed LaRuO_3_. Unexpectedly, the production of much more H_2_ was detected over Ru/La‐Co_3_O_4_ and even Co_3_O_4_ was completely reduced above 600 °C (C_3_H_8_‐TPR was forced to stop because the U‐shaped reactor was blocked by the formed metallic Ru and Co), the better dehydrogenation ability (the breaking of the C‐H bonds) was considered to benefit propane oxidation, it can even be inferred that Ru/La‐Co_3_O_4_ was also great potential for the dehydrogenation of LHs.

**Scheme 1:**
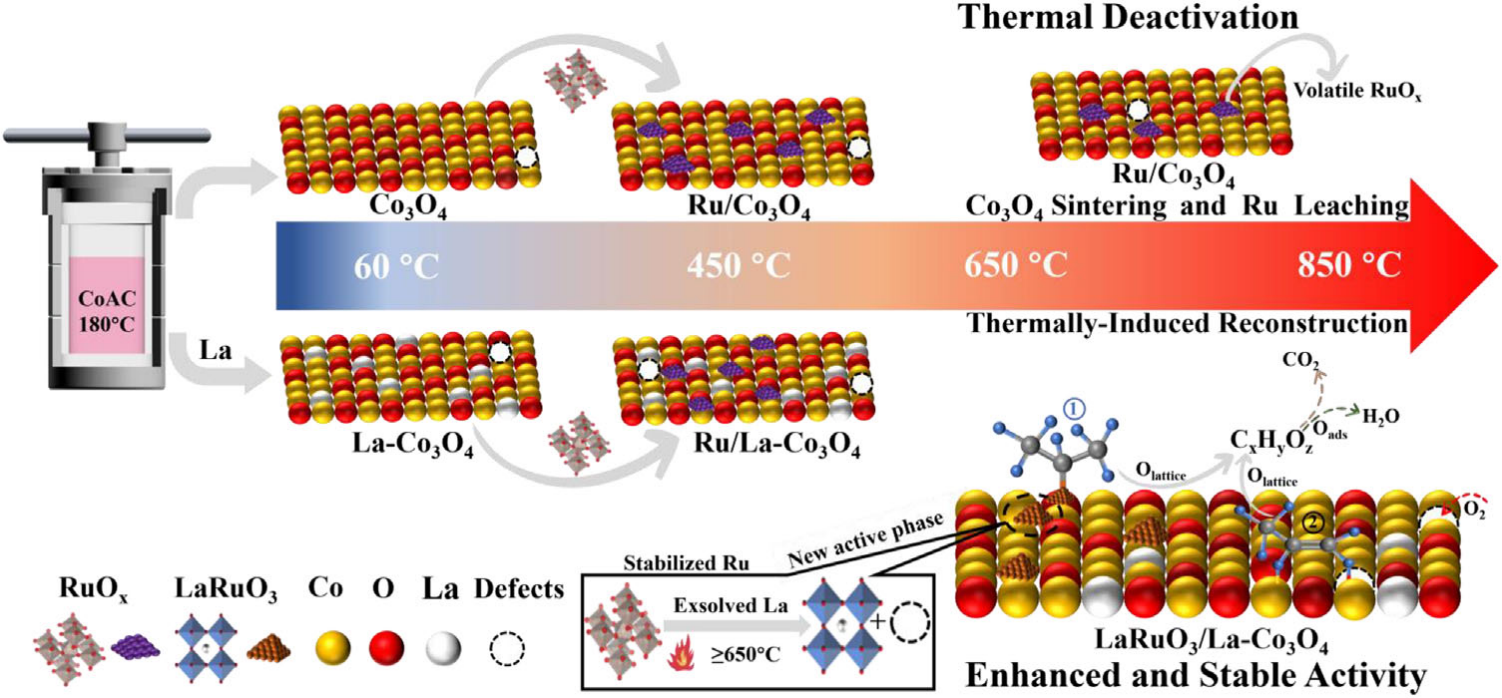
Structural evolution of Ru/Co_3_O_4_ and Ru/La‐Co_3_O_4_ catalysts under high temperature and proposed reaction pathways of propane oxidation over Ru/La‐Co_3_O_4_.

Summarily, the structural evolution of Ru/Co_3_O_4_ and Ru/La‐Co_3_O_4_ catalysts under high temperature and the proposed reaction pathways of propane oxidation as a representative of LHs oxidation over Ru/La‐Co_3_O_4_ were depicted in **Scheme** **1**. The doping of La boosted the resistance to sintering of Co_3_O_4_, importantly, the high‐temperature treatment (above 650 °C) induced the exsolving of La from the lattice of Co_3_O_4_ and then the formation of LaRuO_3_ perovskite (thermally‐induced reconstruction), which generated more vacancies and the distorted Co─O bonds of Co_3_O_4_ and brought the stabilization of Ru species in an oxidizing atmosphere. The dissociated adsorption of LHs such as propane occurred mainly on vacancies and the distorted Co─O of Co_3_O_4_ while LaRuO_3_ perovskite as a new active phase was also contributed over the high‐temperature aged Ru/La‐Co_3_O_4_, thus two reaction pathways called propylene and isopropyl pathways were observed. Then, the dissociated propane was stepwise oxidized by the surface lattice and adsorbed oxygen species.

## 3 Conclusion

In summary, we have successfully developed stable Co_3_O_4_ spinel catalysts by lattice doping with La and surface loading of RuO_x_, which presented a high activity for catalytic combustion of LHs, especially ethane, and propane. More importantly, the outstanding resistance to high temperature (750 °C) and H_2_O (10 vol.%) was demonstrated, the aged Ru/La‐Co_3_O_4_ at 750 °C presented a better activity than a fresh catalyst and only a slight activity decline was generated (T_90‐C3_ only increased by 15 °C) even after aging at 750 °C for 200 h. The studies of the structure‐activity relationship revealed that the co‐presence of La and Ru enhanced the high‐temperature stability of Co_3_O_4_ and promoted the migration of the lattice oxygen, the high‐temperature treatment induced the formation of LaRuO_3_ perovskite with a high activity through the reaction of RuO_x_ with the exsolved La from the lattice of Co_3_O_4_ (meanwhile more vacancies and the distorted Co‐O bonds of Co_3_O_4_ also generated), which were jointly responsible for catalytic combustion of LHs. This work provided a simple and feasible idea to design highly thermally stable Co_3_O_4_ spinel catalysts for catalytic combustion of LHs and a strategy for stabilizing the Ru species to inhibit the loss of RuO_x_ at high‐temperature in an oxidizing atmosphere, which contributed to the effective elimination of LHs and VOCs emissions and the stabilization of Ru species. Additionally, it is speculated that Ru/La‐Co_3_O_4_ also has great potential for catalytic oxidative dehydrogenation of LHs.

## 4 Experimental Section

### Materials

Cobalt acetate tetrahydrate and Lanthanum acetate tetrahydrate were purchased from Sinopharm Chemical Reagent Co., Ltd. (Shanghai, China). Ceriu acetate hexahydrate, Yttrium acetate, Praseodymium acetate pentahydrate, Samarium acetate pentahydrate, Neodymium acetate pentahydrate, and Gadolinium acetate trihydrate were purchased from Shanghai Macklin Biochemical Technology Co., Ltd. (Shanghai, China). Ruthenium nitrosyl nitrate, Platinum nitrate, silver nitrate, and palladium nitrate were purchased from Shanghai Praseodymium Strontium New Material Technology Co., Ltd. (Shanghai, China).

### Preparation of Holey Co_3_O_4_ Nanosheets

Holey Co_3_O_4_ nanosheets and REs doped Co_3_O_4_ nanosheets were prepared by a reported methanol solvothermal method with slight modifications.^[^ ^21^ ^]^ In a typical procedure: 3.54 g of cobalt acetate (Co(CH_3_COO)_2_·4H_2_O, CoAC) was dissolved in 75 mL methanol in a 100 ml Teflon autoclave and stirred for 15 min and then transferred to an oven at 180 °C for 24 h. After cooling to room temperature, the precipitate was filtered, washed using absolute alcohol, and then dried at 60 °C for 12 h. Finally, the dried precipitate was calcined in a muffle furnace at 450 °C for 4 h at a ramp rate of 5 °C min^−1^ to produce holey Co_3_O_4_ nanosheets. Ce, La, Y, Pr, Sm, Nd or Ga doped Co_3_O_4_ nanosheets (REs‐Co_3_O_4_) were prepared using the same steps with the addition of the corresponding acetate and the molar ratio of REs and Co was 5:95, the prepared catalysts were abbreviated as REsCo.

### Preparation of Supported RuO_x_ Catalysts

The loading of Ru was prepared by an incipient‐wetness impregnation method using Ru(NO)(NO)_3_ as the chlorine‐free Ru precursor, then statically placed at room temperature for 4 h, dried for 12 h at 60 °C and calcined at 450 °C for 4 h in a muffle furnace at a ramp rate of 5 °C min^−1^, the obtained catalysts were labeled as Ru/REs‐Co_3_O_4_‐450 (abbreviated as Ru/REsCo in the figures). Moreover, the aged Ru/La‐Co_3_O_4_‐750 was prepared through a second calcination (750 °C for 4 h at a rate of 2 °C min^−1^) of fresh Ru/La‐Co_3_O_4_‐450.

### Evaluation of Catalytic Performance

Catalytic combustion of mixed LHs was evaluated in a fixed‐bed quartz tube reactor with an inner diameter of 10 mm. 200 mg of catalyst (60–80 mesh) was fixed in the vertical center of the quartz tube with quartz cores as a separator while quartz sand layers (20–40 mesh) were filled at the top and bottom of catalysts. Mixed LHs (2.5 vol.% methane, 2.5 vol.% ethane, and 2.5 vol.% propane in Ar) and air were controlled by two mass flow meters, the concentration of mixed LHs was 750 ppm and the total flow rate was controlled at 50 mL min^−1^, the corresponding weight hourly space velocity (WHSV) was 15 000 mL/g^−1^·h. 20 ppm SO_2_ (by a mass flow meter), 5 or 10 vol.% H_2_O (by a temperature‐controlled bubbler) were introduced into the mixed gases when used. The effluent gases were analyzed using an online gas chromatograph (GC) equipped with a flame ionization detector (FID) using a PLOT Q capillary column.

### Characterizations of Catalysts

Different characterizations were used to determine the physical and chemical properties of the catalysts. Specifically included: High‐resolution transmission electron microscopy (HRTEM), Field emission scanning electron microscopy (FESEM), Powder X‐ray diffraction patterns (XRD), Raman, Temperature‐programmed reduction by hydrogen (H_2_‐TPR), in situ CO chemisorption diffuse reflective infrared Fourier transform spectroscopy (CO‐DRIFTS), X‐ray photoelectron spectra (XPS), X‐ray Adsorption Spectroscopy (XAS), Inductively coupled plasma atomic emission spectroscopy (ICP‐AES), in situ propane oxidation diffuse reflective infrared Fourier transform spectroscopy (C_3_H_8_‐DRIFTS), Temperature‐programmed reduction by propane (C_3_H_8_‐TPR), and CO pulse chemisorption.

## Conflict of Interest

The authors declare no conflict of interest.

## Author Contributions

B.G. and W.D. contributed equally to this work. B.G. performed methodology, Investigation, data curation, and wrote the final manuscript. W.D. performed conceptualization, investigation, software, and visualization. H.X. performed investigation, software. K.S. performed investigation, methodology. L.W. performed investigation, methodology. B.Q. performed formal analysis, methodology. Q.N. performed investigation, funding acquisition, software. A.W. performed validation, and formal analysis. Y.G. performed supervision, funding acquisition, data curation, and investigation. W.Z. performed methodology, formal analysis. Y.G. wrote, reviewed, and edited the final manuscript. Q.D. performed conceptualization, data curation, project administration, resources, validation, and wrote, reviewed, and edited the final manuscript.

## Supporting information



Supporting Information

## Data Availability

The data that support the findings of this study are available from the corresponding author upon reasonable request.
