# Supplementary material for: Thermally Reconstructed Ru/La‐Co3O4 Nanosheets with Super Thermal Stability for Catalytic Combustion of Light Hydrocarbons: Induced Surface LaRuO3 Active Phase
Source: Adv Sci (Weinh). 2025 Mar 16;12(18):2414919. doi: 10.1002/advs.202414919 (PMC12079328; doi:10.1002/advs.202414919)
Supplement: Supplementary file 1 — Supporting Information [file ADVS-12-2414919-s001.docx]

**Supporting Information**

**Thermally Reconstructed Ru/La-Co_3_O_4_ Nanosheets with Super Thermal Stability for Catalytic Combustion of Light Hydrocarbons: Induced Surface LaRuO_3_ Active Phase**

**Biao Gao ^a†^, Wei Deng ^b†^, Hangqi Xia ^c^, Kai Shan ^d^, Li Wang ^a^*, Boyuan Qiao ^c^**, **Qiang Niu ^c^, Aiyong Wang ^a^, Yun Guo ^a^*, Wangcheng Zhan ^a^, Yanglong Guo ^a^ and Qiguang Dai ^a^***

^a^ State Key Laboratory of Green Chemical Engineering and Industrial Catalysis, Research Institute of Industrial Catalysis, School of Chemistry and Molecular Engineering, East China University of Science and Technology, Shanghai 200237, PR China

^b^ School of Optoelectronic Materials and Technology, Jianghan University, Wuhan 430056, PR China

^c^ Electric Power and Metallurgy Group Co. Ltd., Ordos 016064, Inner Mongolia, P. R. China

^d^ Zhejiang Wild Wind Pharmaceutical Co., Ltd., Zhejiang 322105, P. R. China

* E-mail: daiqg@ecust.edu.cn (Q.G. Dai), wangli@ecust.edu.cn (L. Wang), yunguo@ecust.edu.cn (Y. Guo).

**^†^** Contributed equally to this work

**Summary of the supporting information: 31 pages, 18 figures**

**Contents of Supporting Information**

Catalyst Characterizations and Preparation 1

[Figure S1 5](#__RefHeading___Toc188637204)

[Figure S2 6](#__RefHeading___Toc188637205)

[Figure S3 7](#__RefHeading___Toc188637206)

[Figure S4 8](#__RefHeading___Toc188637207)

[Figure S5 9](#__RefHeading___Toc188637208)

[Figure S6 10](#__RefHeading___Toc188637209)

[Figure S7 11](#__RefHeading___Toc188637210)

[Figure S8 12](#__RefHeading___Toc188637211)

[Figure S9 13](#__RefHeading___Toc188637212)

[Figure S10 15](#__RefHeading___Toc188637213)

[Figure S11 16](#__RefHeading___Toc188637214)

[Figure S12 17](#__RefHeading___Toc188637215)

[Figure S13 18](#__RefHeading___Toc188637216)

[Figure S14 19](#__RefHeading___Toc188637217)

[Figure S15 21](#__RefHeading___Toc188637218)

[Figure S16 23](#__RefHeading___Toc188637219)

[Figure S17 24](#__RefHeading___Toc188637220)

[Figure S18 25](#__RefHeading___Toc188637221)

[Table S1 26](#__RefHeading___Toc188637222)

[Table S2 27](#__RefHeading___Toc188637223)

[Table S3 28](#__RefHeading___Toc188637224)

[Table S4 29](#__RefHeading___Toc188637225)

# Catalyst Characterizations and Preparation

**HRTEM and FESEM**

Images of high-resolution transmission electron microscopy (HRTEM) were obtained via JEM-2100, and images of field emission scanning electron microscopy (FESEM) were obtained via GeminiSEM 500 electron microscope.

**XRD**

The powder X-ray diffraction patterns (XRD) of samples were recorded on a Rigaku D/Max-rC powder diffractometer using CuKα radiation (40 kV and 100 mA). The diffractograms were recorded within the 2θ range of 10 to 80° with a 2θ step size of 6° and a step time of 1 min.

**Raman**

Raman spectra were obtained on a Renishaw in a Viat + Reflex spectrometer equipped with a CCD detector.

**H_2_-TPR**

Temperature-programmed reduction by hydrogen (H_2_-TPR) was carried out in a conventional apparatus equipped with a TCD. The samples (20 mg) were first pretreated for 2 h at 300 °C with an N_2_ flow (30 mL/min) before being cooled to room temperature. Then the test was performed by heating the samples in a 5% H_2_/N_2_ flow (30 mL/min) at a heating rate of 10 °C/min from 50 to 750 °C.

***in situ* CO chemiadsorption Fourier transform infrared spectroscopic (CO-DRIFTS)**

*in situ* CO chemiadsorption Fourier transform infrared spectroscopic (CO-DRIFTS) was measured on a Nicolet 6700 spectrometer equipped with an MCT detector in diffuse reflection mode. The sample was first purged with Ar (60 mL/min) at 300 °C for 1 h, and background measurements were collected at 30 °C. The 5% CO/Ar (60 mL/min) was then admitted to the IR cell for 30 min at 30 °C, and FTIR spectra were finally recorded. A Nicolet 6700 Fourier transform infrared (FTIR) spectrometer equipped with a liquid-nitrogen-cooled mercury-cadmium telluride detector was used to perform in situ diffuse reflectance infrared Fourier transform spectroscopy (DRIFTS). A DRIFTS cell (Harrick, HVC-DRP) fitted with a ZnSe window was used as a reaction chamber, and the spectra were recorded within a frequency range of 4000-1000 cm^-1^ at a resolution of 4 cm^-1^ for 64 scans.

**XPS**

X-ray photoelectron spectra (XPS) of prepared samples were collected on a PHI 5000 VersaProbe system, equipped with a monochromatic Al Kα source gun (1486.6 eV) operated at an accelerating power of 15 kW. The binding energies of all elements were calibrated using the C1s XPS peak at 284.8 eV.

**XAS**

The X-ray adsorption near-edge structure (XANES) and extended X-ray absorption fine structure (EXAFS) were collected at the Co K-edge of the Shanghai Synchrotron Radiation Facility (SSRF).

**ICP-AES**

The real concentrations of Ru were measured by ICP-AES analysis on a Vanan 710 spectrometer.

***in situ* diffuse reflectance infrared Fourier transform spectroscopy (*in situ-*DRIFTS)**

*in situ* propane oxidation Fourier transform infrared spectroscopic (C_3_H_8_-DRIFTS) was measured on a Nicolet 6700 spectrometer equipped with an MCT detector in diffuse reflection mode. The sample was first purged with 20 vol% O_2_/Ar (50 mL/min) at 300 ^o^C for 2 h, and background measurements were collected at 50, 100, 150, 200, 250, and 300 ^o^C. The propane/O_2_/Ar (50 mL/min) was then admitted to the IR cell for 15 min at 50 ^o^C, and FTIR spectra were finally recorded. A Nicolet 6700 Fourier transform infrared (FTIR) spectrometer equipped with a liquid-nitrogen-cooled mercury-cadmium telluride detector was used to perform in situ diffuse reflectance infrared Fourier transform spectroscopy (DRIFTS). A DRIFTS cell (Harrick, HVC-DRP) fitted with a ZnSe window was used as a reaction chamber that allowed samples to be heated to 600 ^o^C, and the spectra were recorded within a frequency range of 4000-1000 cm^-1^ at a resolution of 4 cm^-1^ for 64 scans. A 50-mg-grain catalyst (40-60 mesh) was packed in the DRIFTS cell.

**Temperature-programmed reduction by propane (C_3_H_8_-TPR)**

Temperature-programmed reduction by propane (C_3_H_8_-TPR) was carried out in a conventional apparatus equipped with an MS. The samples (200 mg) were first pretreated for 2 h at 300 ^o^C with an Ar flow (30 mL/min) before being cooled to room temperature. Then the test was performed by heating the samples in a 5% C_3_H_8_/Ar flow (50 mL/min) at a heating rate of 10 ^o^C/min from 50 to 850 ^o^C.

**Preparation of the Ru/CeO_2_ catalyst**

The CeO_2_ nanosheets were synthesized using a precisely temperature-controlled rapid precipitation method. Specifically, 5.56 g of cerium(III) nitrate hexahydrate (Ce(NO_3_)_3_·6H_2_O) and 4.0 g of ammonium bicarbonate (NH_4_HCO_3_) were separately dissolved in 200 mL of deionized water at 30 °C. The NH_4_HCO_3_ solution was then rapidly poured into the Ce(NO_3_)_3_ solution under continuous stirring for 0.5 h, followed by static aging at 30 °C for 24 h. The resulting precipitate was collected by filtration, thoroughly washed with deionized water and ethanol, and dried at 60 °C. Finally, the product was calcined at 450 °C for 4 h in air to obtain the CeO_2_ nanosheets.

The Ru/CeO_2_ catalyst was prepared via an incipient wetness impregnation method using a chlorine-free ruthenium precursor, ruthenium(III) nitrosyl nitrate (Ru(NO)(NO_3_)_3_). After impregnation, the sample was allowed to stand for 4 h at room temperature, dried overnight at 60 °C, and calcined at 450 °C for 4 h in air.

**Preparation of the Ru/Z-500 catalyst**

The ZSM-5 with the SiO_2_/Al_2_O_3_ ratio of 500 support was synthesized via a hydrothermal method. Briefly, 50 g of deionized water, 13 mg of aluminum isopropoxide, 6.5 g of tetraethyl silicate (TEOS), and 0.3 g of sodium chloride were mixed in a Teflon liner and stirred for 2 h at room temperature until a homogeneous solution was obtained. Subsequently, 11.25 g of tetrapropyl ammonium hydroxide (TPAOH) was added as a templating agent, and the mixture was hydrolyzed under continuous stirring at room temperature for 24 h. The Teflon liner was then transferred to a high-pressure hydrothermal reactor and heated at 170 °C for 72 h. After the reaction, the crude product was collected, dried, and calcined at 550 °C for 4 h in air to obtain the Z-500.

The Ru/Z-500 catalyst was prepared using the same incipient wetness impregnation method as described above, with Ru(NO)(NO_3_)_3_ as the ruthenium precursor. After impregnation, the sample was allowed to stand for 4 h at room temperature, dried overnight at 60 °C, and calcined at 450 °C for 4 h in air.


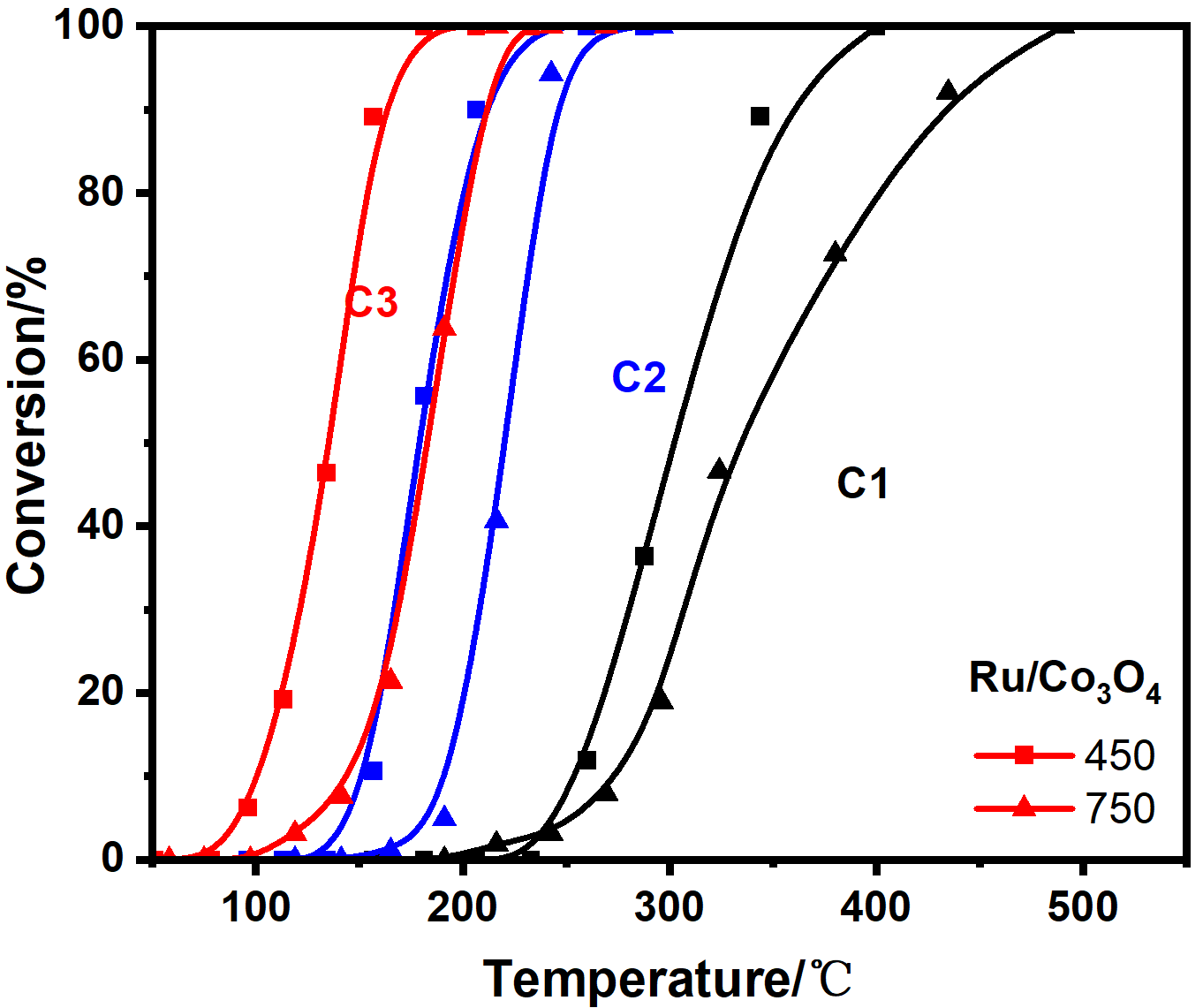

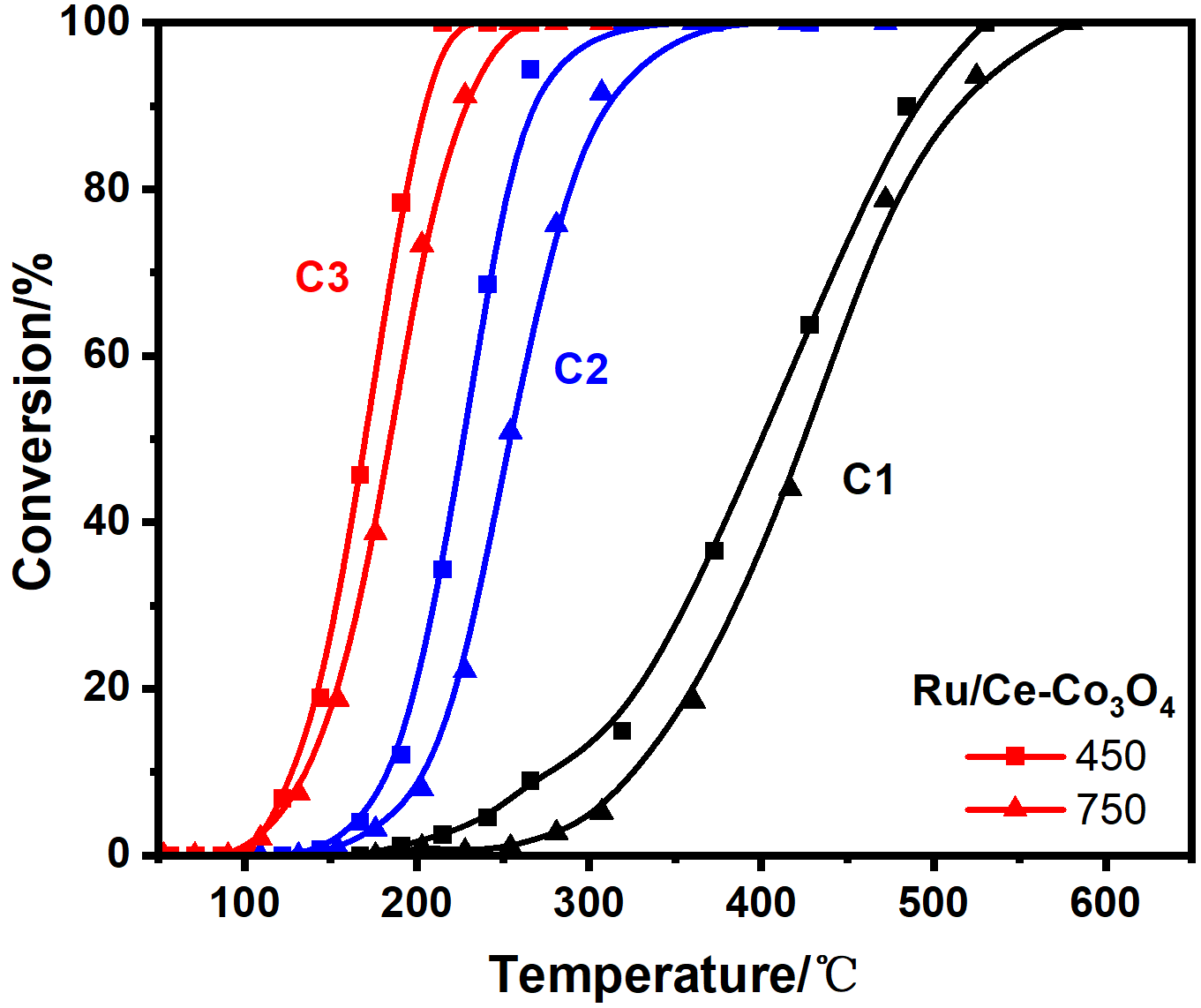

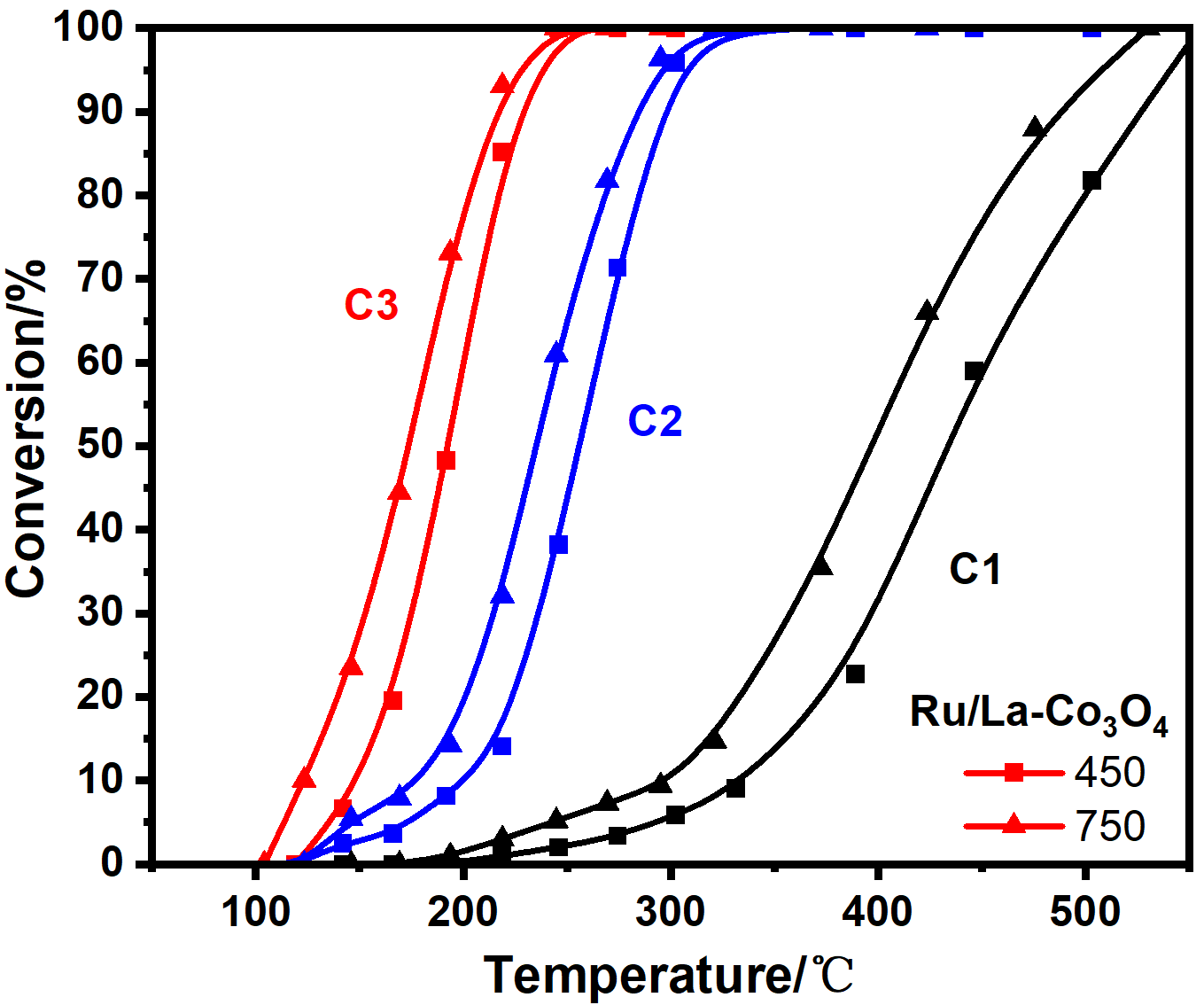

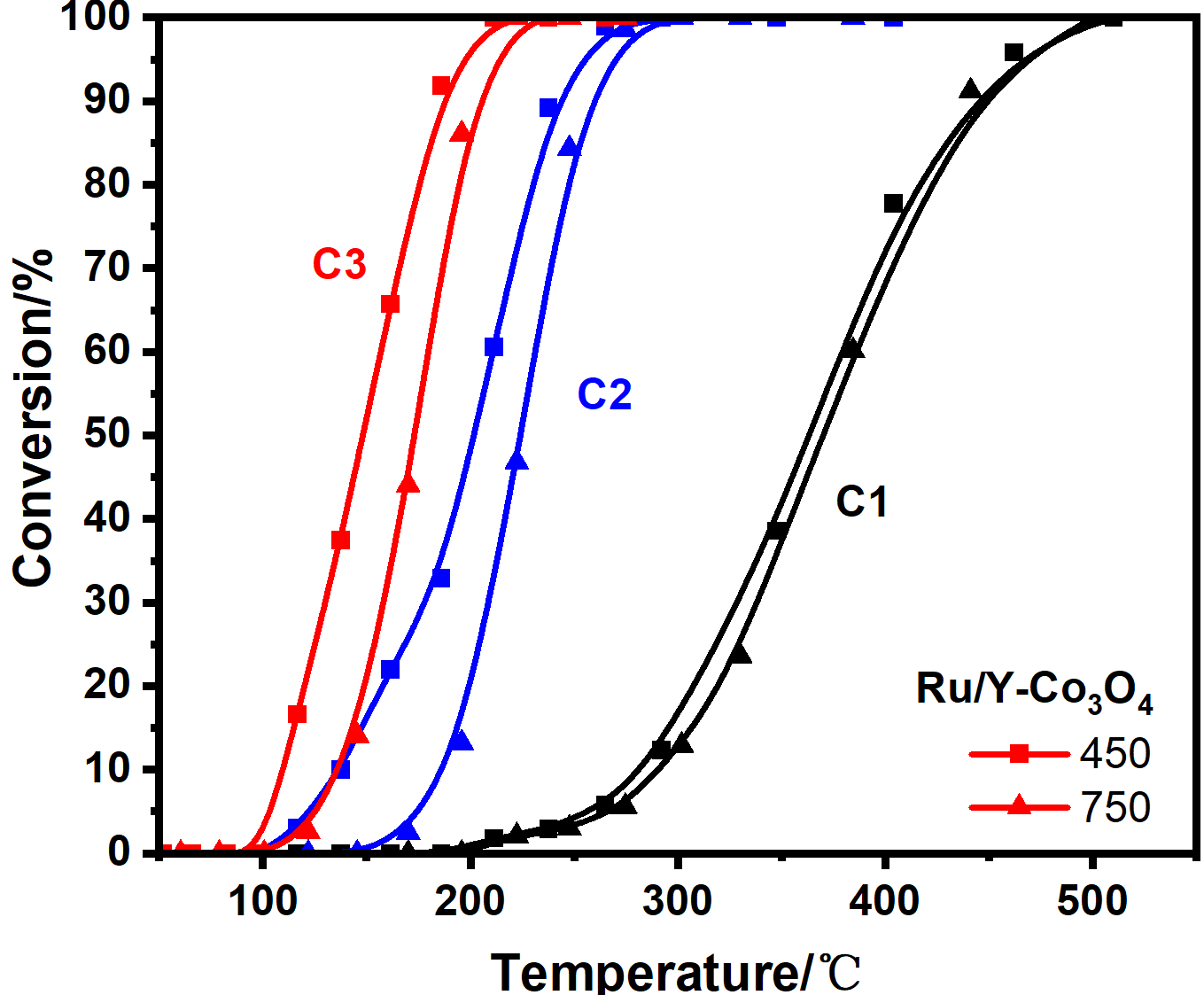


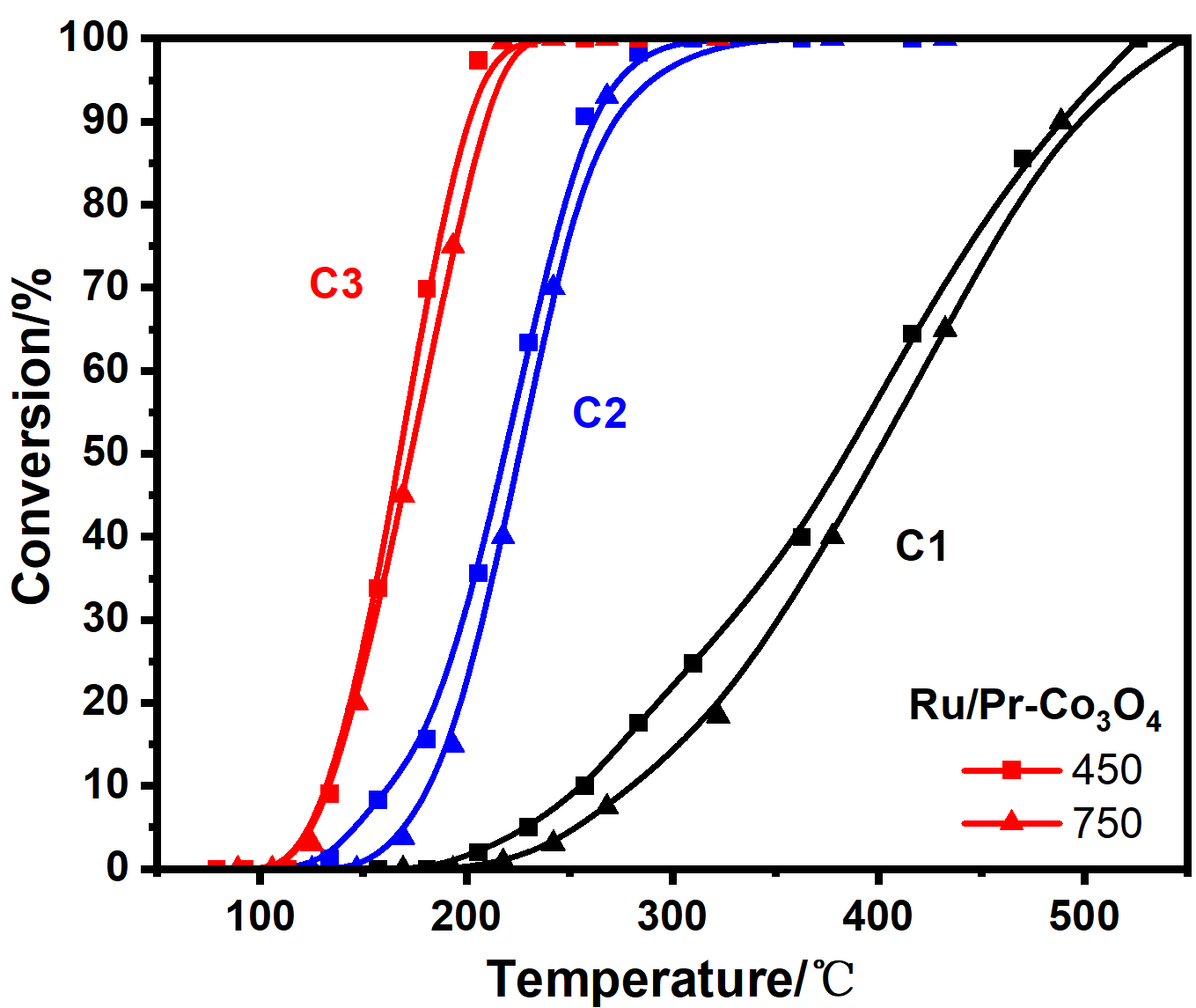

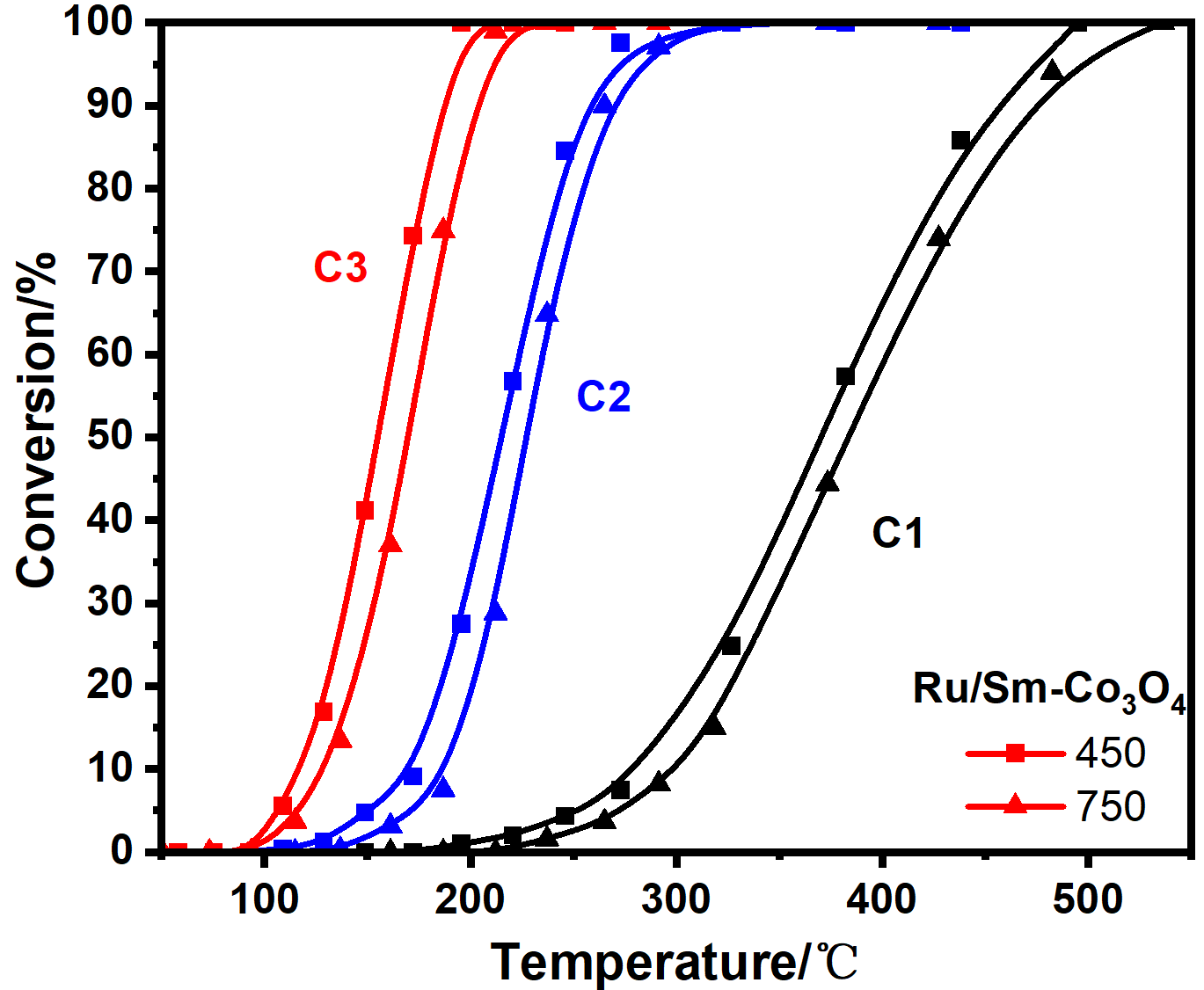


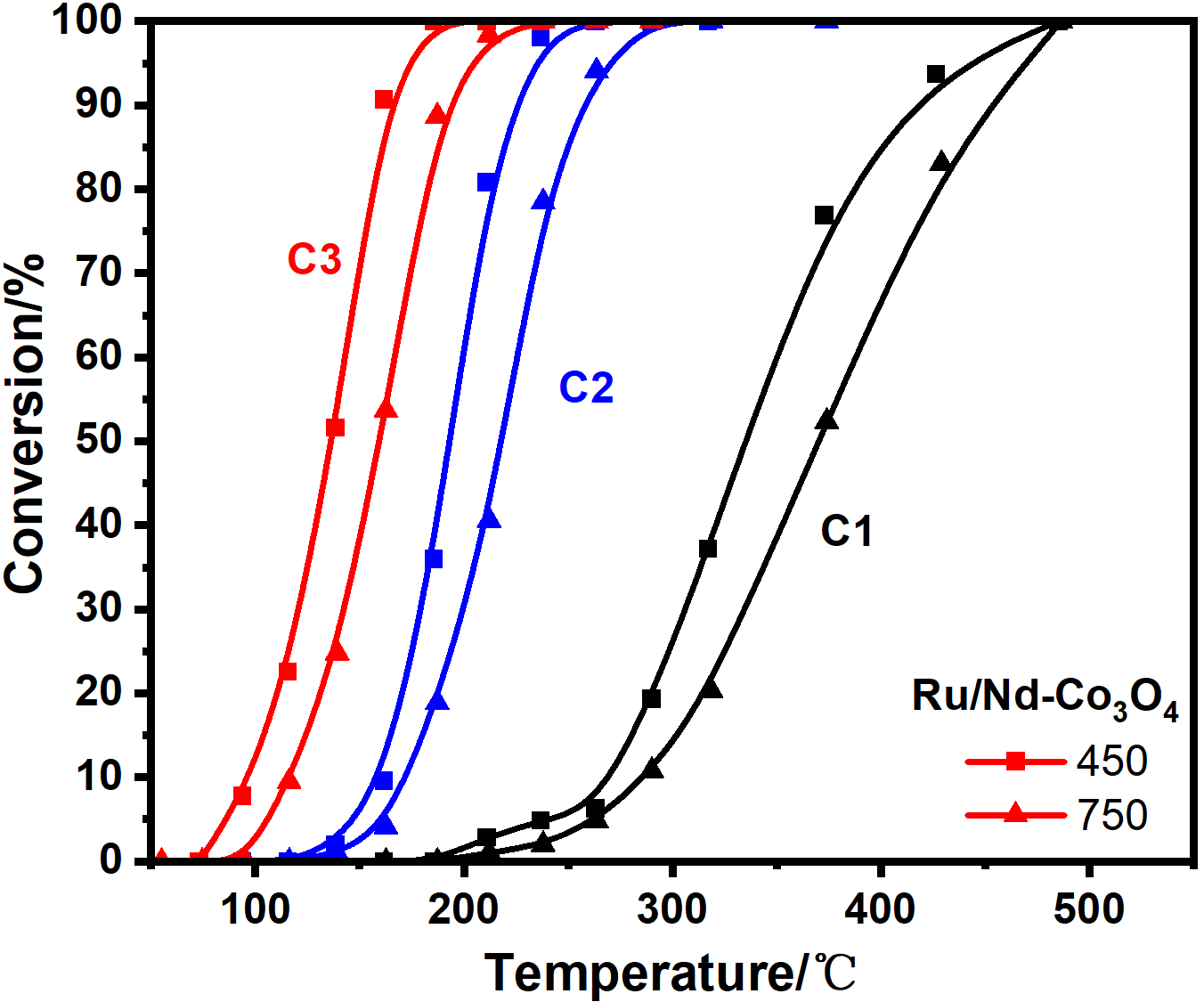

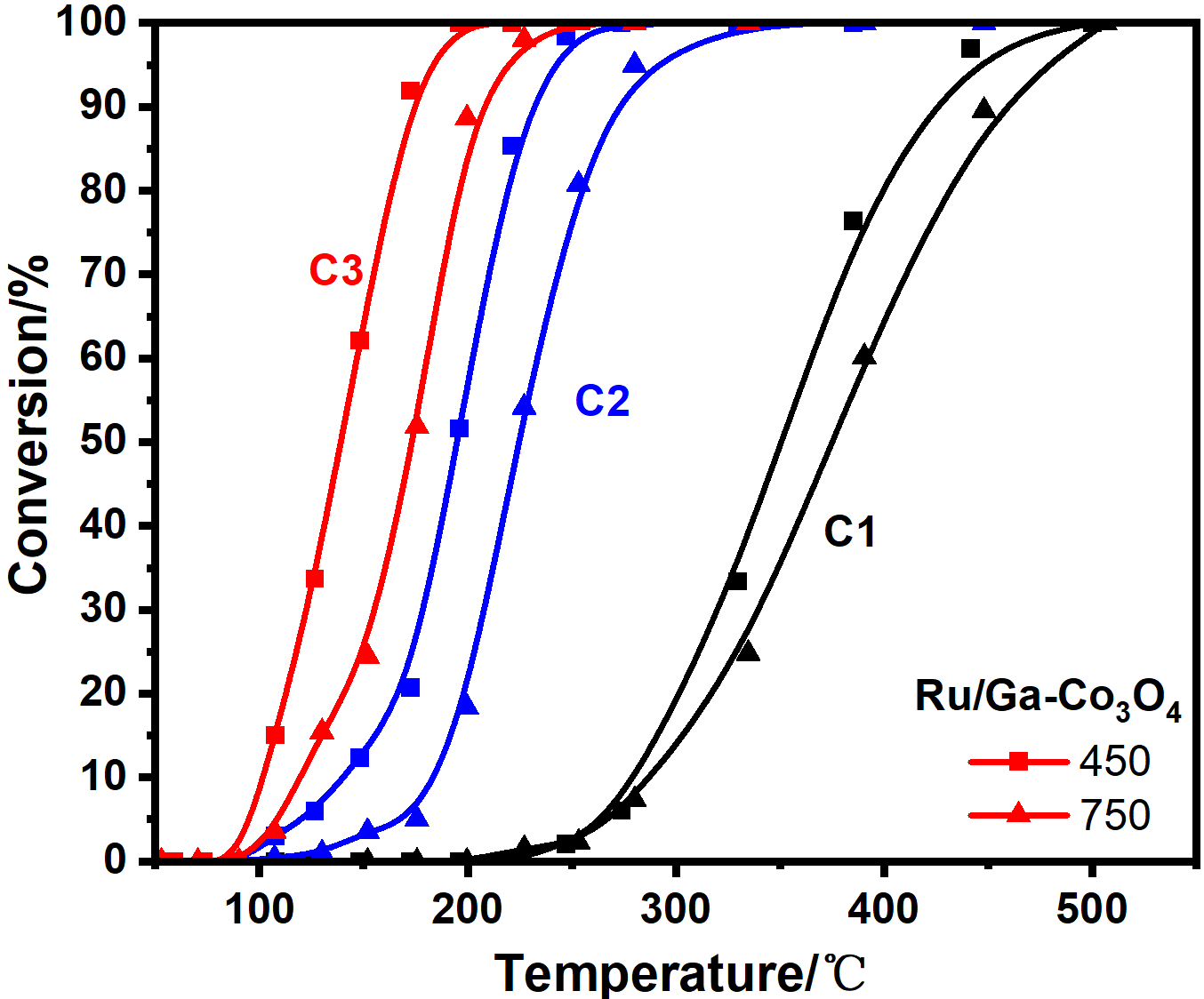


Figure S1 Light-off curves of fresh and aged (at 750 °C) Ru/REs-Co_3_O_4_ catalysts for catalytic combustion of mixed LHs.


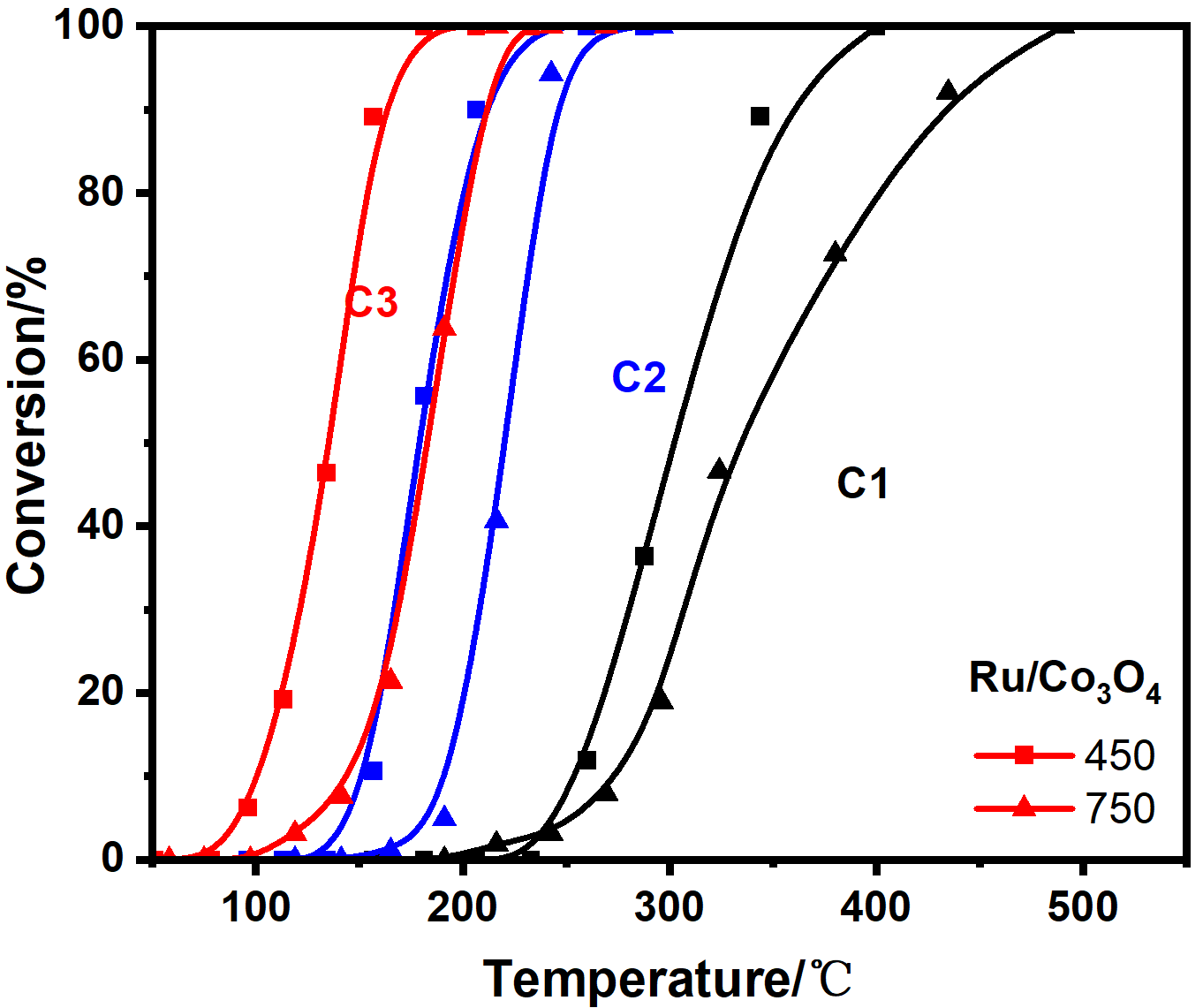

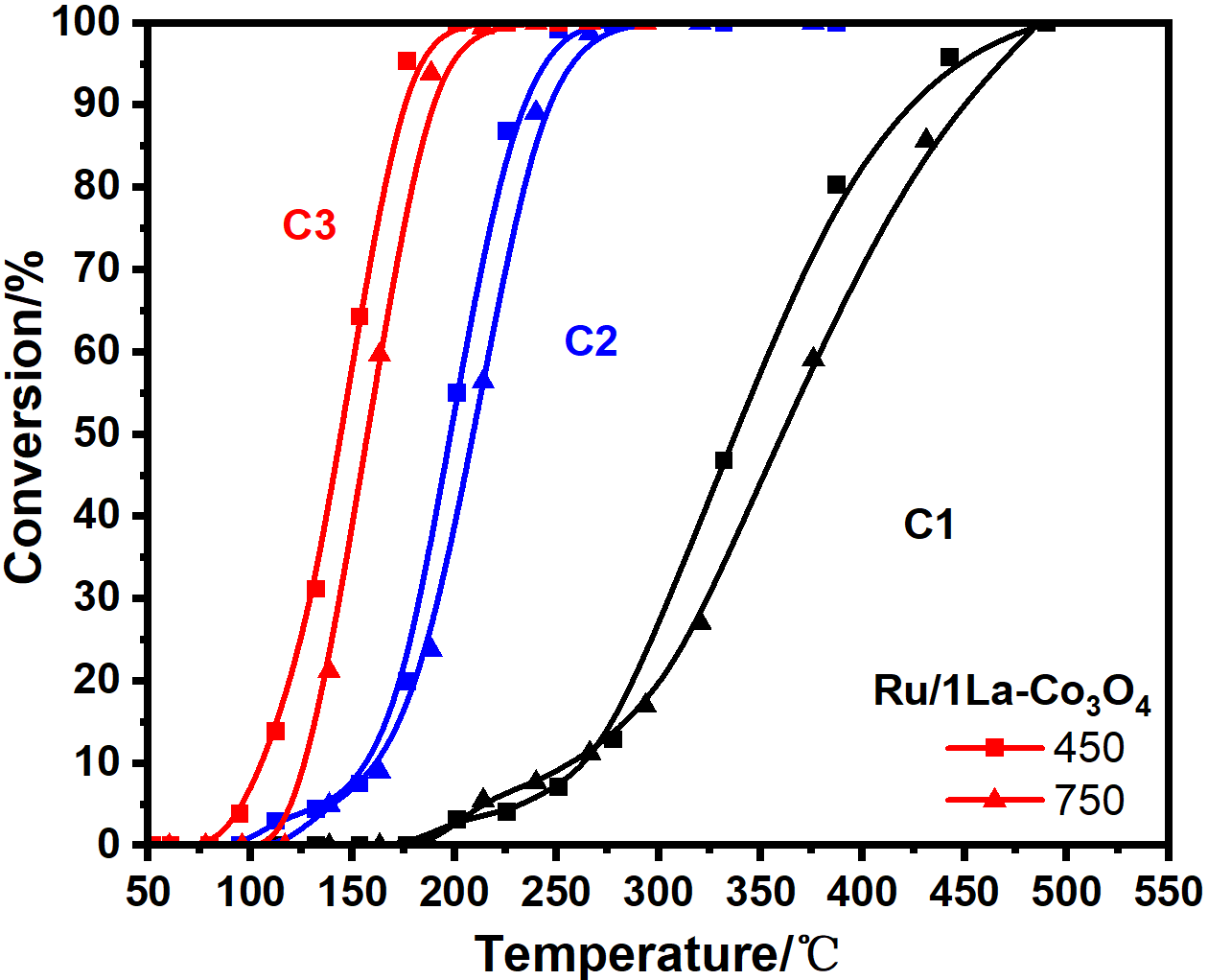


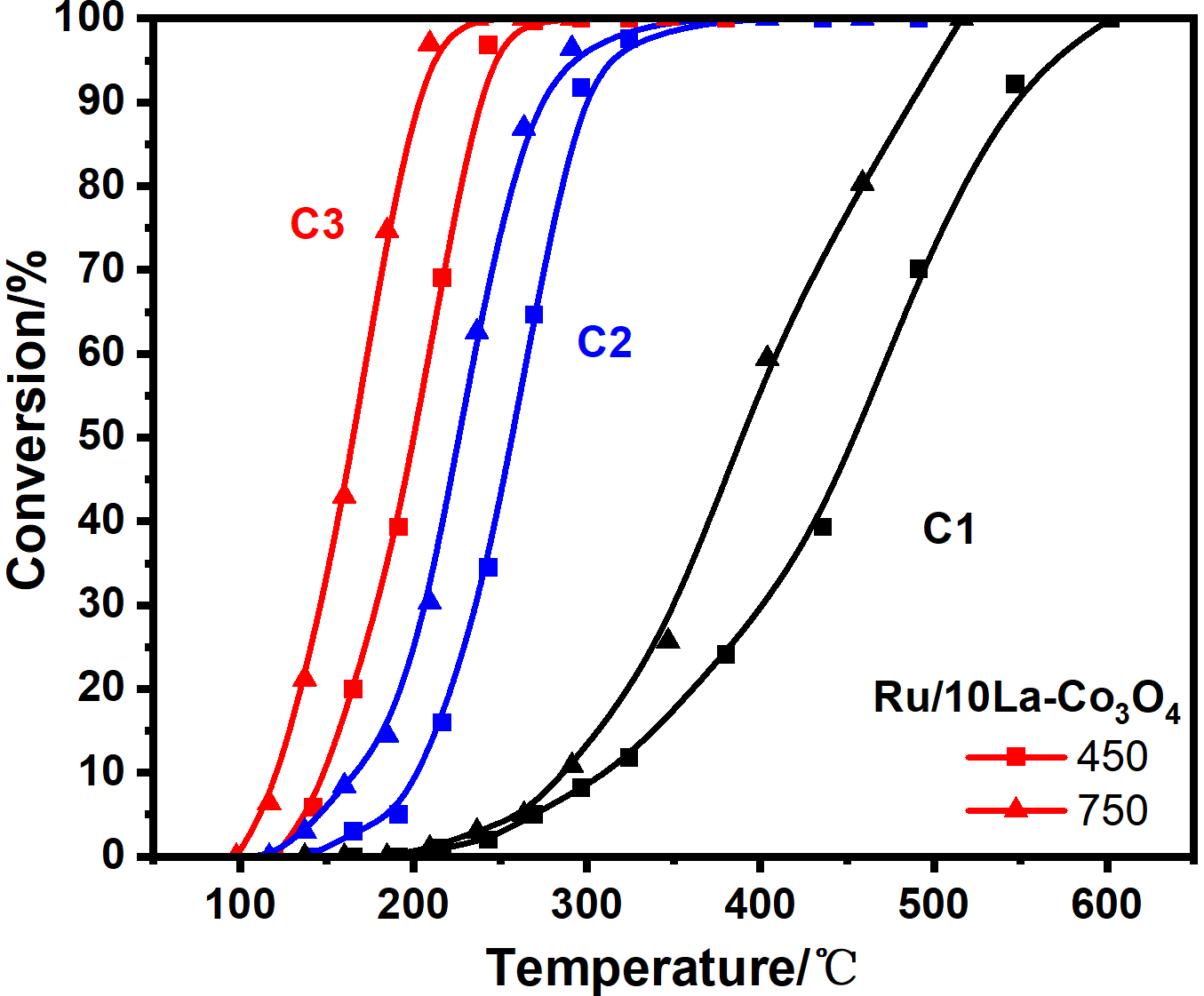

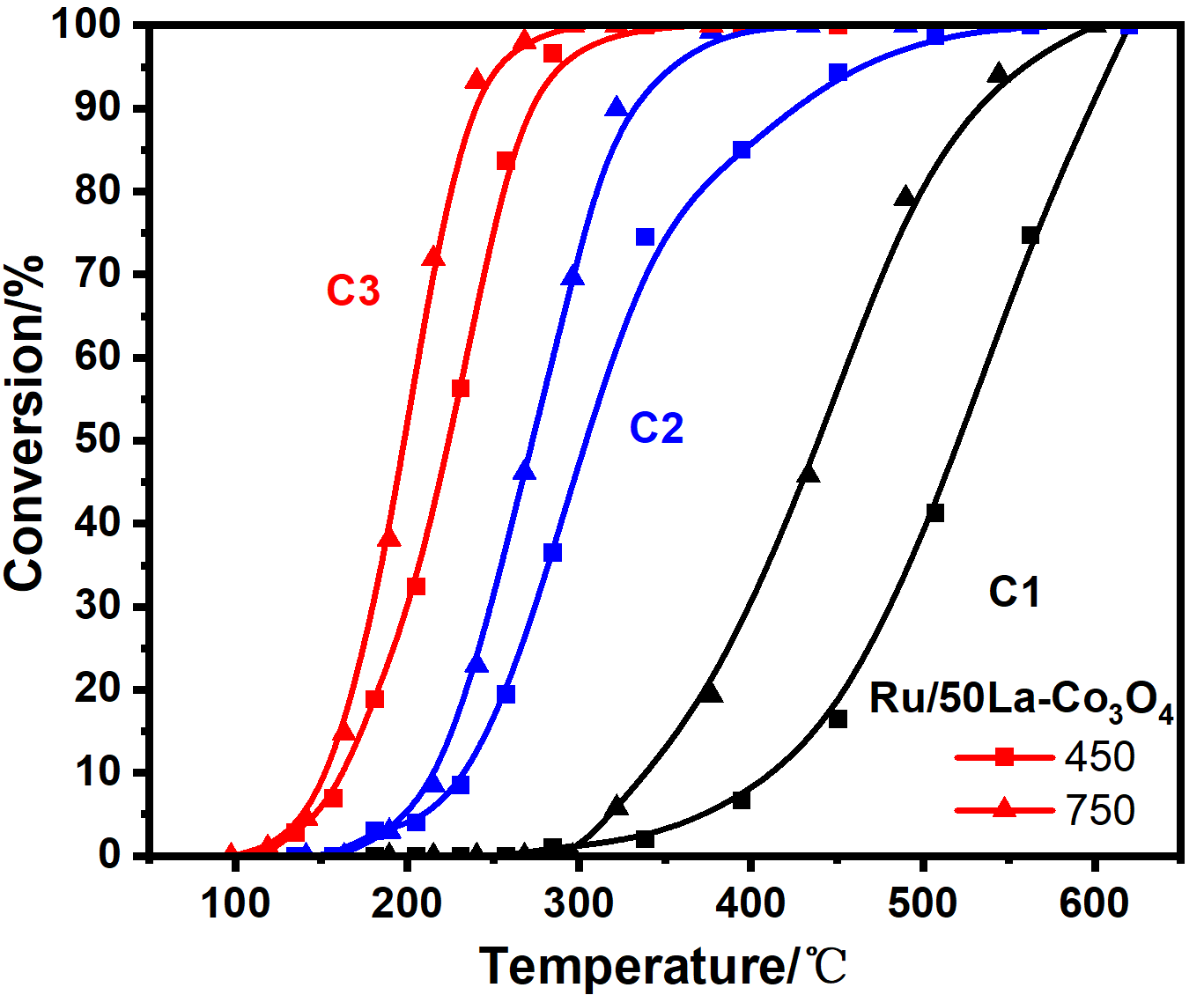


Figure S2 Light-off curves of fresh and aged (at 750 °C) 2.25 wt.% Ru/La-Co_3_O_4_ with different La content for catalytic combustion of mixed LHs.


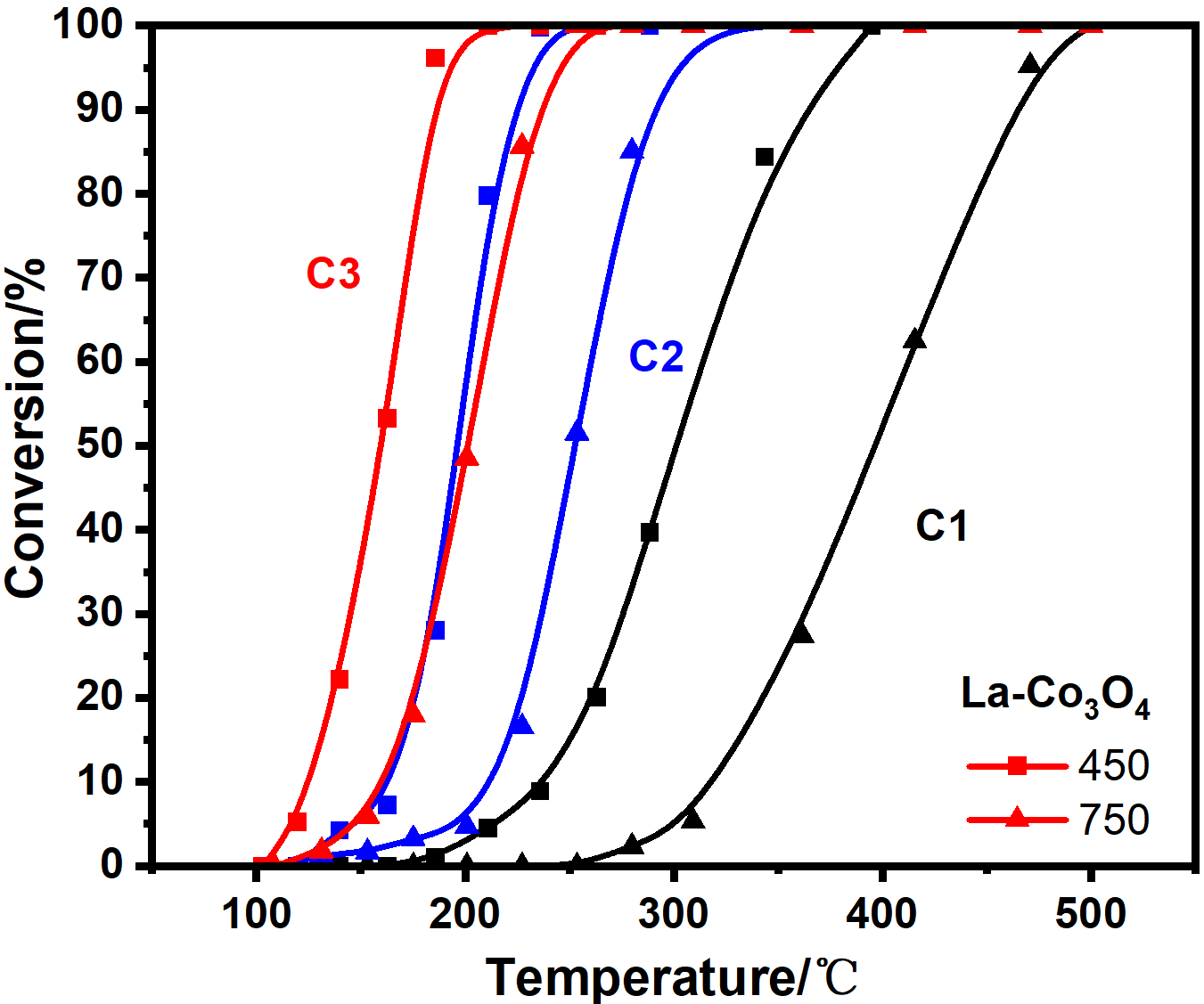

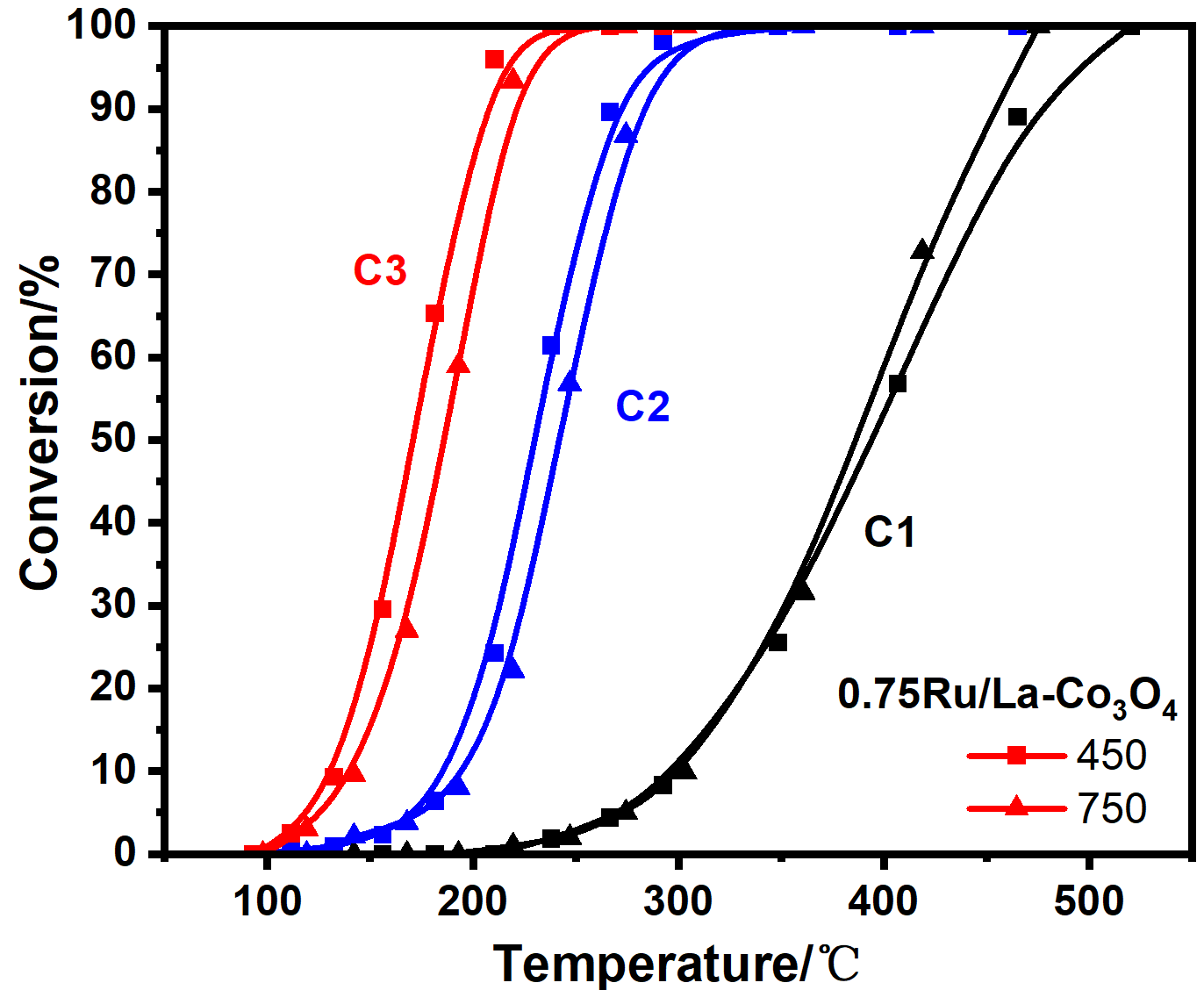


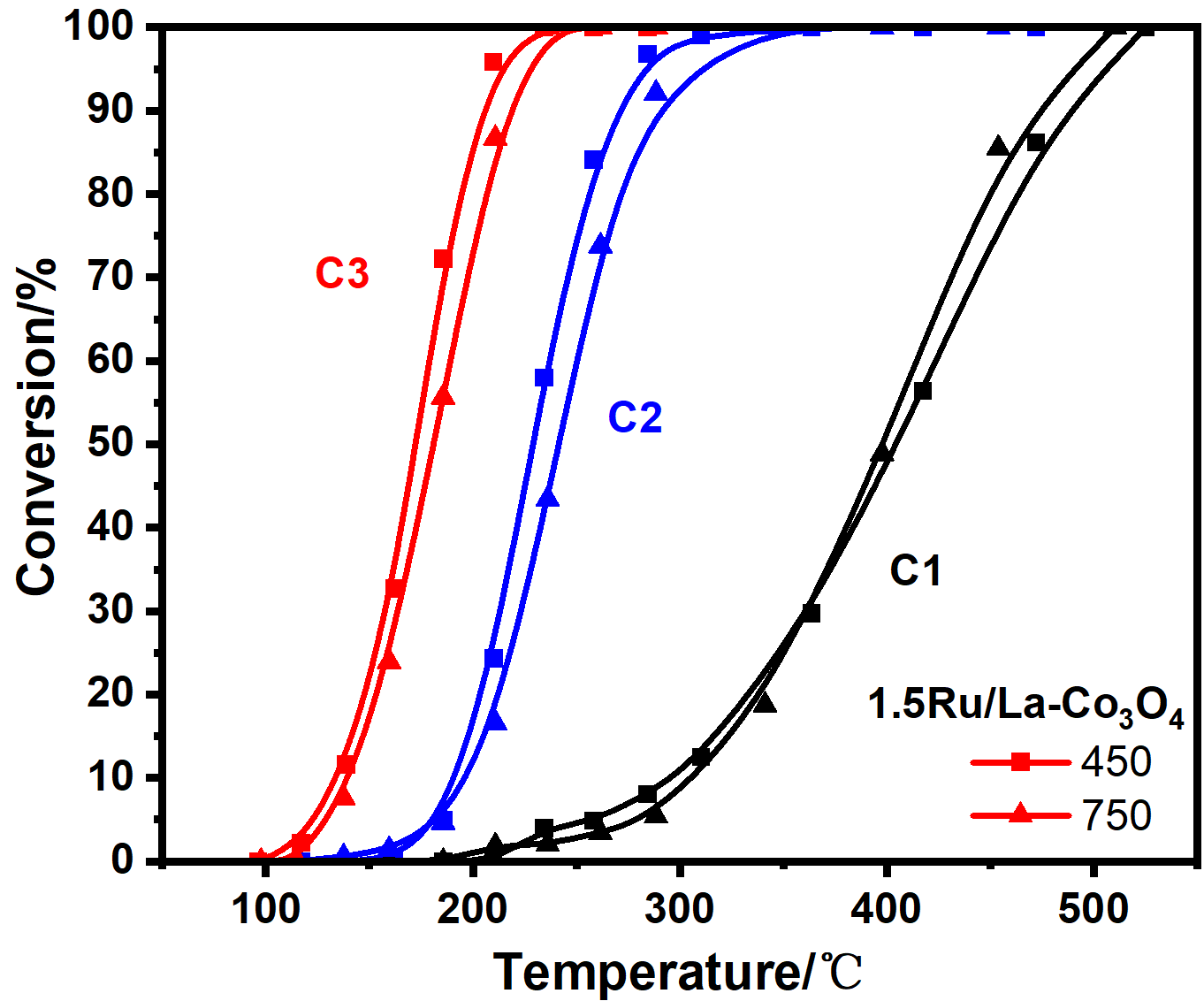

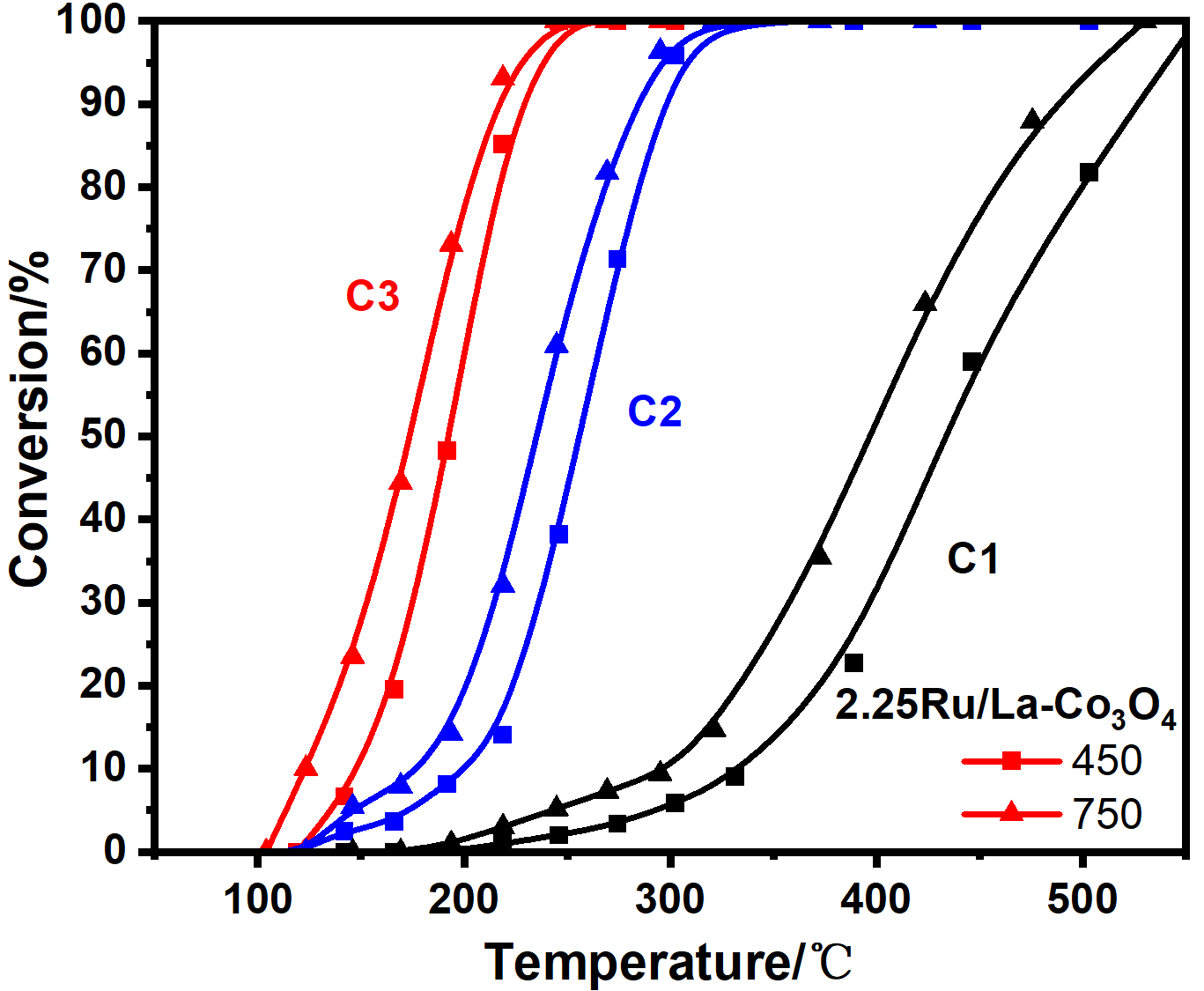


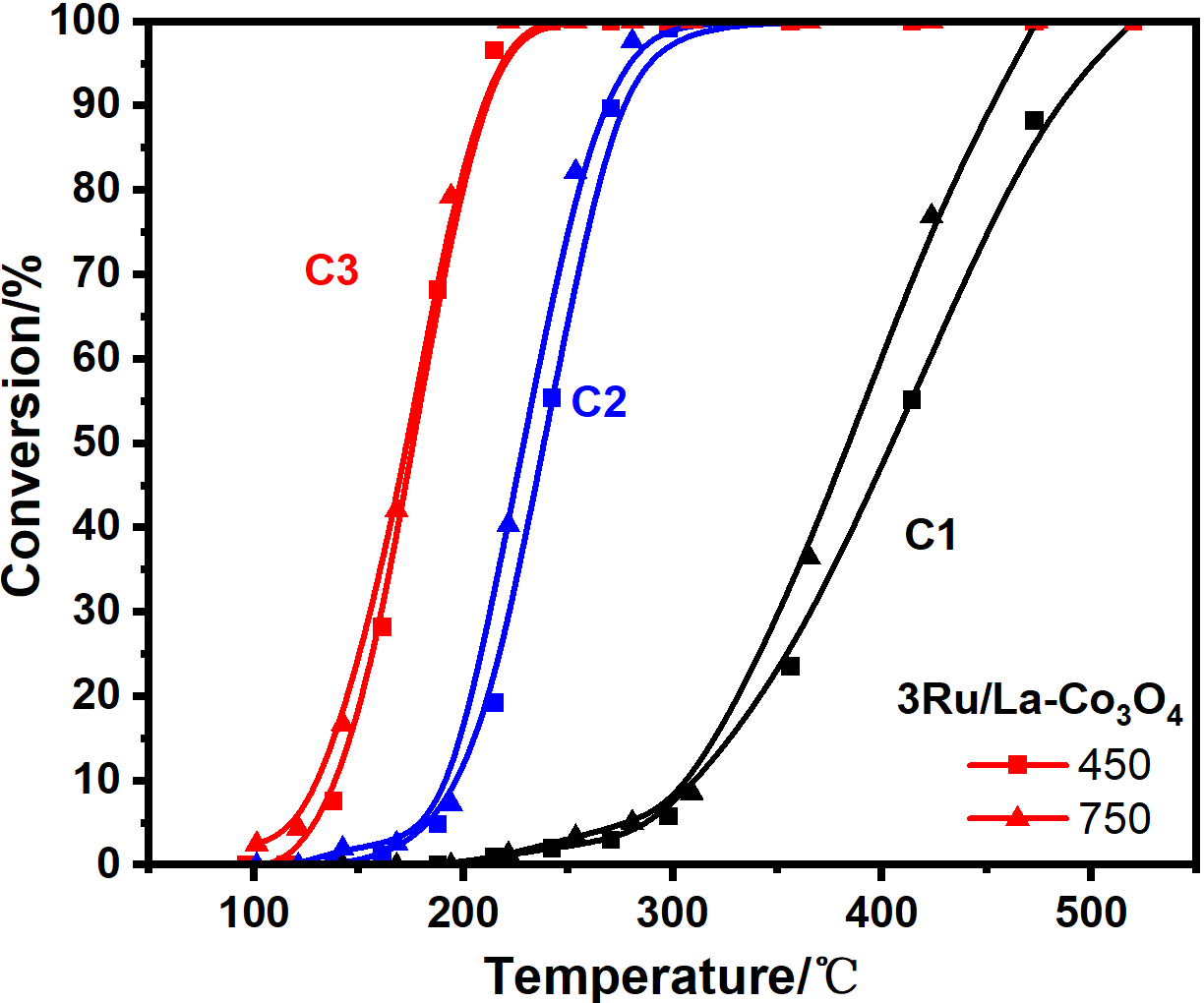

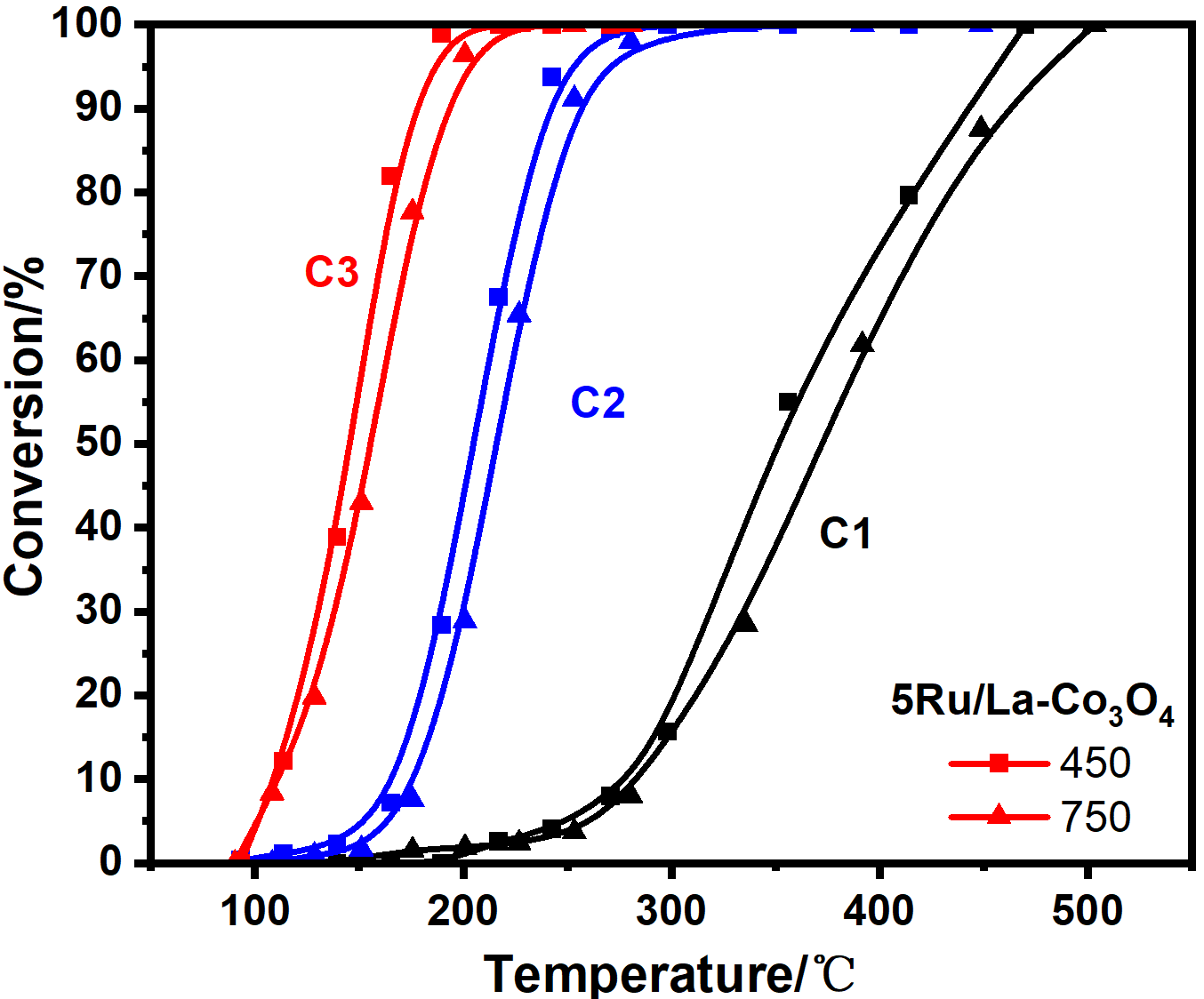


Figure S3 Light-off curves of fresh and aged (at 750 °C) Ru/5La-Co_3_O_4_ with different Ru content for catalytic combustion of mixed LHs.


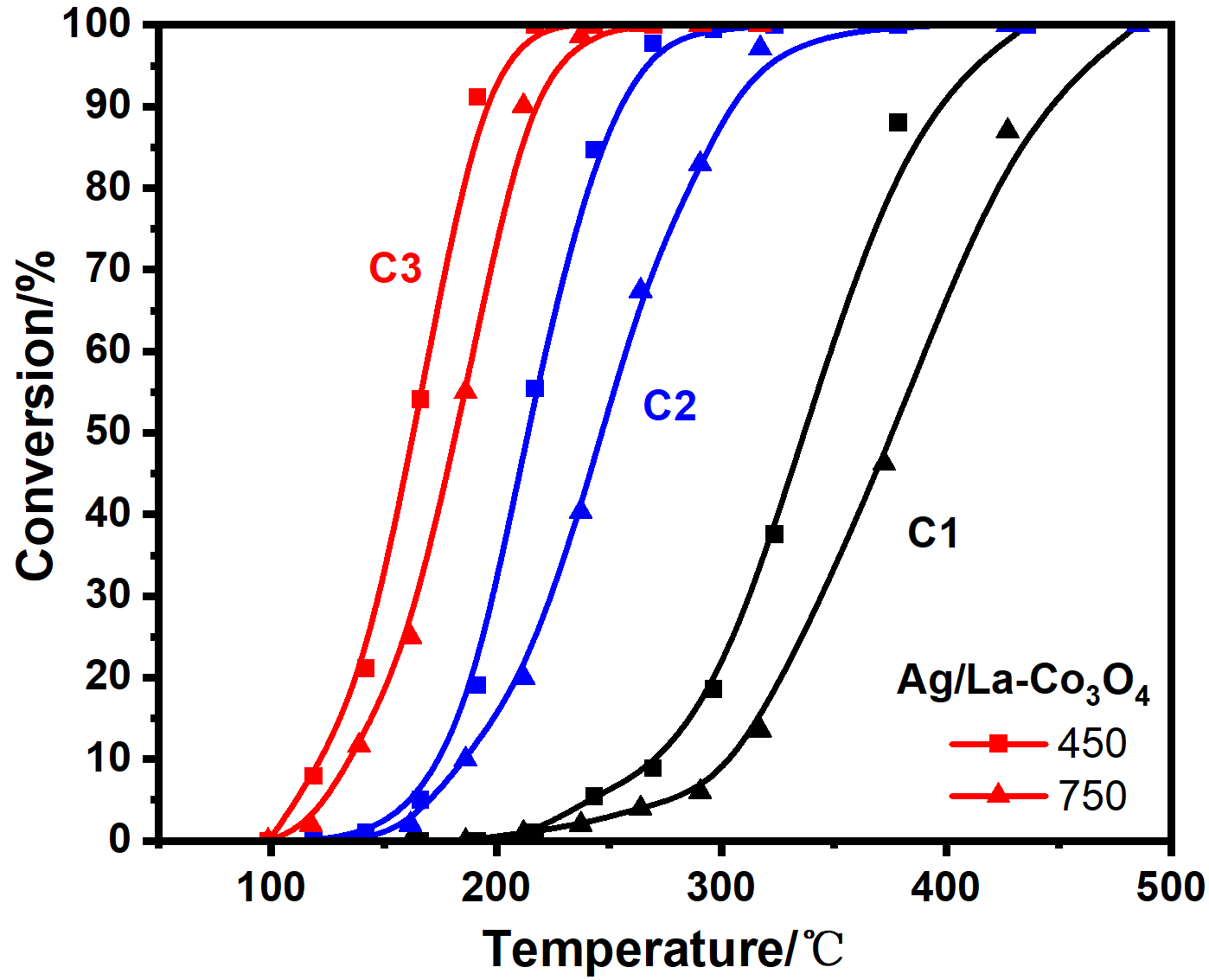


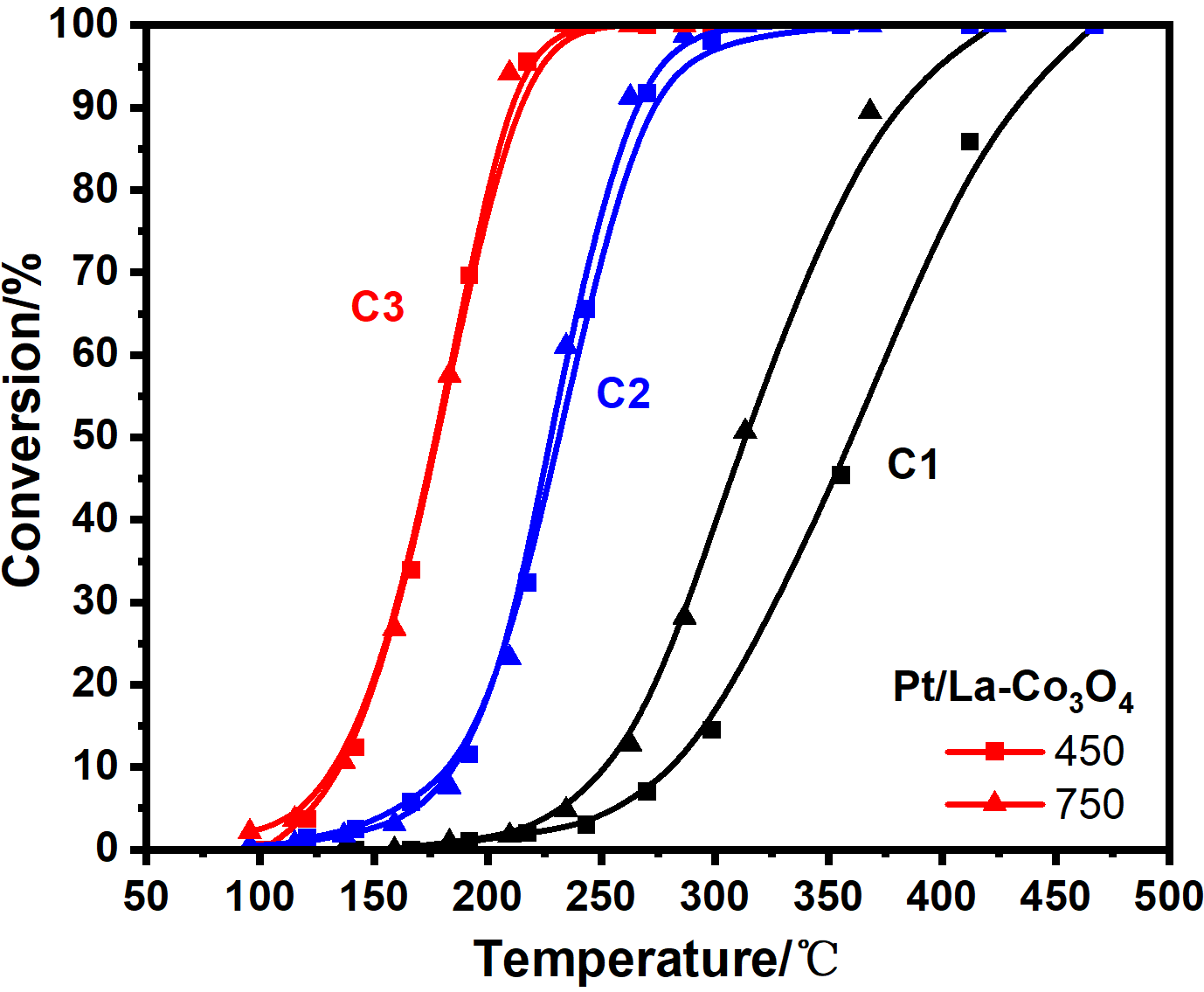


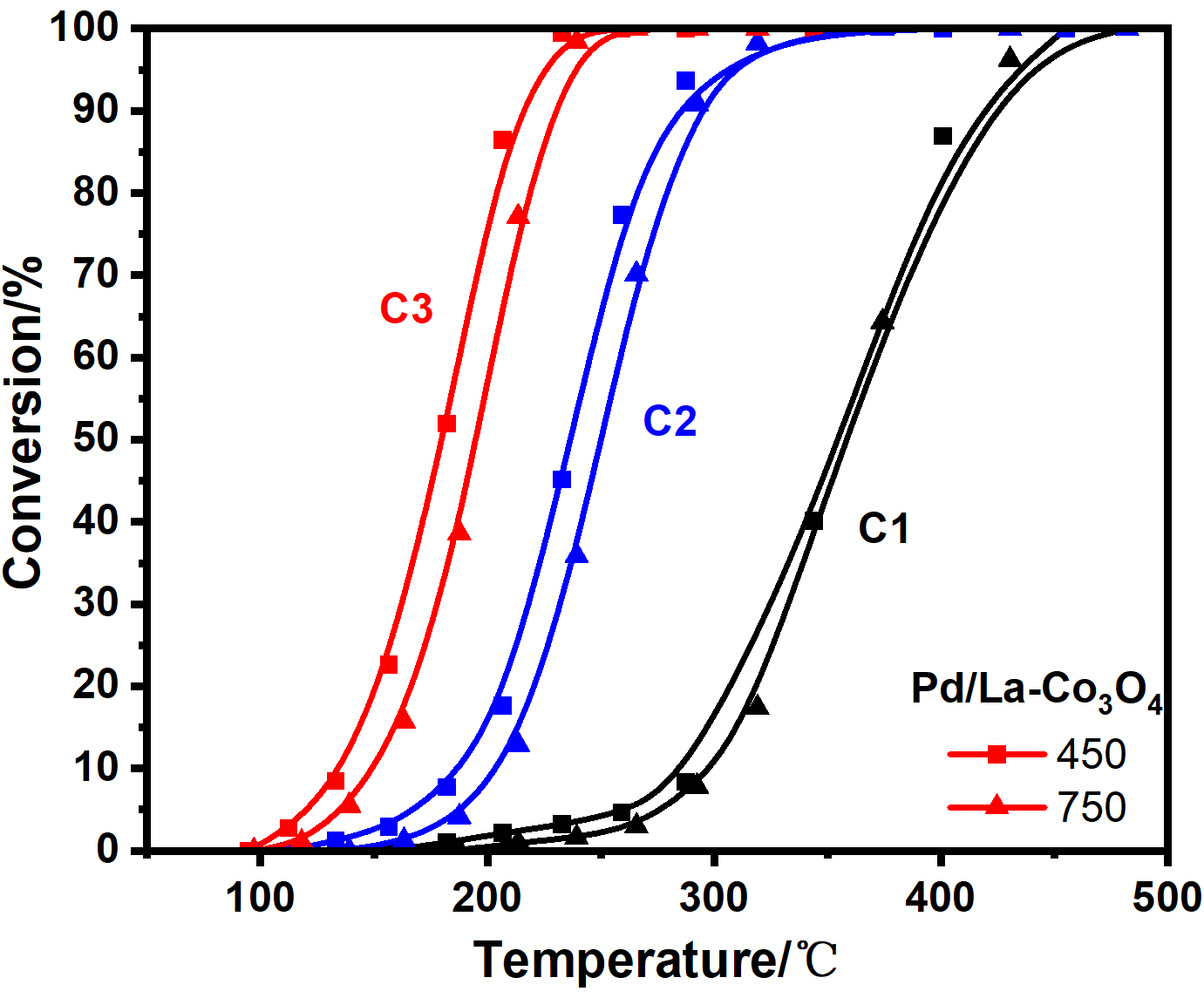


Figure S4 Light-off curves of fresh and aged (at 750 °C) precious metals supported 5La-Co_3_O_4_ (precious metals = Ag, Pd, and Pt) for catalytic combustion of mixed LHs.


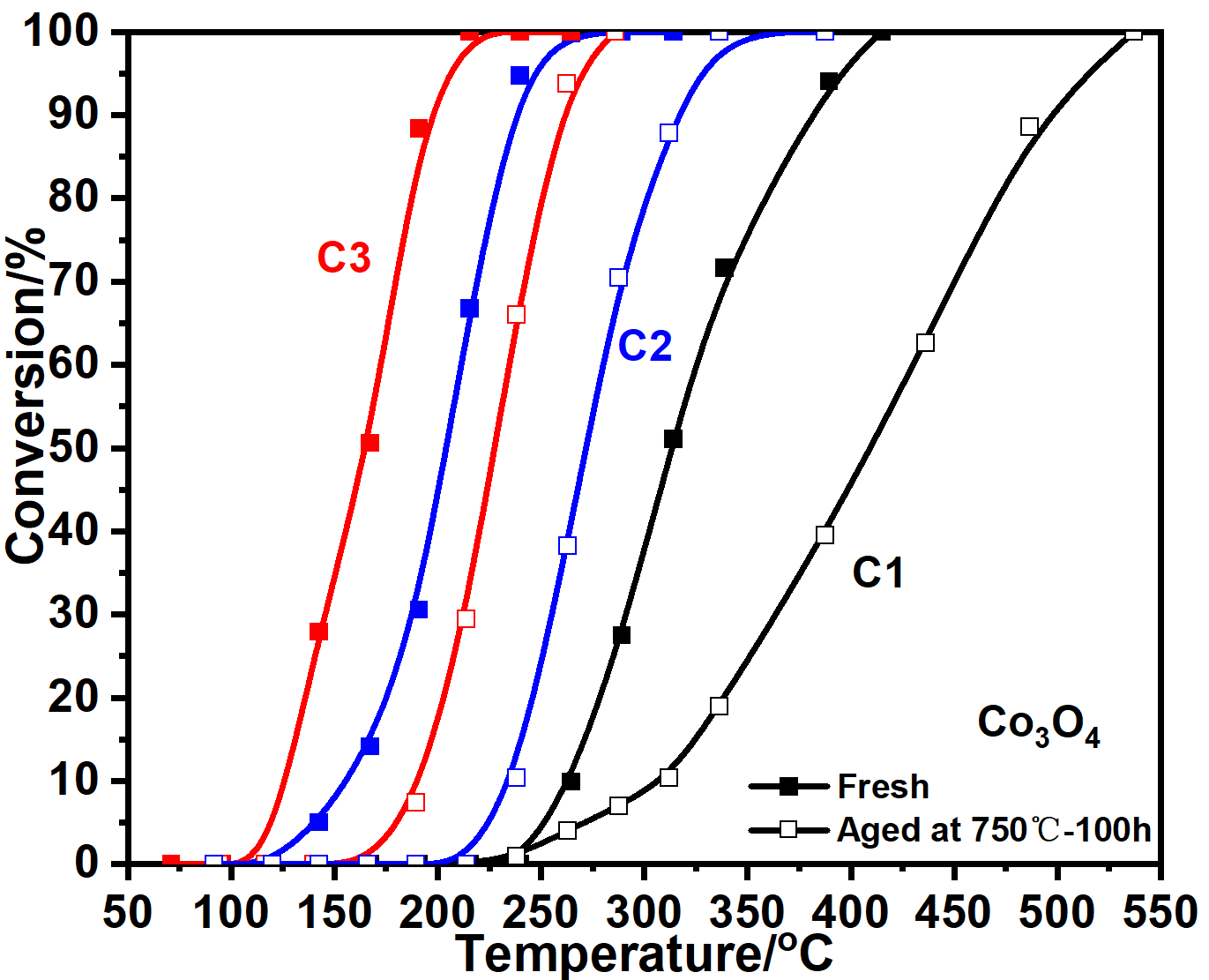

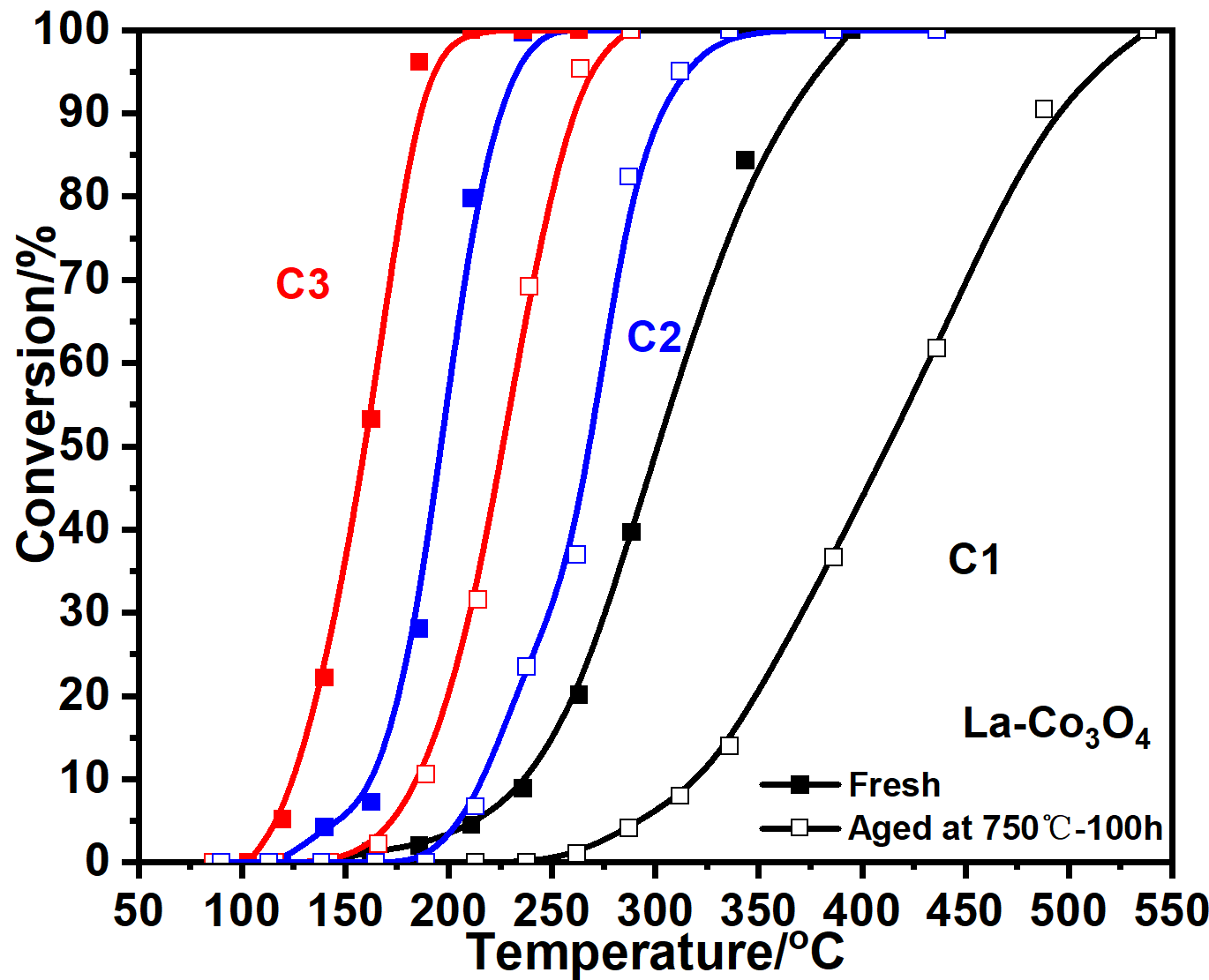


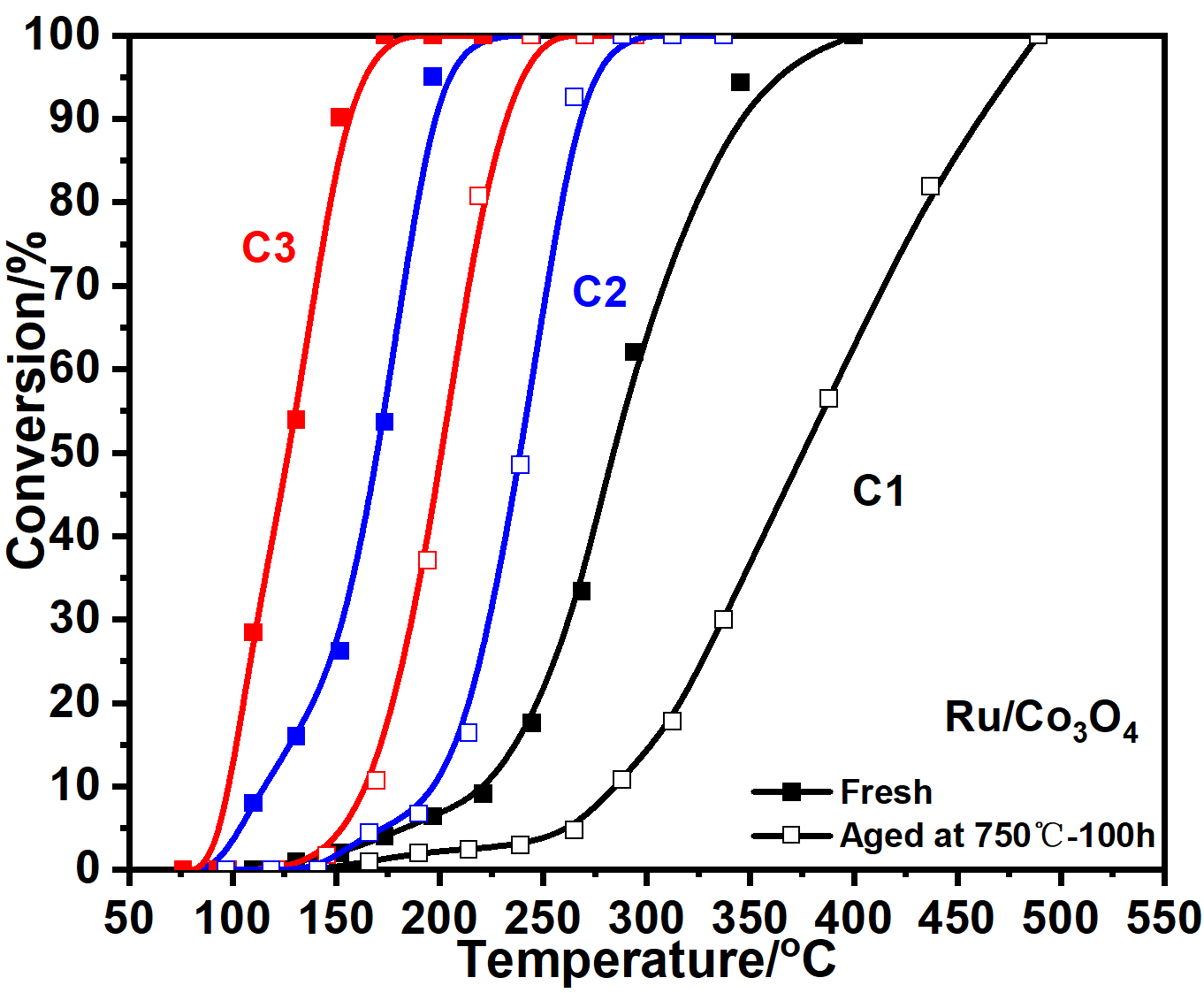

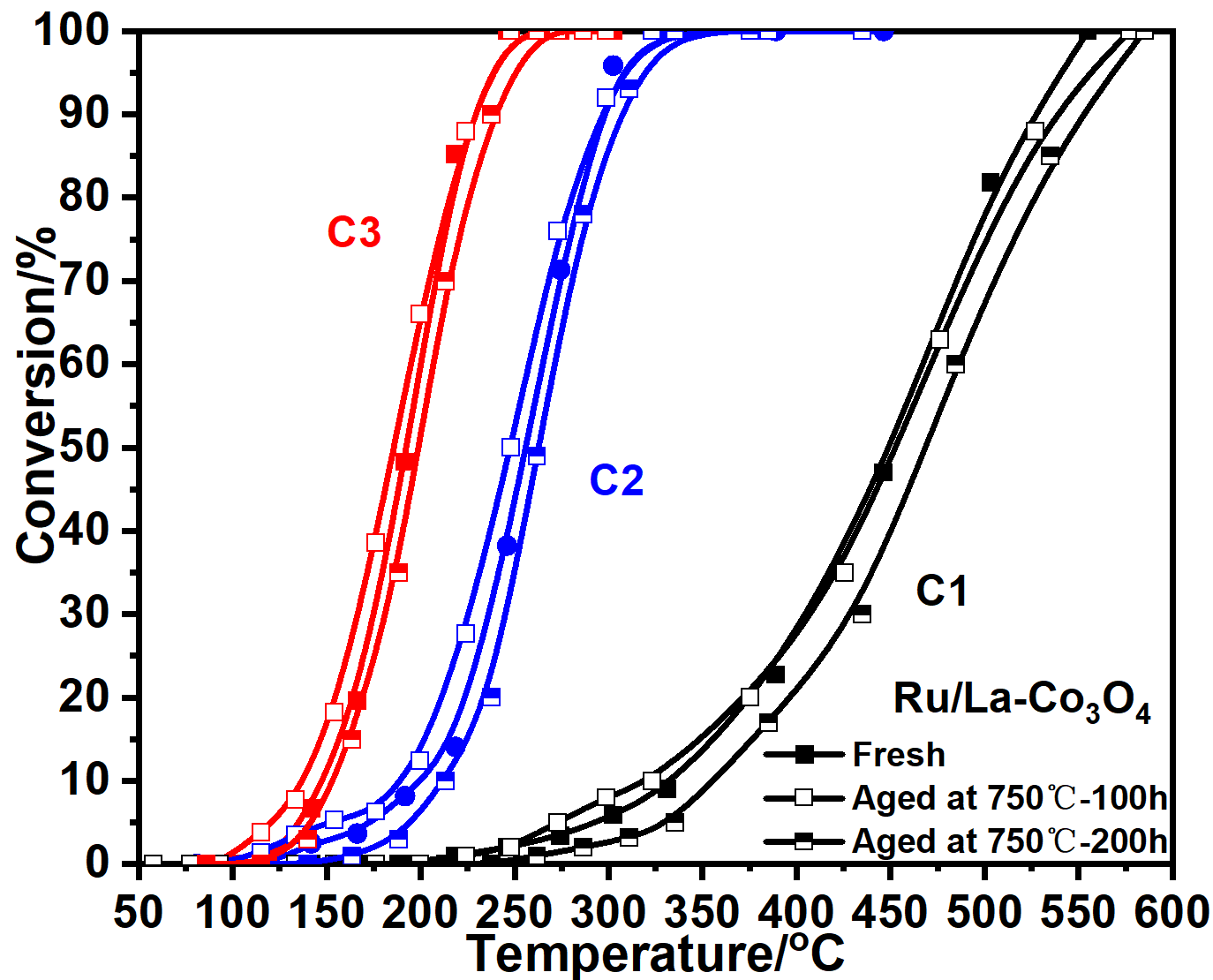


Figure S5 Light-off curves of the aged Co_3_O_4_-based catalysts at 750 °C for 100 h or 200 h for catalytic combustion of mixed LHs.


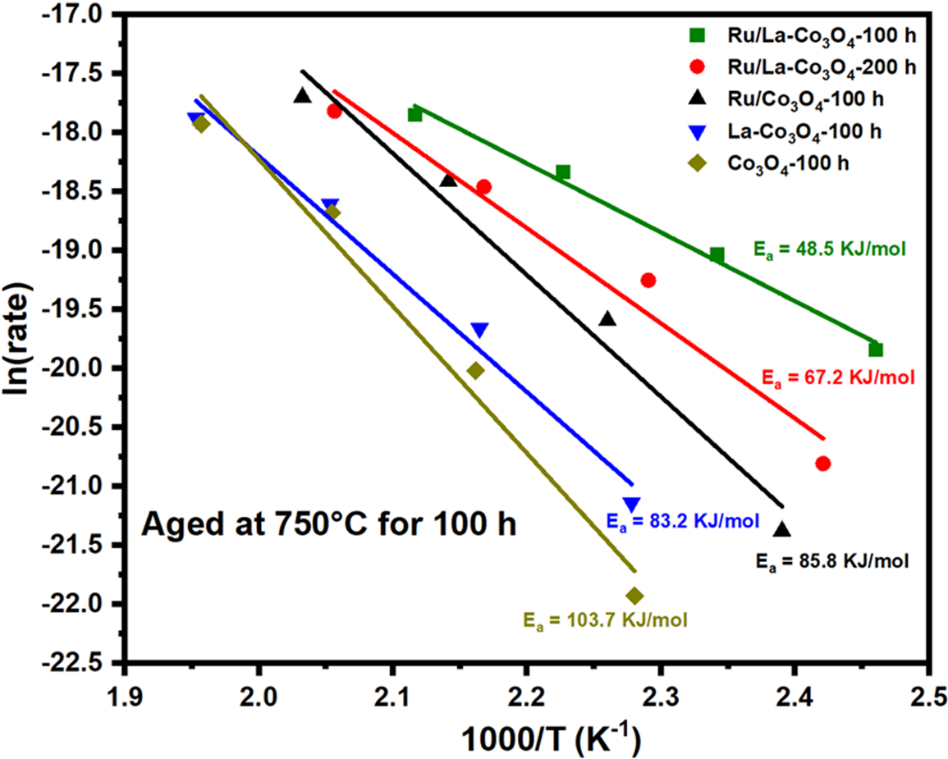


Figure S6 Arrhenius plots of the aged Co_3_O_4_-based catalysts at 750 °C for 100 h or 200 h.


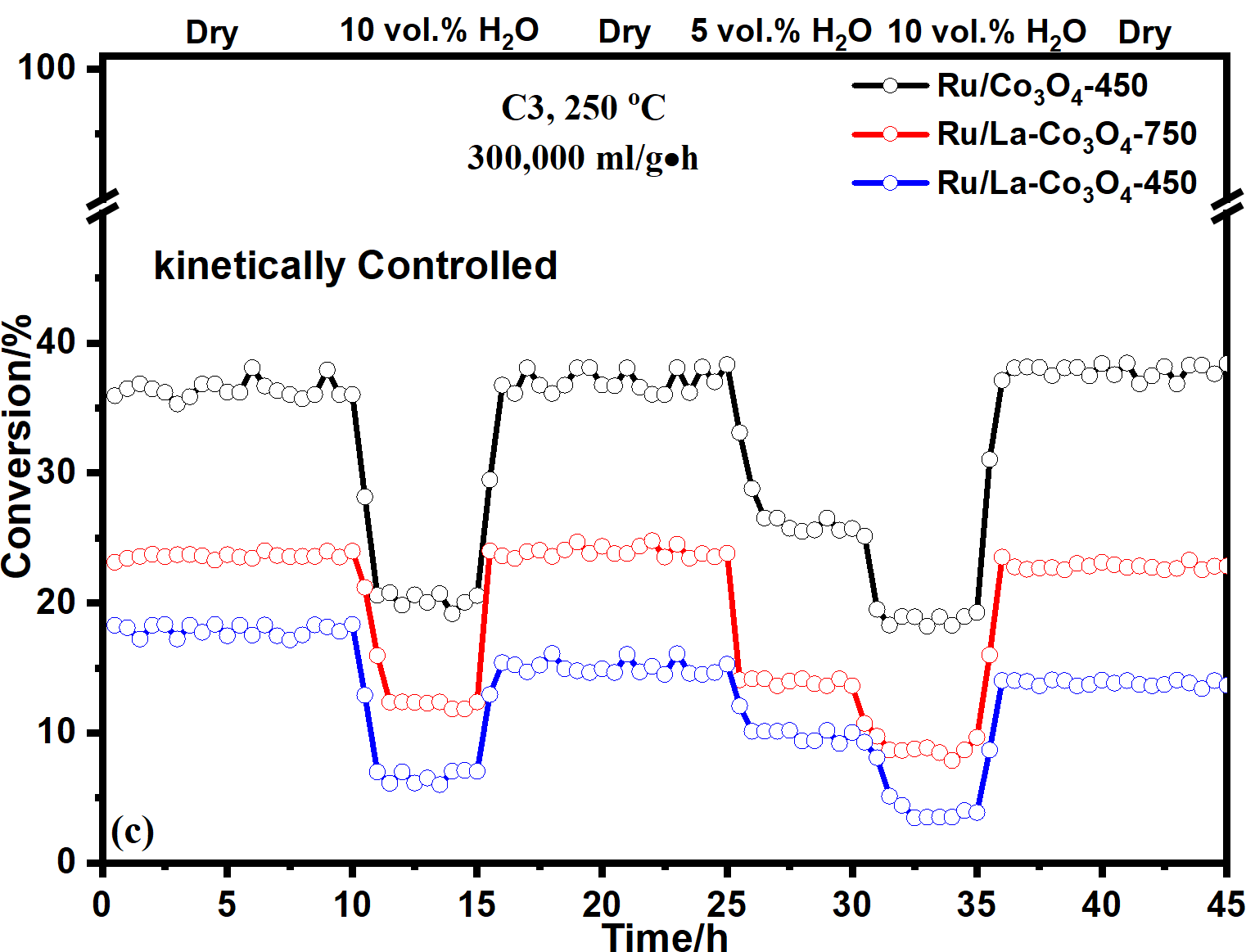


Figure S7 Long-term stability test of Ru/Co_3_O_4_-450, Ru/La-Co_3_O_4_-450, and Ru/La-Co_3_O_4_-750 at 250 °C and 300,000 ml/g·h in the absence and presence of 10.0 vol.% or 5.0 vol.% H_2_O under kinetically controlled conditions.


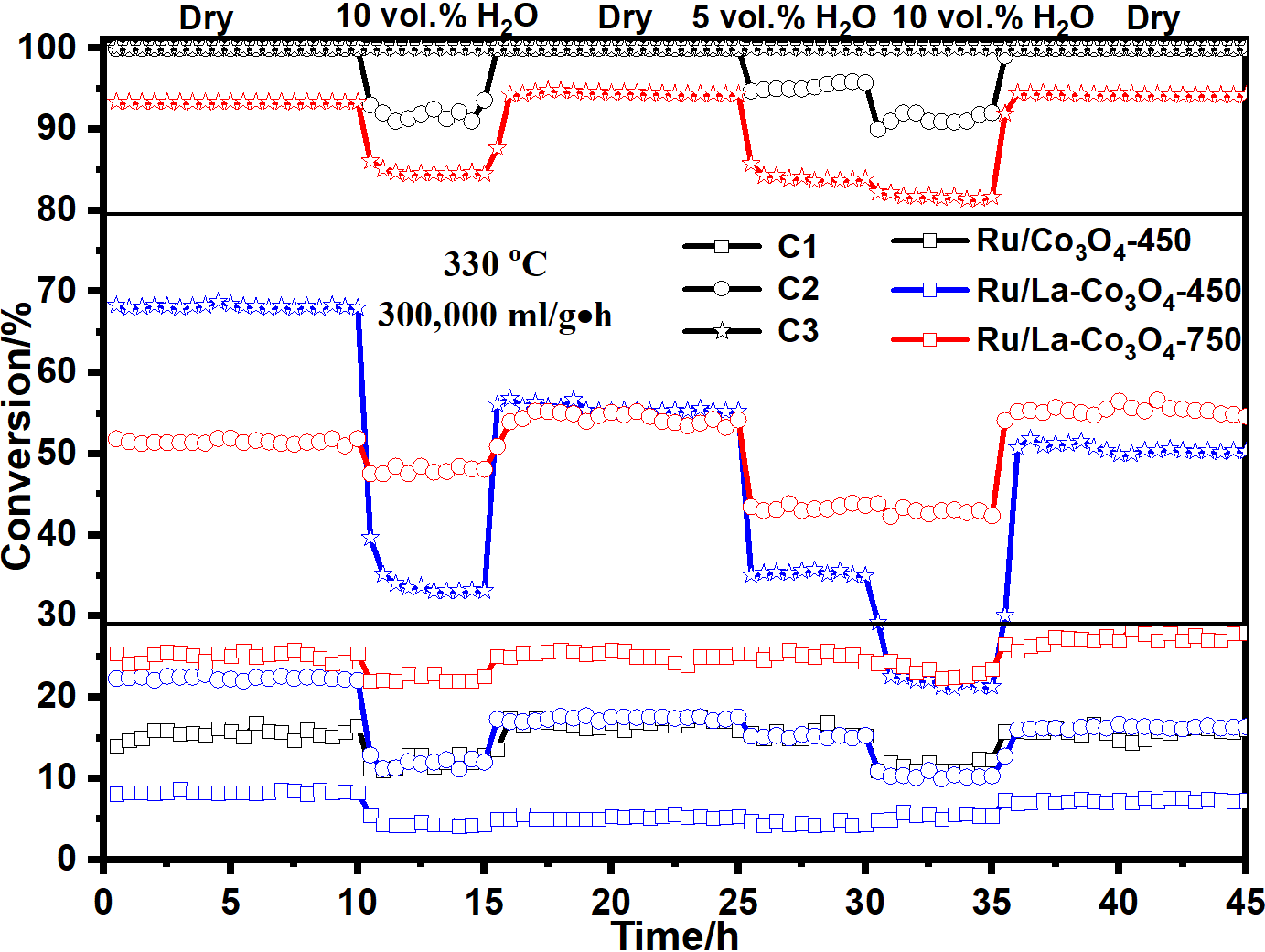


Figure S8 Long-term stability test of Ru/Co_3_O_4_-450, Ru/La-Co_3_O_4_-450, and Ru/La-Co_3_O_4_-750 at 330 °C and 300,000 ml/g·h in the absence and presence of 10.0 vol.% or 5.0 vol.% H_2_O.


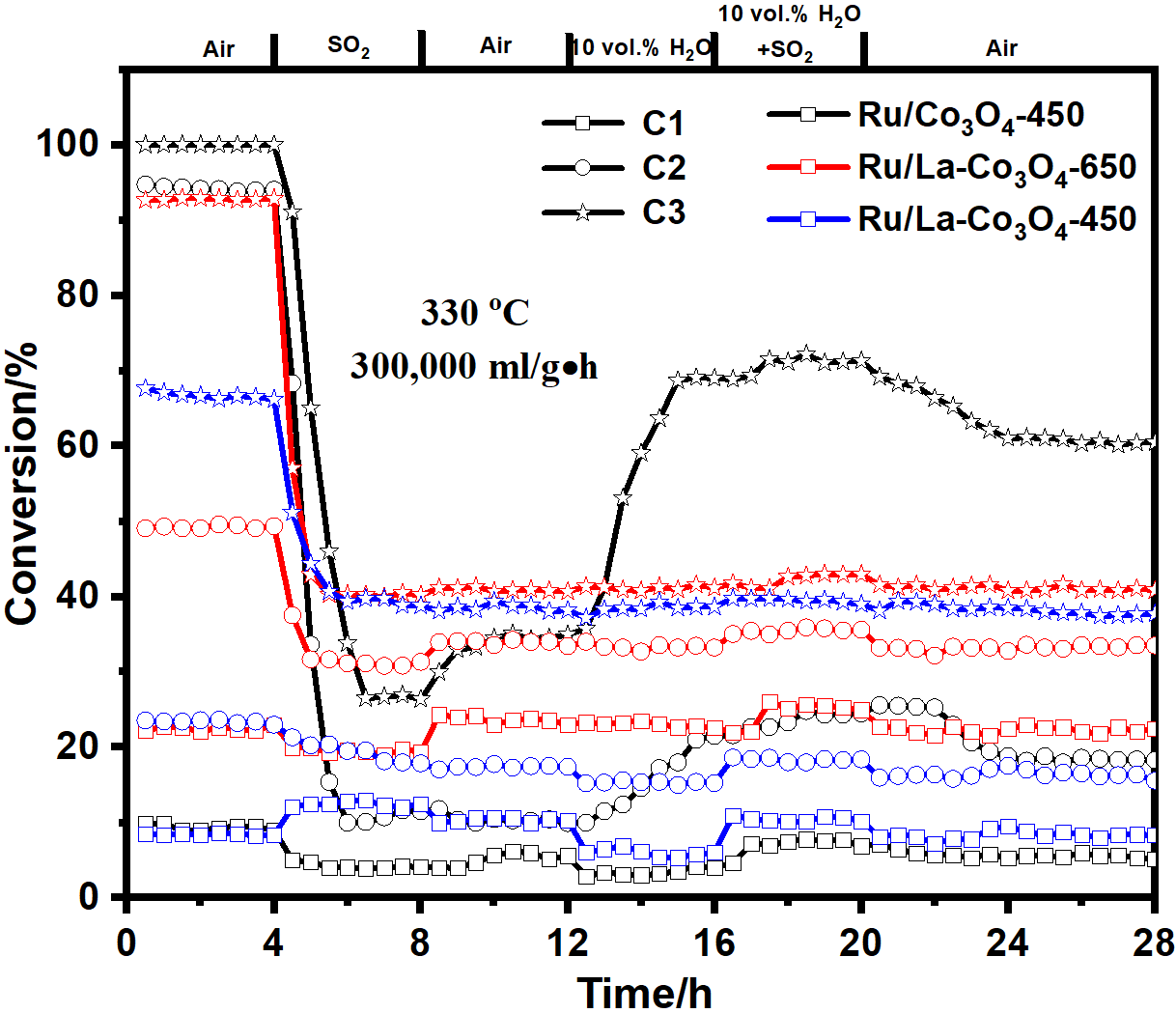


Figure S9 Effects of H_2_O (10.0 vol.%) and SO_2_ (20 ppm) on the stability of Ru/Co_3_O_4_-450, Ru/La-Co_3_O_4_-450 and Ru/La-Co_3_O_4_-650 at 330 °C and 300,000 ml/g·h.


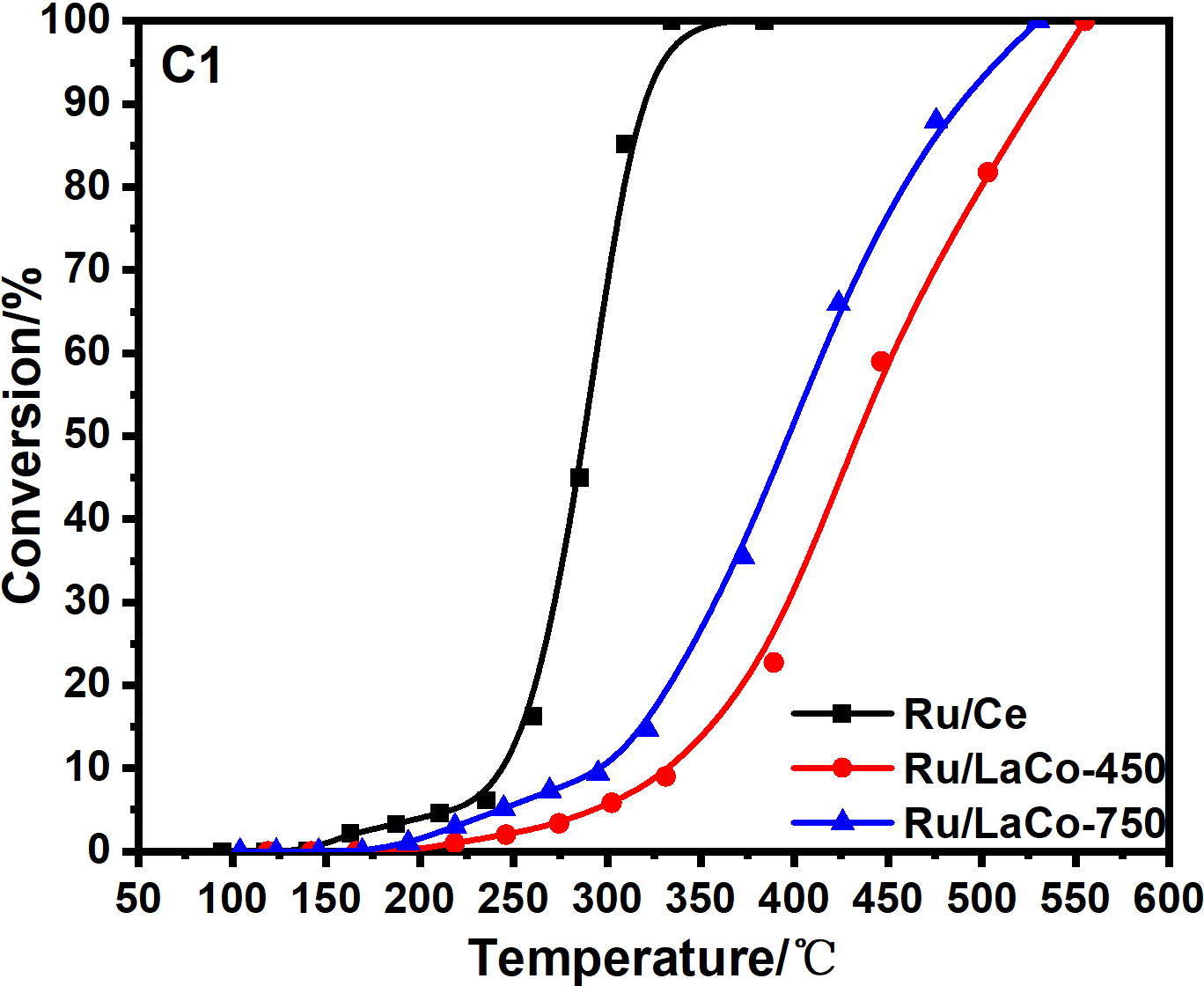


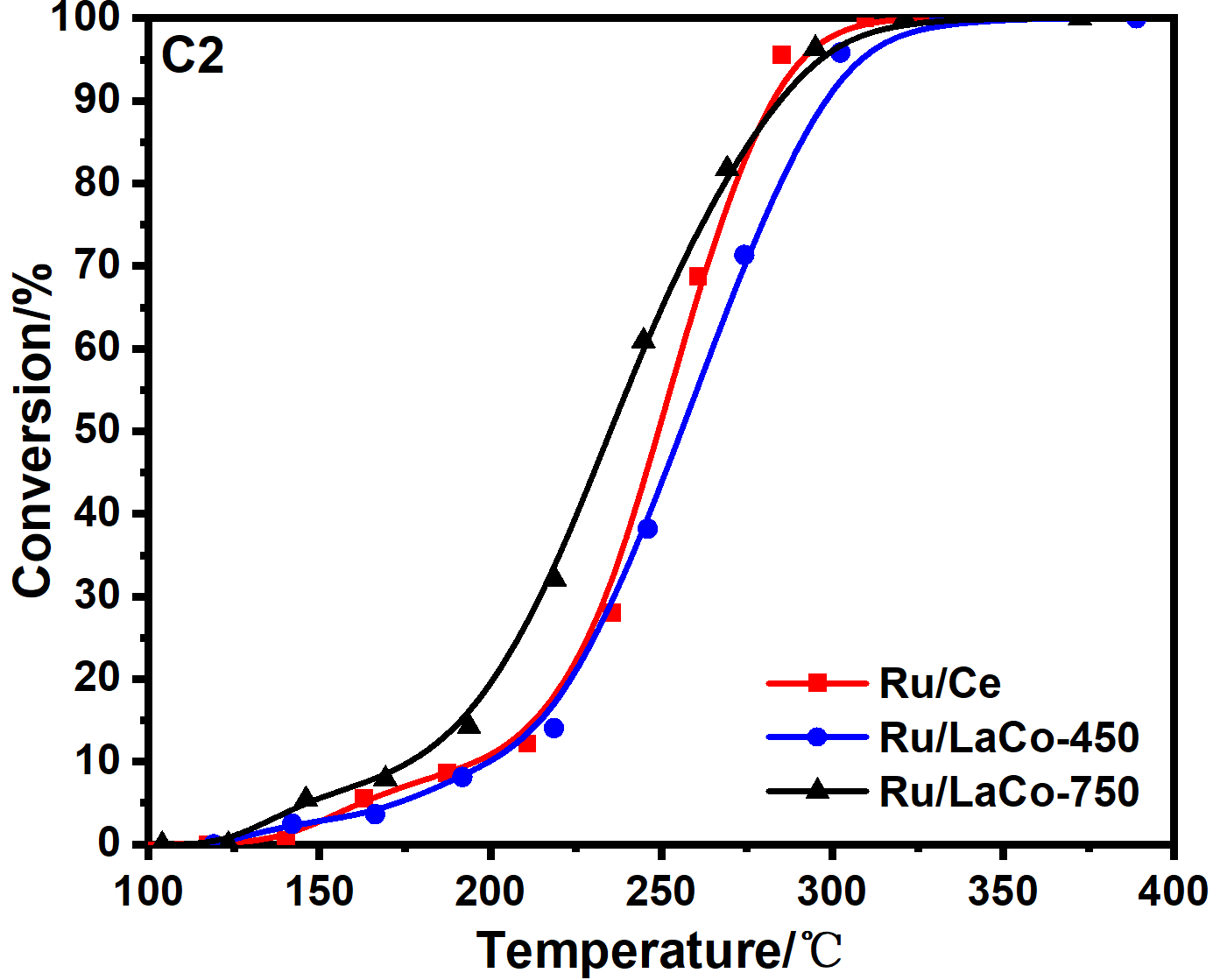


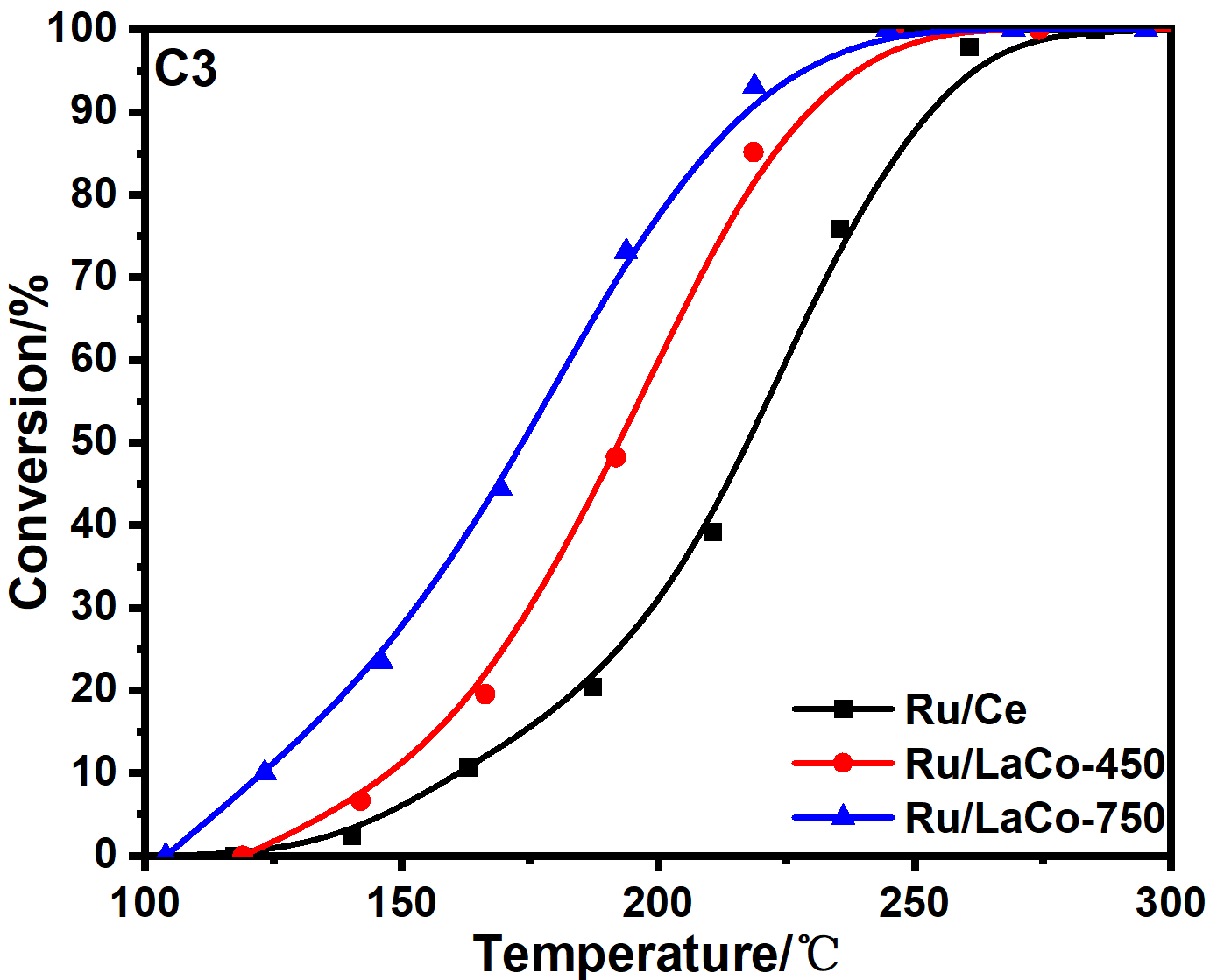


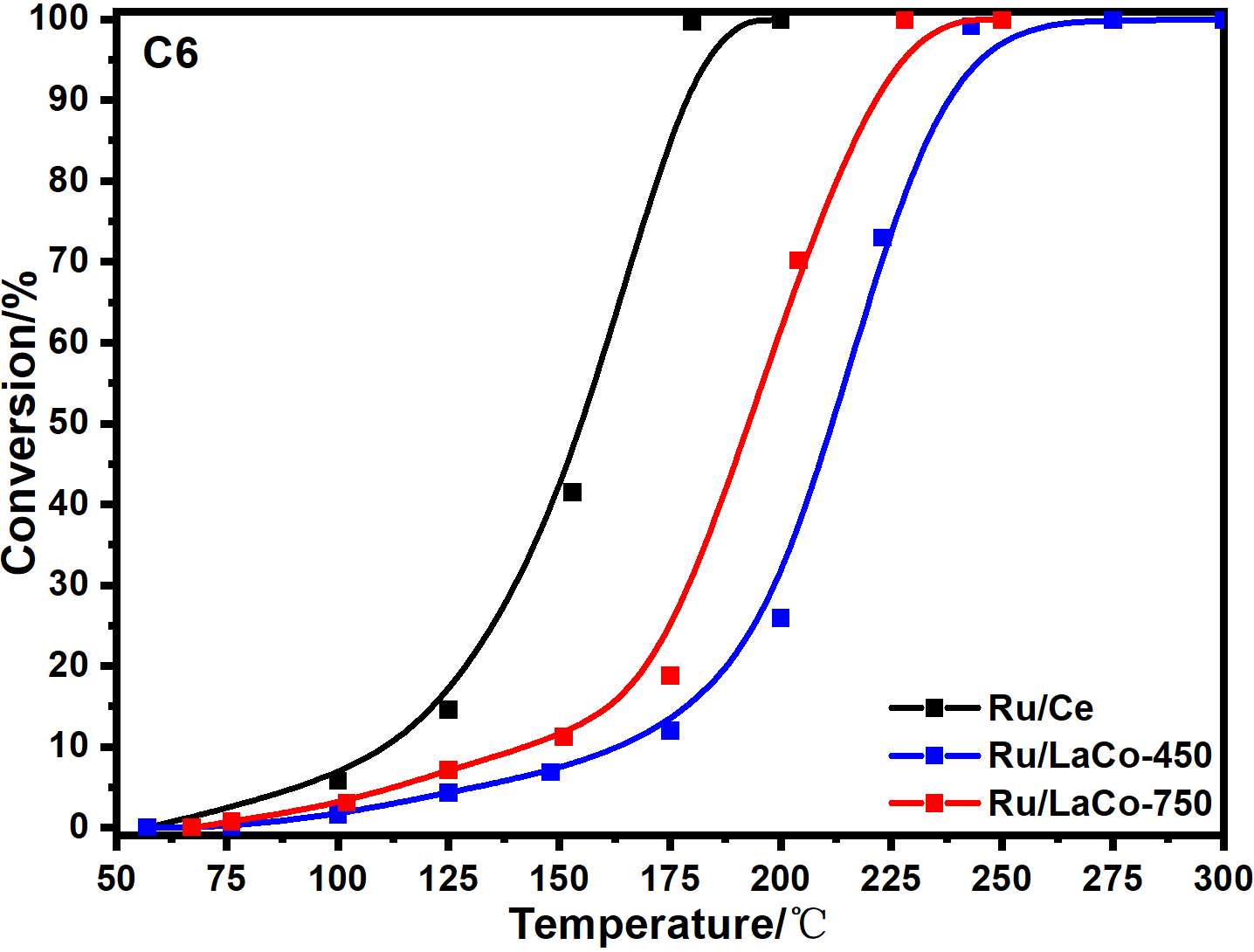


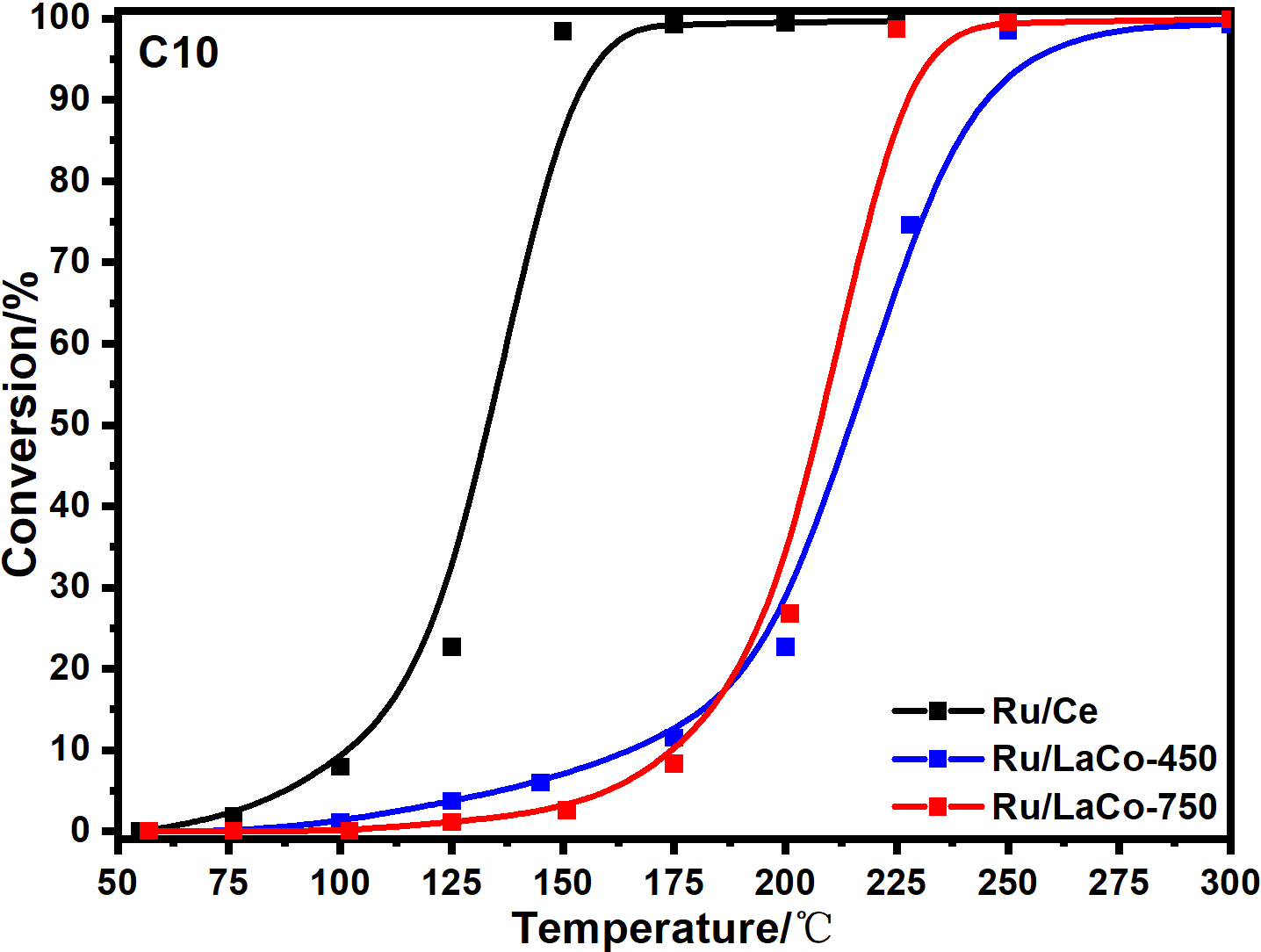


Figure S10 Light-off curves of Ru/La-Co_3_O_4_-450, Ru/La-Co_3_O_4_-750, and Ru/CeO_2_-450 catalysts for catalytic combustion of alkanes with different carbon chain lengths.


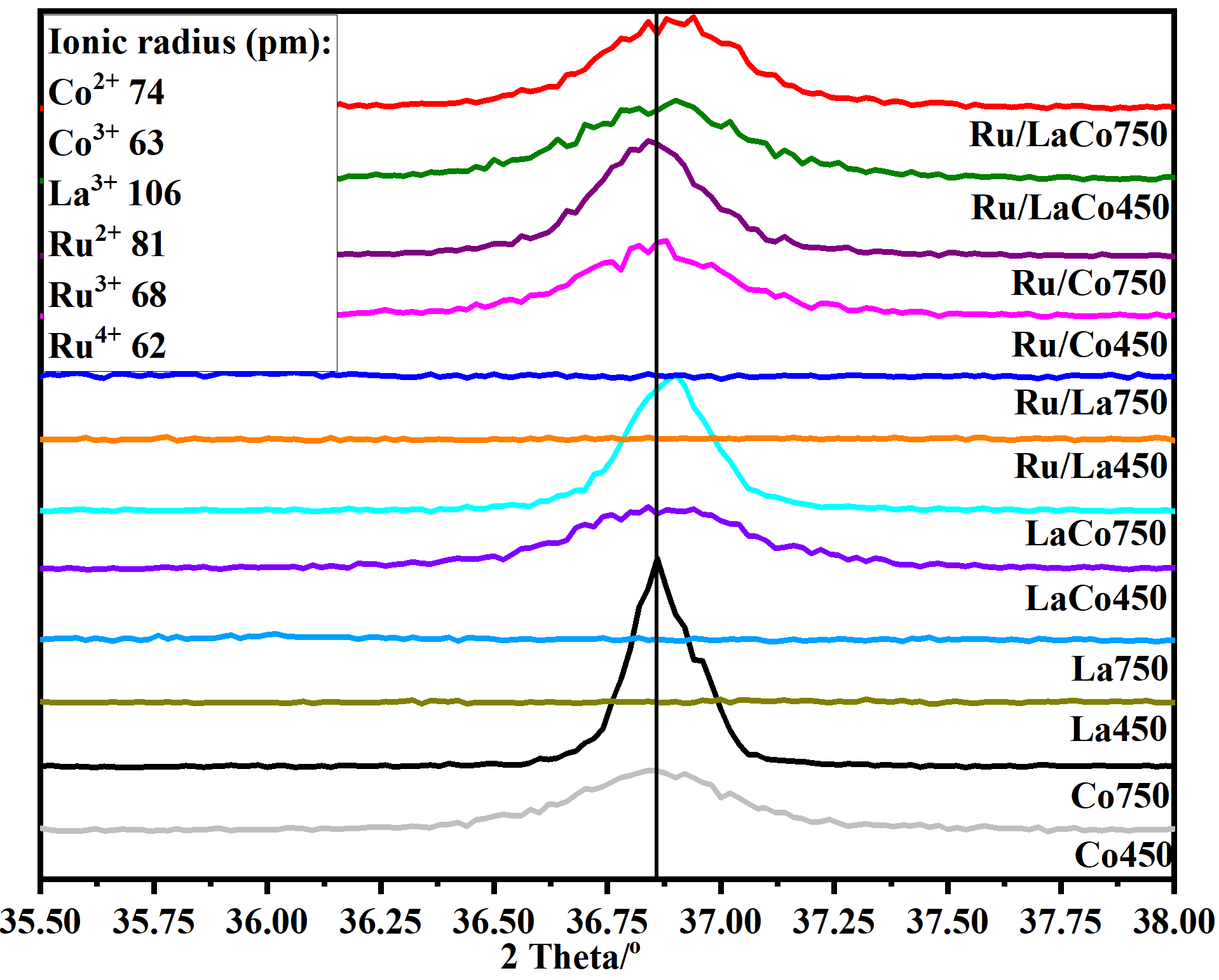


Figure S11 The enlarged XRD patterns of fresh and aged Co_3_O_4_, La_2_O_3_, La-Co_3_O_4_, Ru/La_2_O_3_, Ru/Co_3_O_4_, and Ru/La-Co_3_O_4_ catalysts.


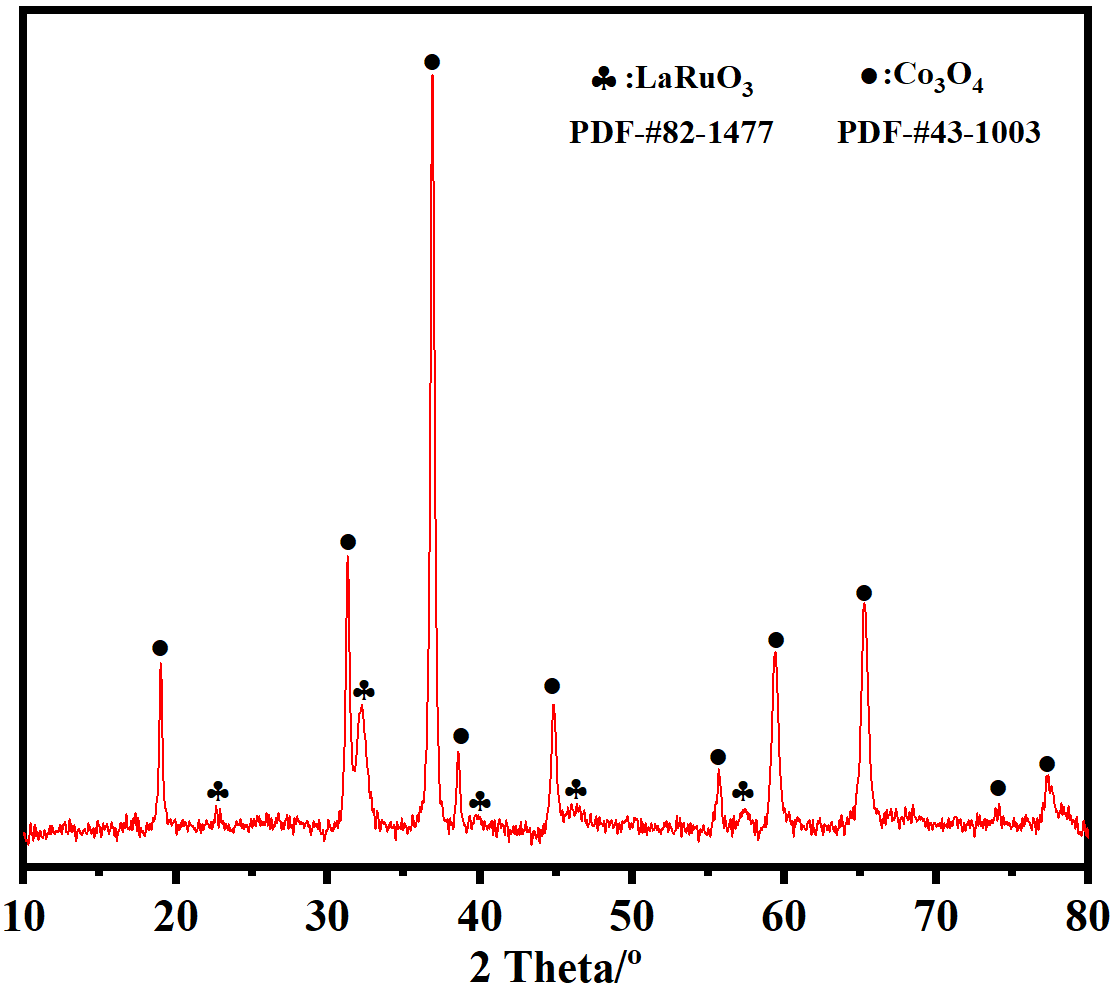


Figure S12 The XRD pattern (2°/min) of Ru/La-Co_3_O_4_-750 catalyst.


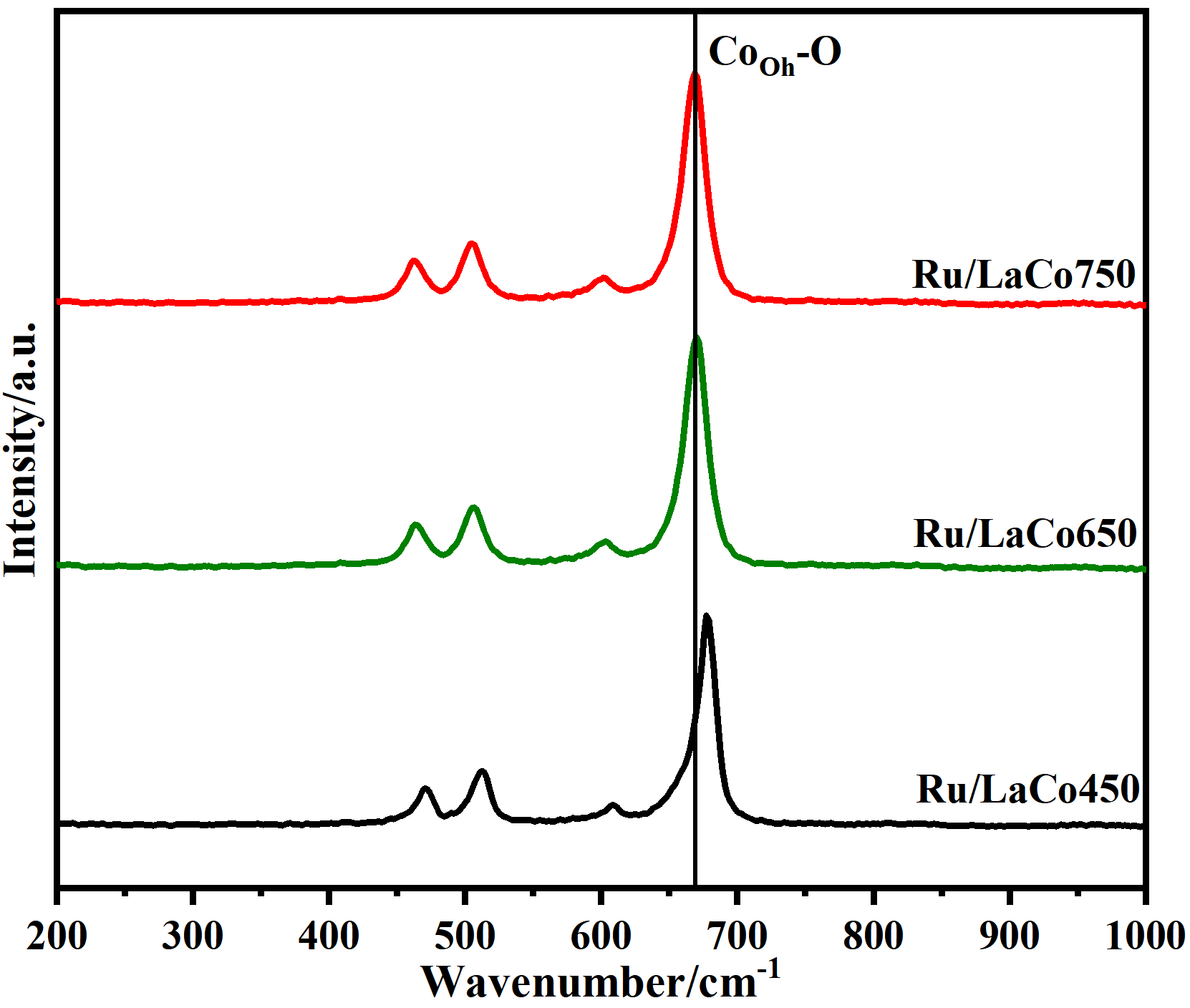


Figure S13 The enlarged Raman patterns of Ru/La-Co_3_O_4_-450, Ru/La-Co_3_O_4_-650, and Ru/La-Co_3_O_4_-750 catalysts.


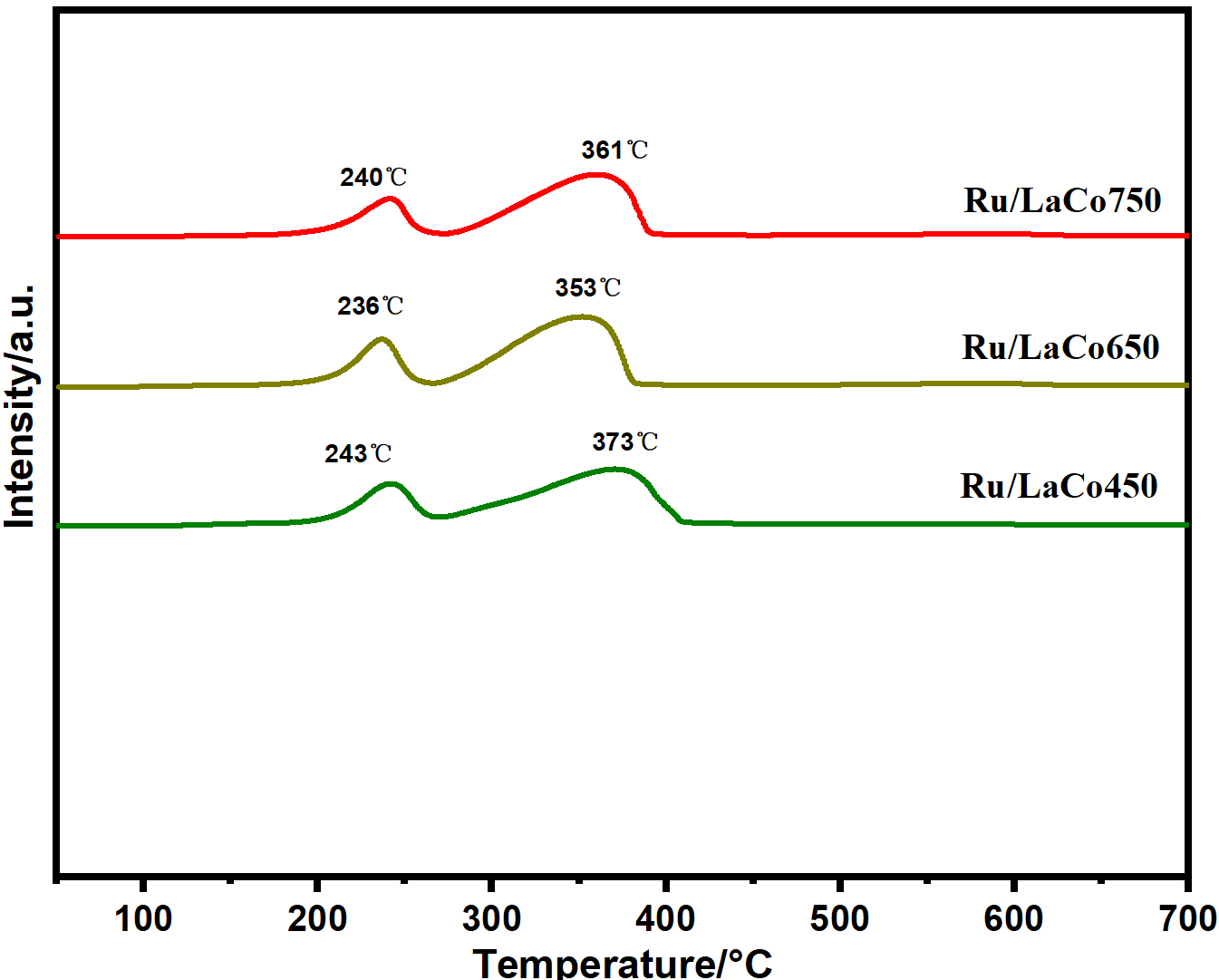


Figure S14 H_2_-TPR of Ru/La-Co_3_O_4_-450, Ru/La-Co_3_O_4_-650 and Ru/La-Co_3_O_4_-750 catalysts.


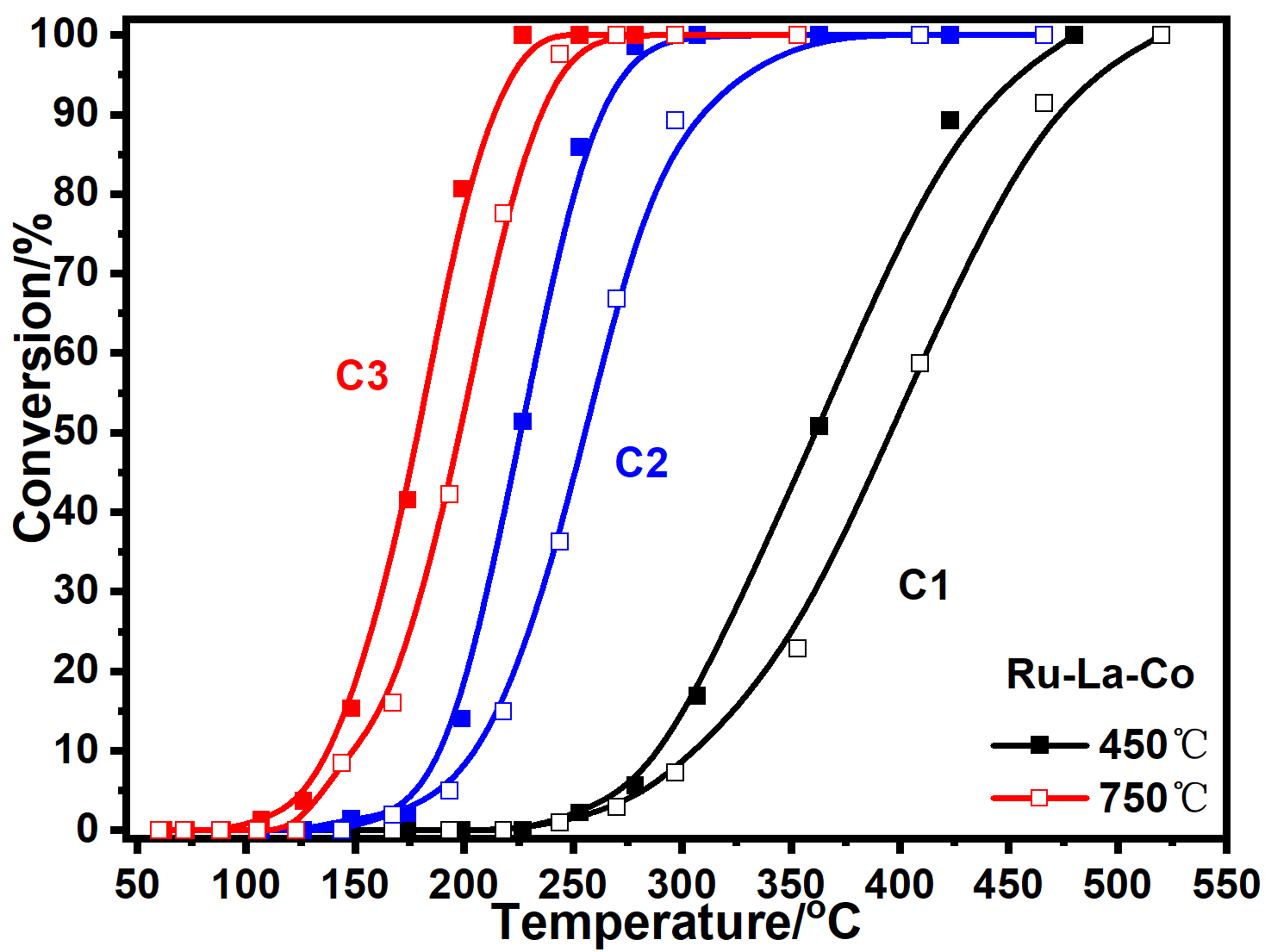


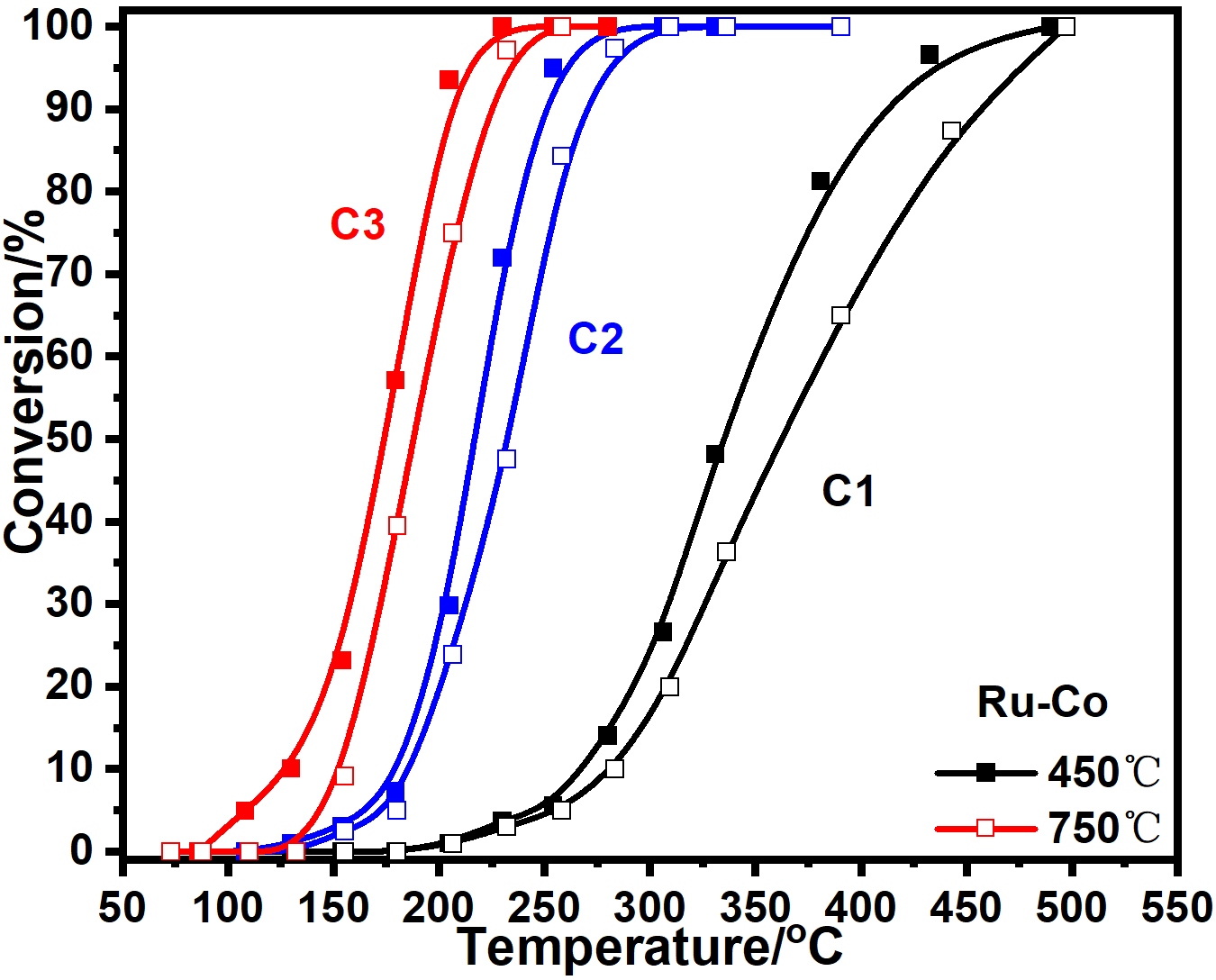


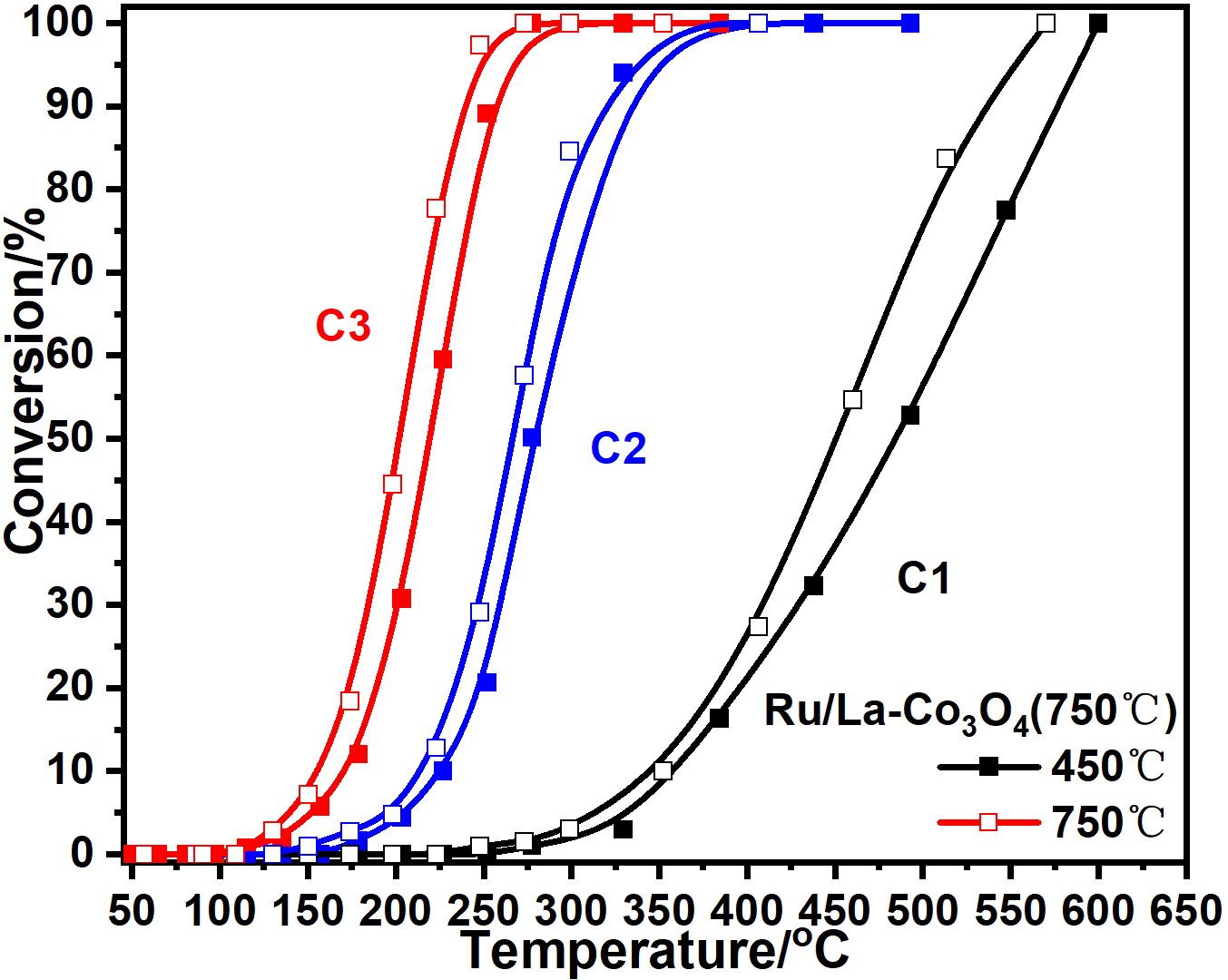


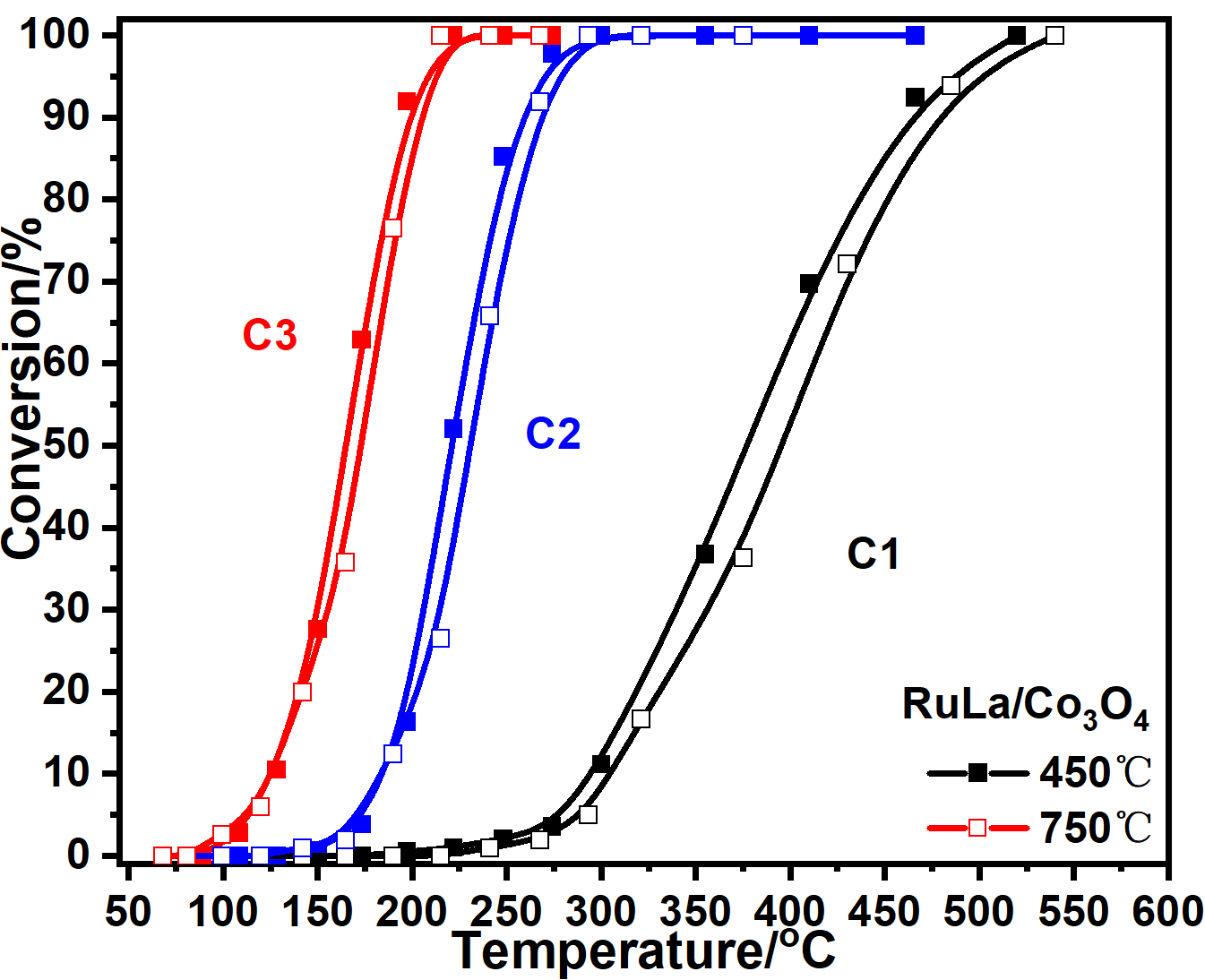


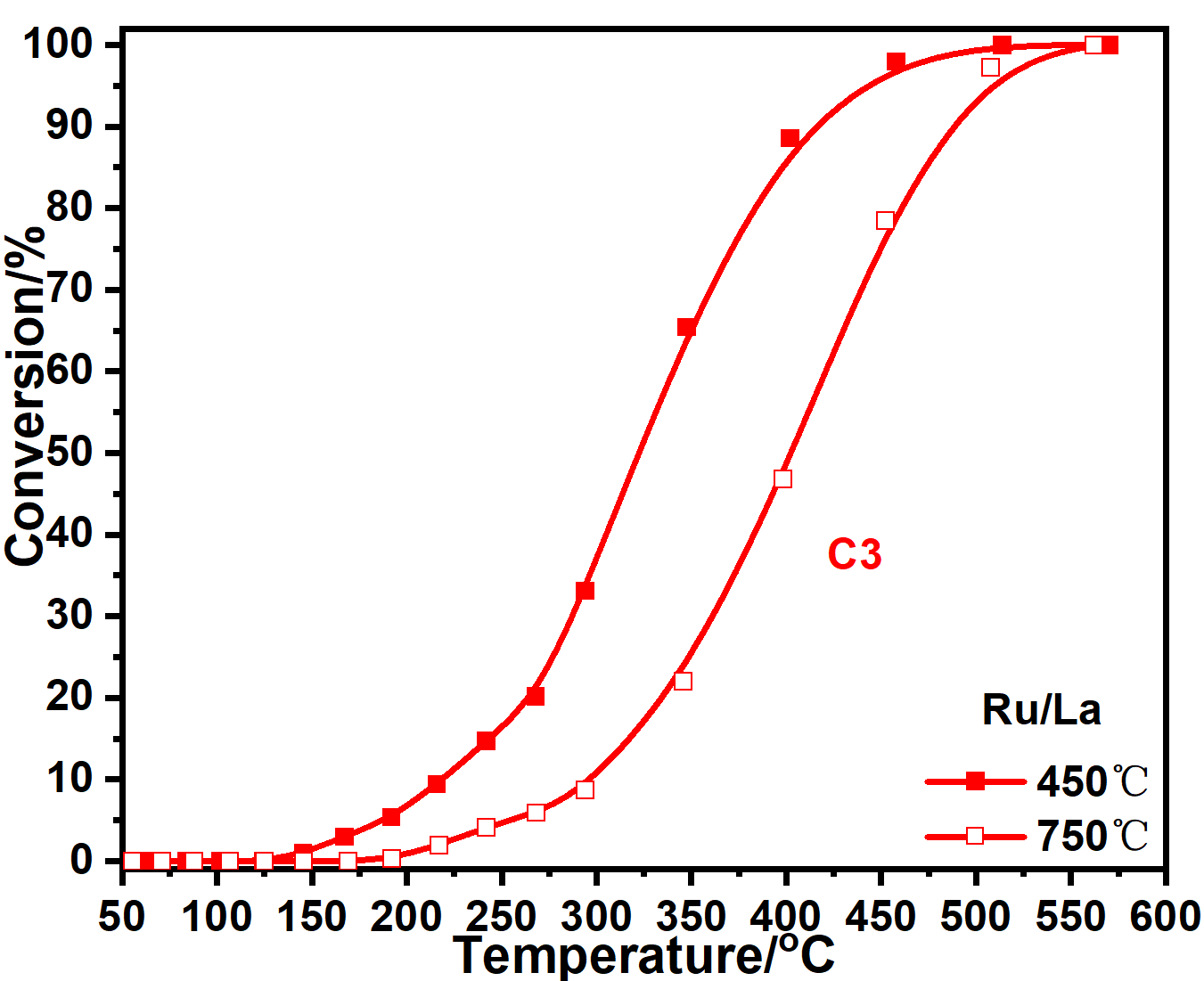


Figure S15 Light-off curves of fresh and aged RuLa-Co_3_O_4_, Ru-Co_3_O_4_, Ru/750La-Co_3_O_4_ (La-Co_3_O_4_ support was calcined at 750 °C), RuLa/Co_3_O_4_ and Ru/La_2_O_3_ catalysts for catalytic combustion of LHs.


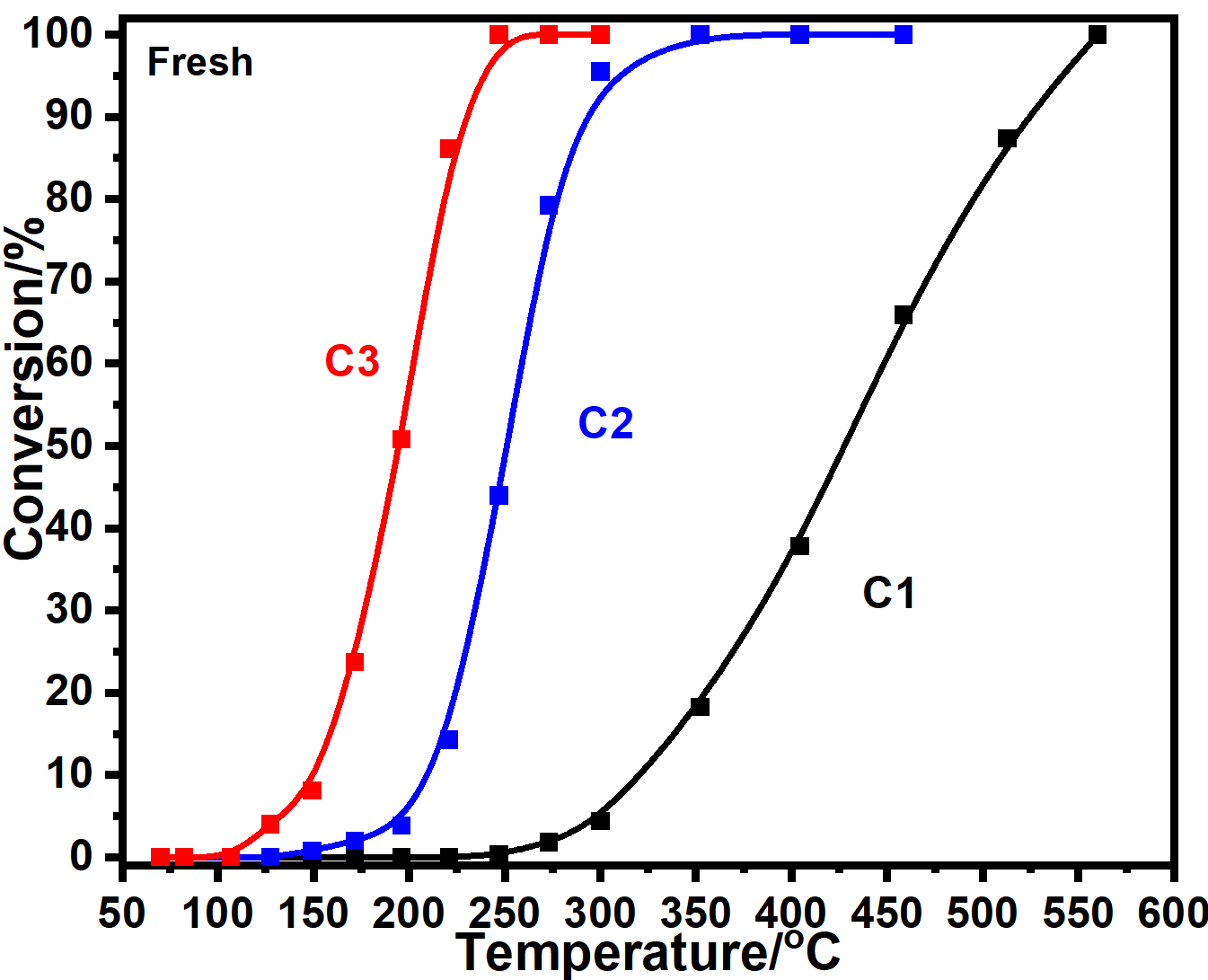


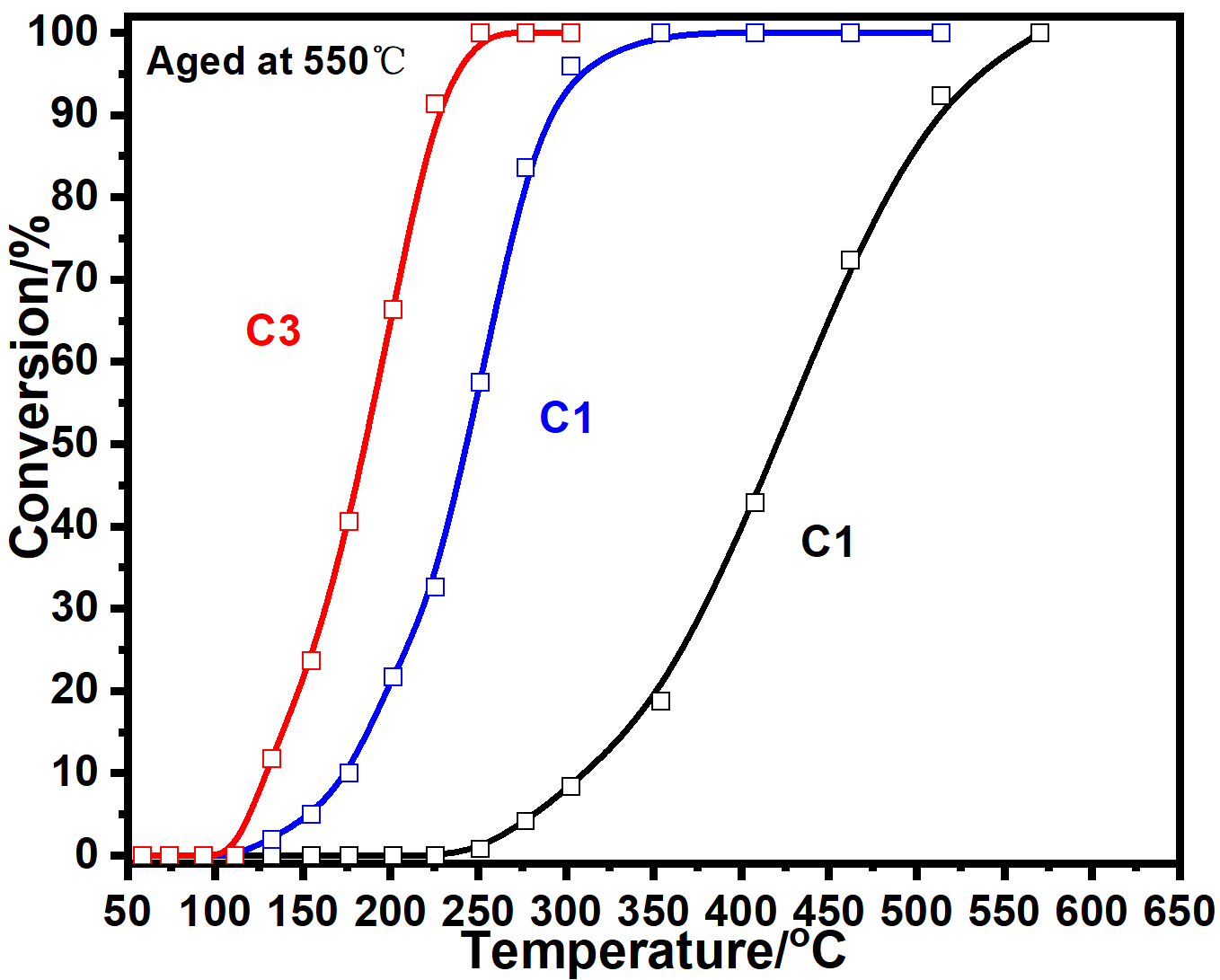


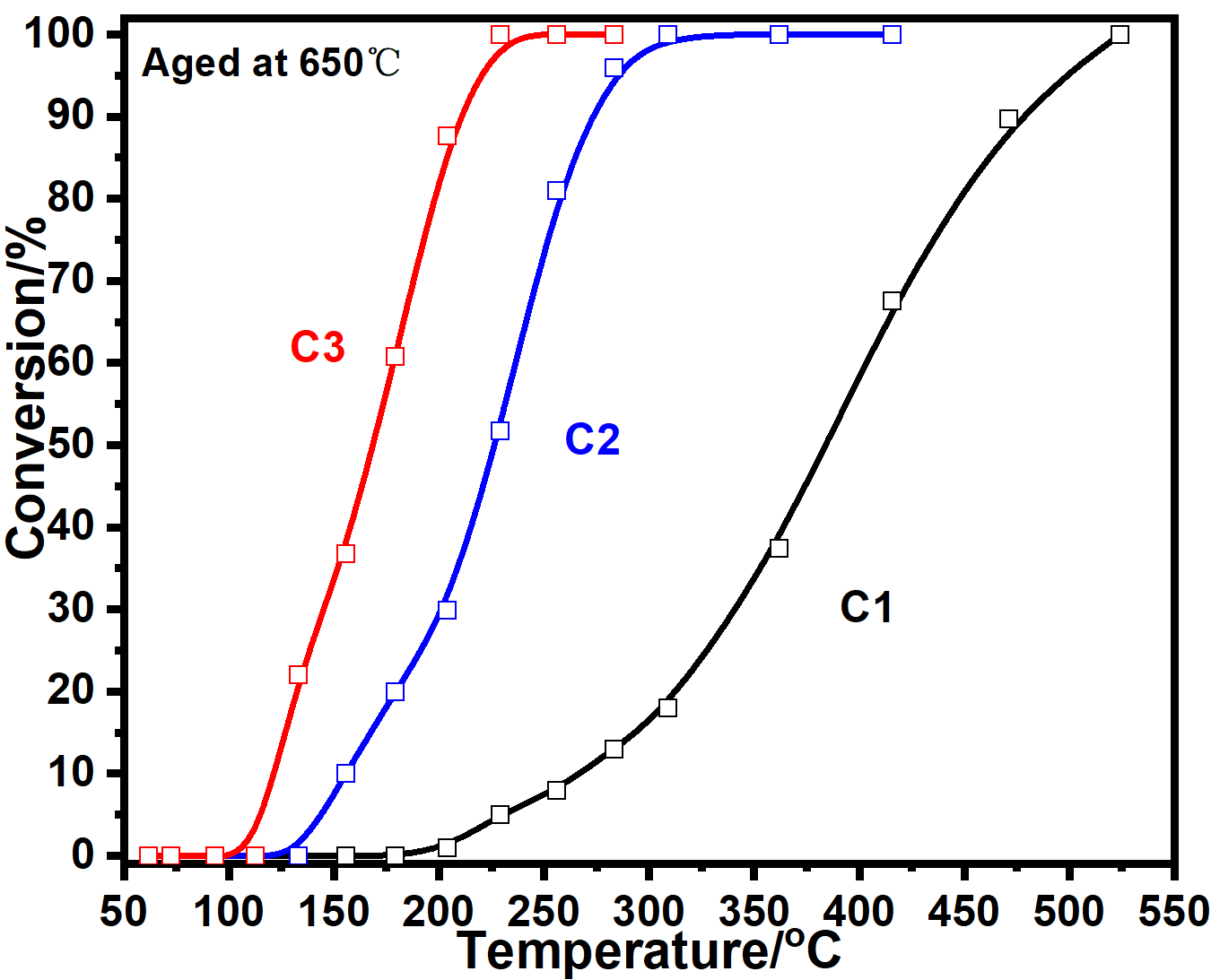


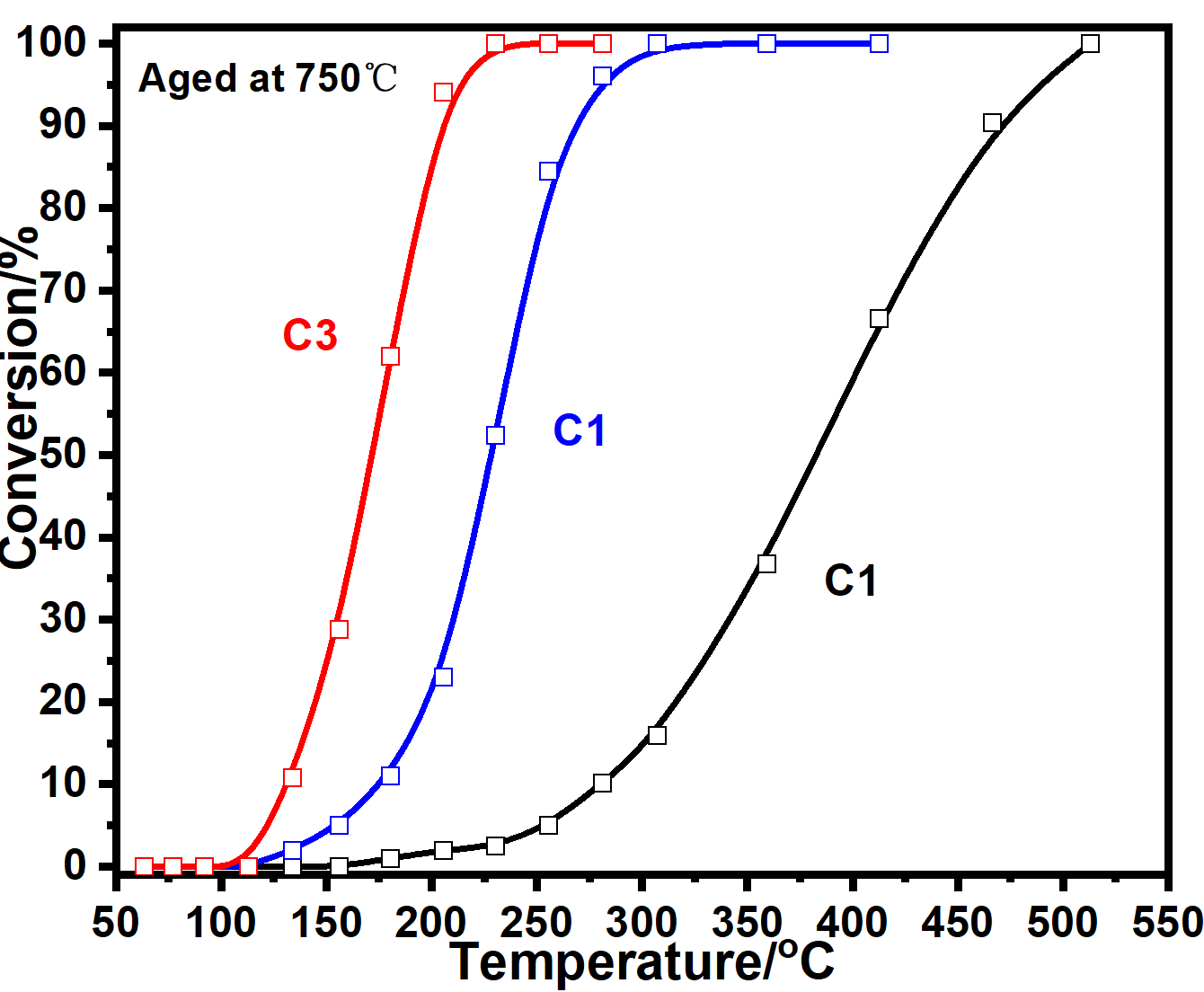


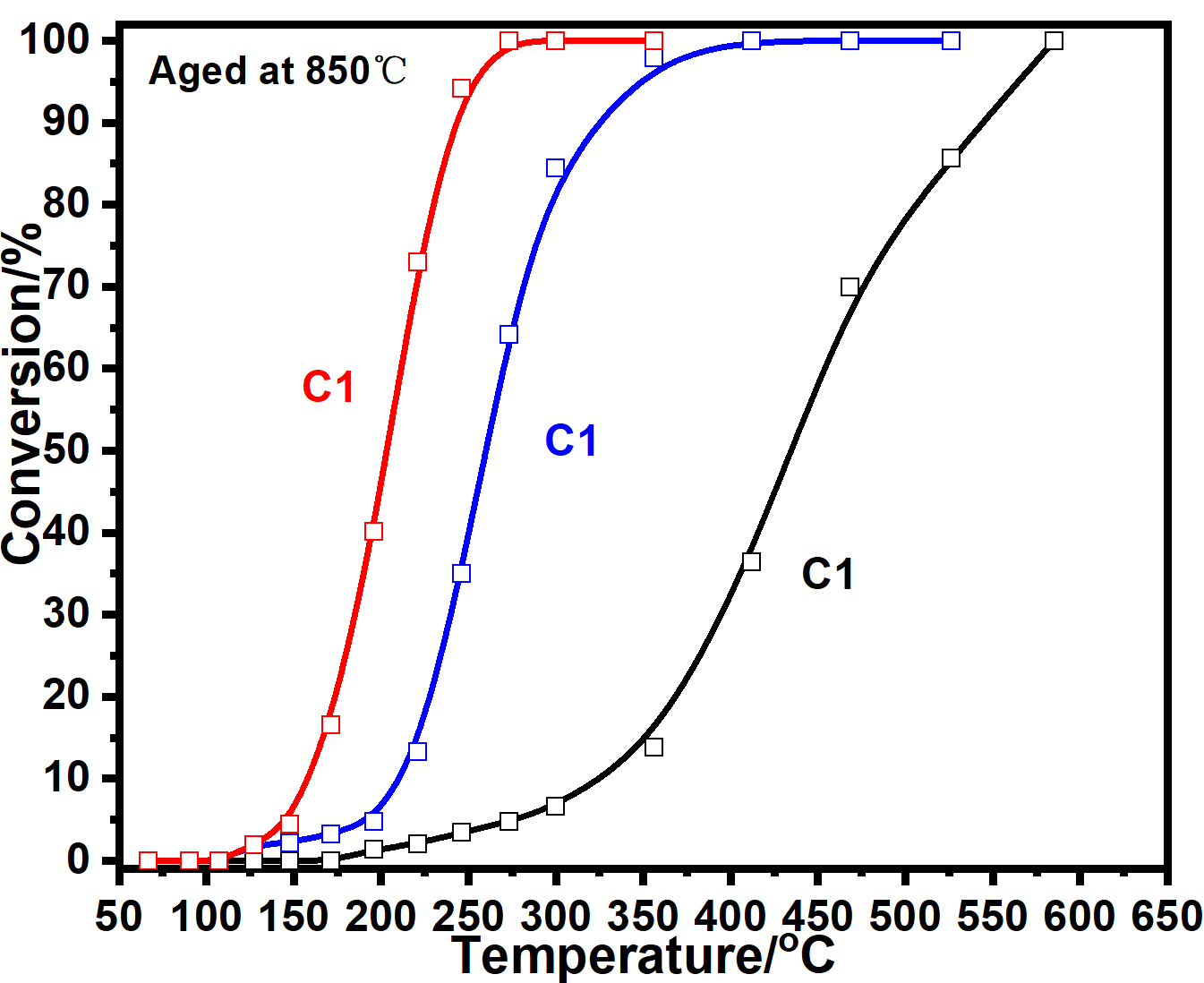


Figure S16 Light-off curves of fresh and aged Ru/La-Co_3_O_4_ at different temperatures for catalytic combustion of LHs.


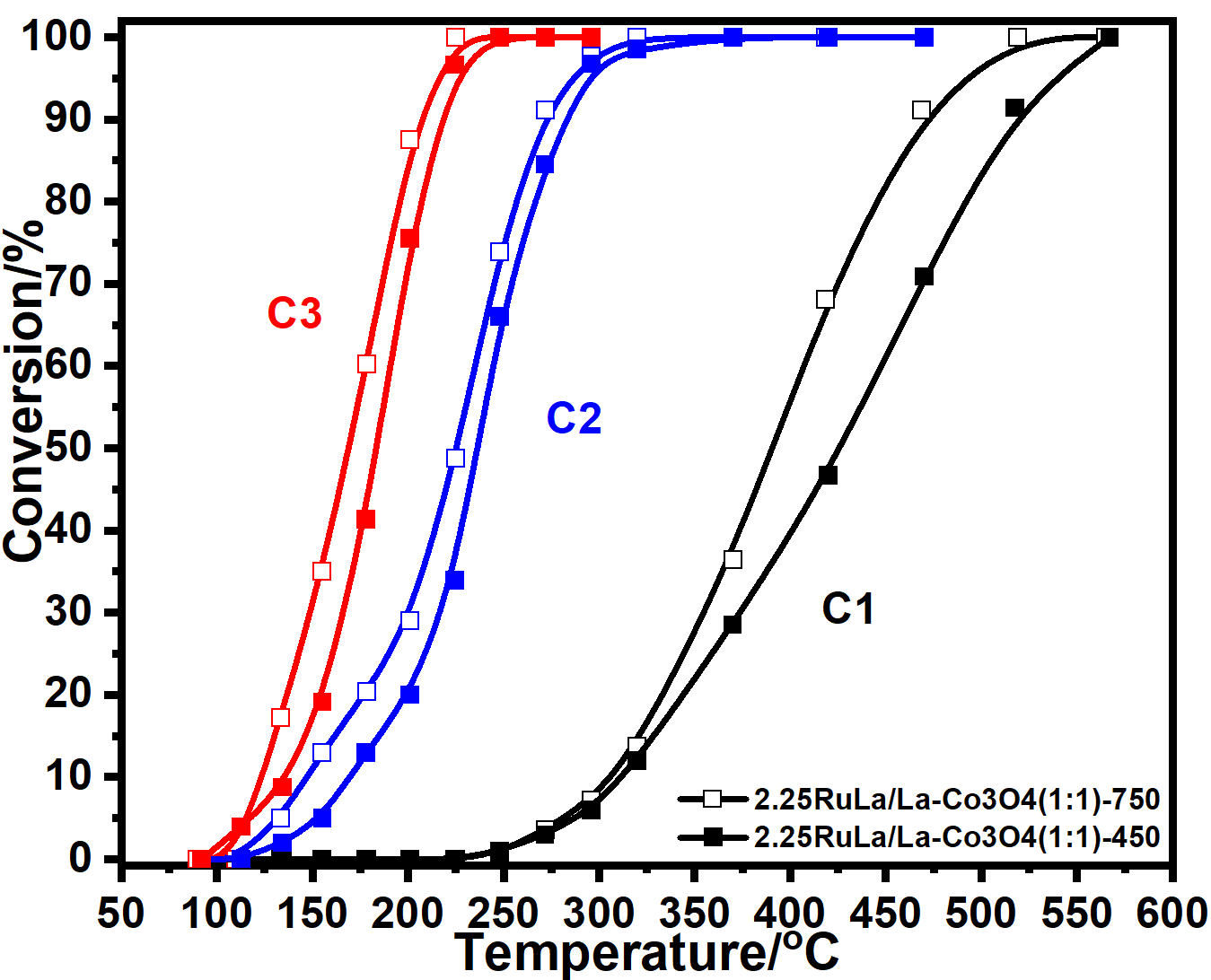

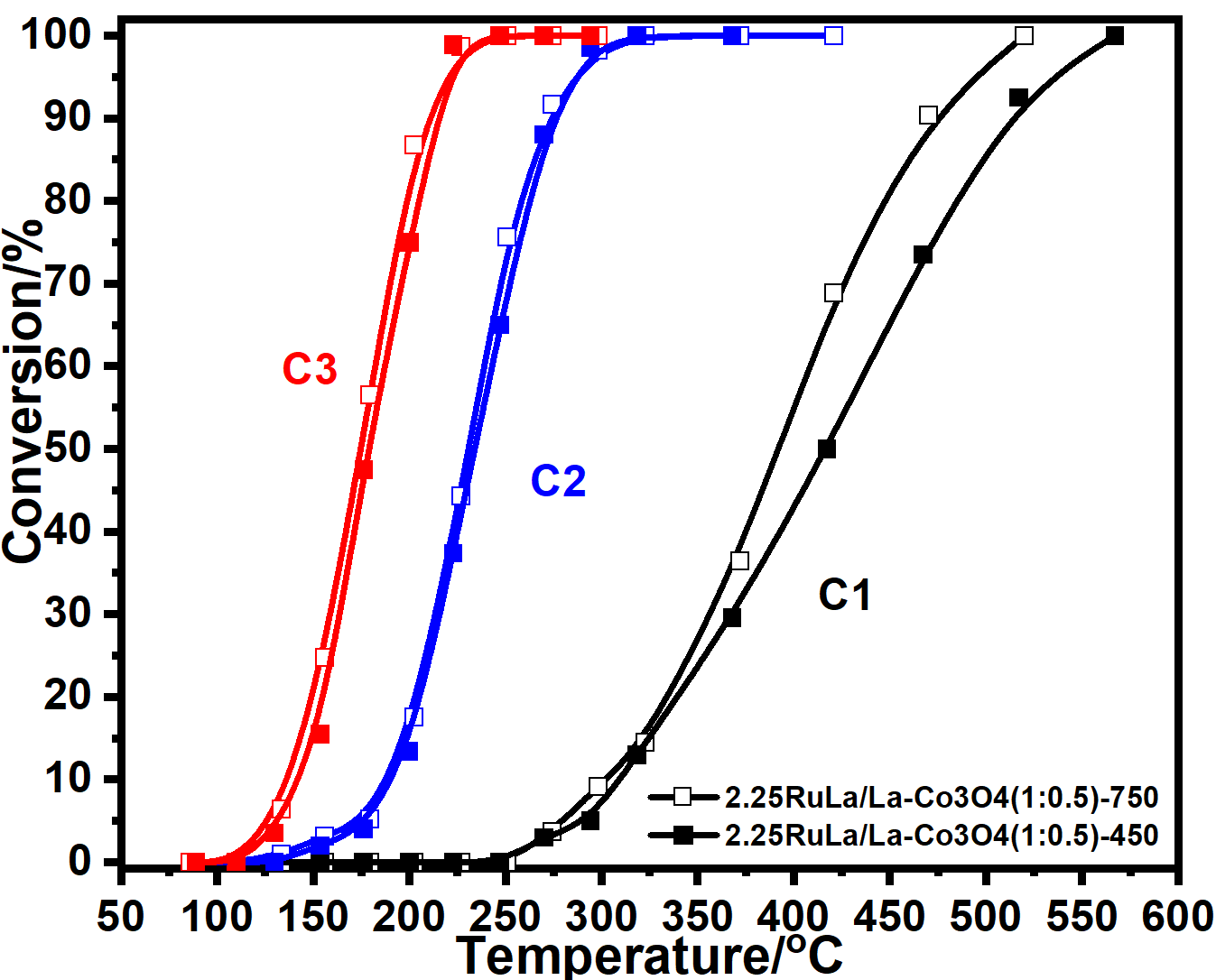


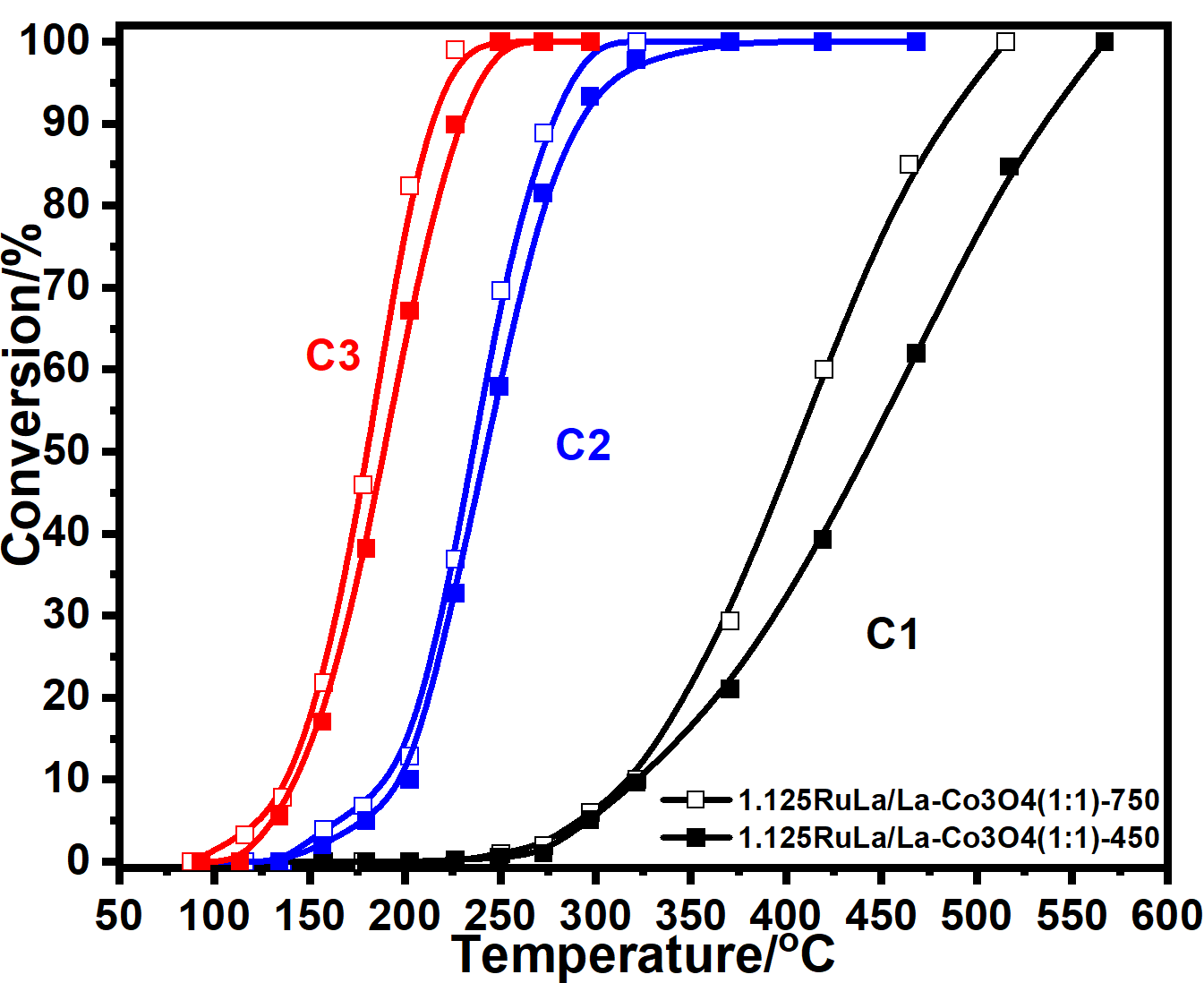

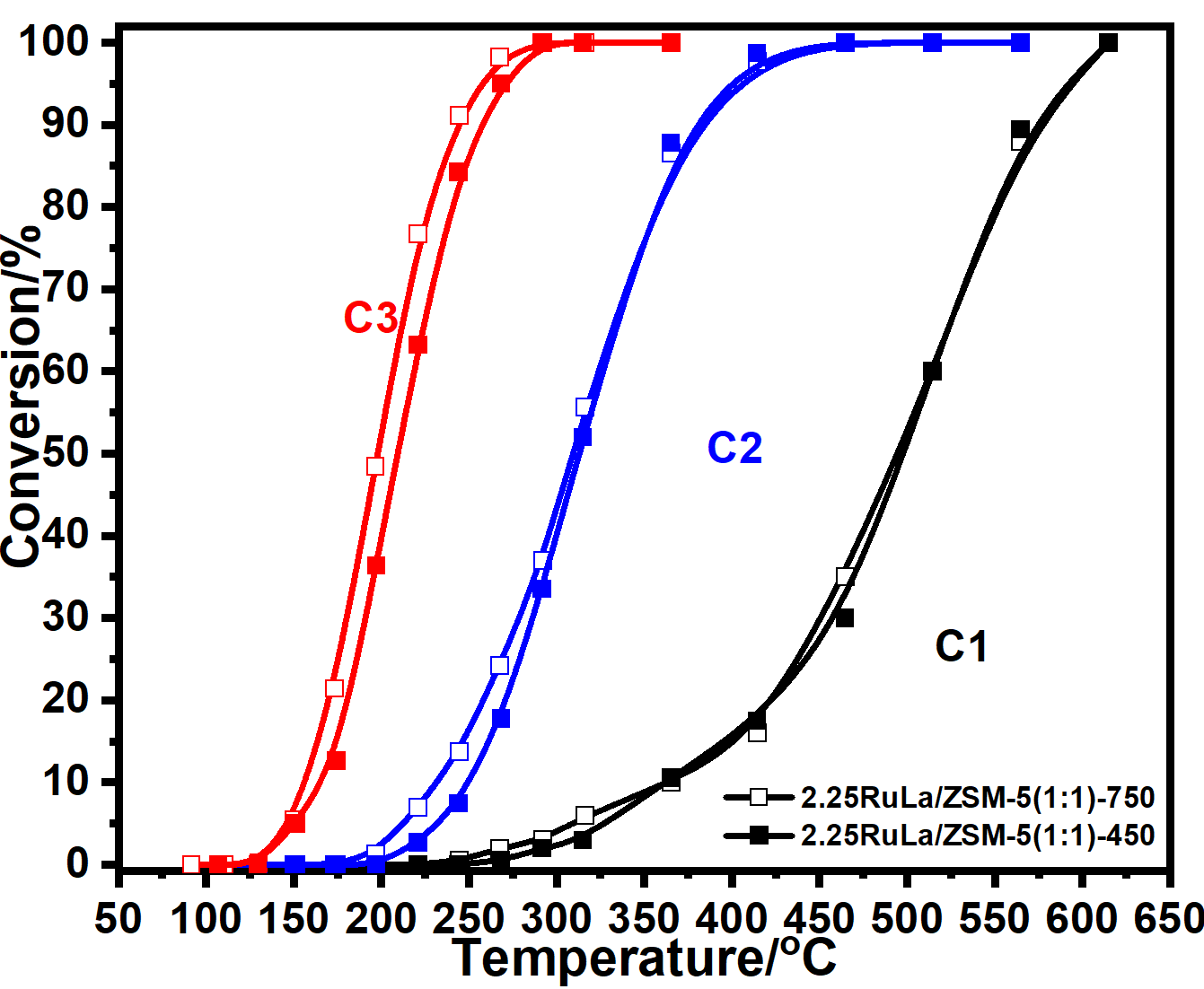


Figure S17 Light-off curves of fresh and aged Ru/La-Co_3_O_4_, RuLa/La-Co_3_O_4_ (5%La-Co_3_O_4_ as support, the loading of Ru and La as follows: RuLa/LaCo-1) 2.25 wt.% Ru and the mole ratio of La/Ru is 1, RuLa/LaCo-2) 2.25 wt.% Ru and the mole ratio of La/Ru is 0.5, and RuLa/LaCo-3) 1.125 wt.% Ru and the mole ratio of La/Ru is 1), RuLa/ZSM-500 for catalytic combustion of LHs.


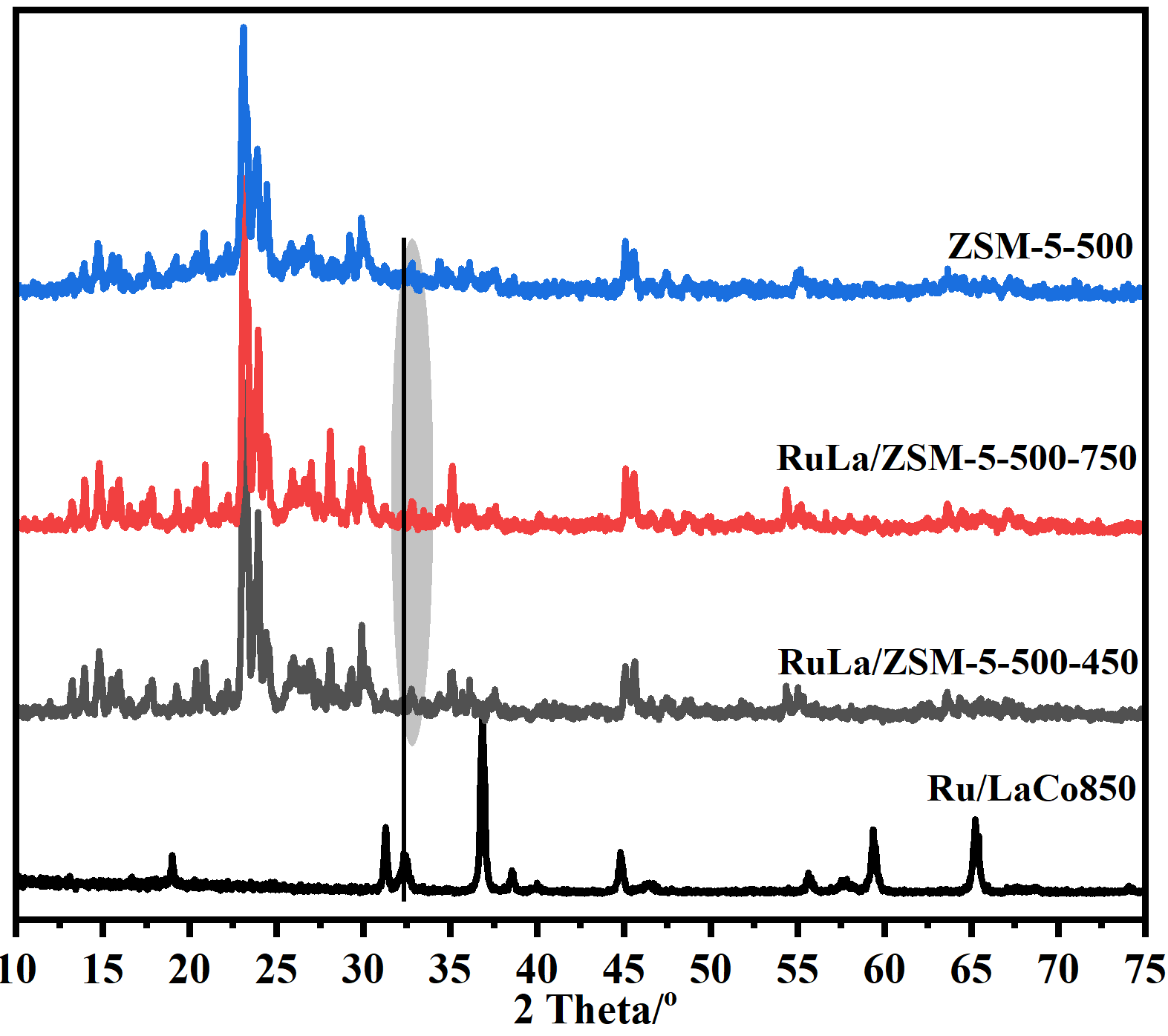


Figure S18 XRD patterns of Ru/LaCo-850, RuLa/ZSM-5-500-450, RuLa/ZSM-5-500-750 and ZSM-500.

Table S1 Chemicals and reagents used in the experiments

| reagents | molecular formula | standard | manufacturer |
| --- | --- | --- | --- |
| Cobalt acetate tetrahydrate | C_4_H_14_CoO_8_ | AR | Sinopharm Chemical Reagent Corporation |
| Cerium acetate hexahydrate | C_6_H_21_CeO_12_ | AR | Shanghai McLean Biochemical Technology Co |
| Lanthanum acetate tetrahydrate | C_6_H_17_LaO_10_ | AR | Sinopharm Chemical Reagent Corporation |
| Yttrium acetate | C_6_H_9_YO_6_ | AR | Shanghai McLean Biochemical Technology Co |
| Praseodymium acetate pentahydrate | C_6_H_19_PrO_11_ | AR | Shanghai McLean Biochemical Technology Co |
| Samarium acetate pentahydrate | C_6_H_19_SmO_11_ | AR | Shanghai McLean Biochemical Technology Co |
| Neodymium acetate pentahydrate | C_6_H_19_NdO_11_ | AR | Shanghai McLean Biochemical Technology Co |
| Gadolinium acetate trihydrate | C_6_H_11_GdO_7_ | AR | Shanghai McLean Biochemical Technology Co |
| Ruthenium nitrosyl nitrate | Ru(NO)(NO_3_)_3_ | AR | Shanghai Praseodymium Strontium New Material Technology Co |
| Platinum nitrate | Pt(NO_3_)_2_ | AR | Shanghai Praseodymium Strontium New Material Technology Co |
| silver nitrate | AgNO_3_ | AR | Shanghai Praseodymium Strontium New Material Technology Co |
| palladium nitrate | Pd(NO_3_)_2_ | AR | Shanghai Praseodymium Strontium New Material Technology Co |
| deionized water | H_2_O | ≥99.9% | self-restraint |

Table S2 SSA of the synthesized catalysts

| samples | SSA(g/m^2^) |
| --- | --- |
| Ru/Co_3_O_4_-450 | 23 |
| Ru/Co_3_O_4_-750 | 6 |
| Ru/La-Co_3_O_4_-450 | 24 |
| Ru/La-Co_3_O_4_-750 | 12 |

Table S3 XPS data of the synthesized catalysts

| **samples** | **Co^3+^/Co^2+^** | **Ru^4+^/Ru^n+^** |
| --- | --- | --- |
| **Ru/Co_3_O_4_-450** | 0.64 | 0.45 |
| **Ru/Co_3_O_4_-750** | 0.50 | 1.42 |
| **Ru/La-Co_3_O_4_-450** | 0.43 | 0.41 |
| **Ru/La-Co_3_O_4_-750** | 0.54 | 0.32 |

Table S4 Curve fit parameters for Co K-edge EXAFS for Co_3_O_4_, La-Co_3_O_4_, Ru/ La-Co_3_O_4_-450 and Ru/La-Co_3_O_4_-750.(S_0_^2^ of 0.72 was obtained from the experimental EXAFS fit of Co reference by fixing N as the known crystallographic value and was fixed to all samples.Data rangs:3≤k≤12.3 Å^-1^,1.0≤R≤3.5 Å . The number of variable parameters is 10, out of a total of 14.8 independent data points.)

| **Sample** | **Path** | ***N*** | ***R*/**Å | **ΔE_0_/eV** | **σ^2^/Å^2^** | **R-factor** |
| --- | --- | --- | --- | --- | --- | --- |
|  | Co-O | 5.9±0.3 | 1.91±0.01 |  | 0.004±0.001 |  |
| **Co_3_O_4_** | Co-Co1 | 5.9±0.8 | 2.87±0.01 | 4.7±0.6 | 0.005±0.001 | 0.002 |
|  | Co-Co2 | 9.8±1.5 | 3.34±0.01 |  | 0.007±0.001 |  |
|  | Co-O | 5.3±0.5 | 1.92±0.01 |  | 0.003±0.001 |  |
| **La-Co_3_O_4_** | Co-Co1 | 6.5±1.6 | 2.85±0.01 | 5.7±1.1 | 0.006±0.001 | 0.011 |
|  | Co-Co2 | 7.8±2.4 | 3.34±0.01 |  | 0.006±0.002 |  |
|  | Co-O | 5.7±0.5 | 1.92±0.01 |  | 0.003±0.001 |  |
| **Ru/ La-Co_3_O_4_-450** | Co-Co1 | 6.0±1.6 | 2.85±0.01 | 5.0±1.2 | 0.006±0.01 | 0.001 |
|  | Co-Co2 | 8.5±2.8 | 3.34±0.01 |  | 0.007±0.002 |  |
|  | Co-O | 5.86±0.3 | 1.92±0.01 |  | 0.002±0.001 |  |
| **Ru/ La-Co_3_O_4_-750** | Co-Co1 | 6.1±1.0 | 2.85±0.01 | 5.0±0.7 | 0.005±0.001 | 0.002 |
|  | Co-Co2 | 9.6±1.7 | 3.34±0.01 |  | 0.007±0.001 |  |
